# CLN7 suppression induces apoptosis via mTOR-regulated and chaperone-mediated autophagy in myeloid leukemia cells

**DOI:** 10.1038/s41419-026-08936-2

**Published:** 2026-05-30

**Authors:** Miaomiao Wu, Hui Li, Sujun Li, Qianwen Xu, Yijing Liao, Lili Qu, Chunlei Cang, Xingbing Wang

**Affiliations:** 1 Department of Hematology, Centre for Leading Medicine and Advanced Technologies of Institute of Health and Medicine, The First Affiliated Hospital of University of Science and Technology of China, Division of Life Sciences and Medicine, University of Science and Technology of China, Hefei, China; 2 State Key Laboratory of Immune Response and Immunotherapy, University of Science and Technology of China, Hefei, China; 3 Department of Rheumatology and Immunology, The First Affiliated Hospital of USTC, Division of Life Sciences and Medicine, University of Science and Technology of China, Hefei, China

**Keywords:** Chronic myeloid leukaemia, Acute myeloid leukaemia

## Abstract

Refractory disease and relapse continue to impede effective treatment of myeloid leukemia, despite substantial progress in therapeutic approaches. Emerging evidence implicates lysosomal ion channels in the regulation of cell death pathways, highlighting these channels as viable targets for therapeutic intervention. This study identified elevated expression of the lysosomal ion channel CLN7 in myeloid leukemia cells. Suppression of CLN7 triggered apoptosis, inhibited cellular proliferation, and markedly reduced the abundance of oncogenic proteins. Mechanistically, CLN7 inhibition promoted nuclear translocation of TFEB by downregulating mTOR signaling, thereby enhancing lysosomal biogenesis and macroautophagy. Notably, CLN7 suppression selectively accelerated chaperone-mediated autophagic degradation of BCR-ABL through cathepsin B (CTSB) upregulation. In addition, inhibition of CLN7 induced autophagy-mediated apoptosis, which led to significant impairment of leukemogenic potential. Co-treatment with chemotherapeutic agents and CLN7 suppression enhanced therapeutic efficacy in myeloid leukemia cells. Finally, suppression of CLN7 markedly reduced tumor growth in human xenograft models without compromising normal hematopoietic function. These findings establish CLN7 as a critical regulator of leukemic cell survival, representing a promising therapeutic target for myeloid leukemia.

## Introduction

Leukemia remains among the frequently diagnosed malignancies and is a leading contributor to cancer-associated mortality worldwide. Myeloid leukemia constitutes the predominant subtype in adults [1, 2] and continues to exhibit high relapse rates and resistance to current therapeutic regimens. Despite significant advances in treatment and mechanistic understanding, outcomes for patients with refractory or relapsed disease remain poor [3, 4], underscoring the urgent need for alternative therapeutic strategies.

Lysosomes have recently emerged as key organelles in cancer pathophysiology [5–7]. Structurally, lysosomes comprise a single lipid bilayer membrane enriched with integral and peripheral membrane proteins, enclosing an acidic lumen filled with hydrolytic enzymes and activators [8]. In malignant cells, lysosomal biogenesis and function are frequently upregulated, contributing to tumor progression through modulation of nutrient sensing, intracellular signaling, programmed cell death, autophagy, immune evasion, and metabolic reprogramming [9–11]. In the context of acute myeloid leukemia (AML), lysosomes also act as reservoirs for chemotherapeutic agents, leading to drug sequestration and therapeutic resistance [12]. During leukemogenesis, AML cells exhibit increased lysosomal mass and heightened enzymatic activity, which are associated with greater membrane fragility compared to non-malignant cells [13, 14].

Maintenance of lysosomal homeostasis depends on the regulation of membrane-embedded proteins, including transporters and ion channels that regulate luminal ion composition, membrane potential, and pH. These membrane proteins have attracted growing attention as therapeutic targets in myeloid leukemia [9]. In particular, pharmacologic inhibition of vacuolar-type ATPase (V-ATPase) disrupts lysosomal acidification, thereby attenuating pH-dependent enzyme activity [15]. Additional lysosome-targeted agents either neutralize luminal acidity to inactivate cathepsin function or induce lysosomal membrane permeabilization to trigger lysosome-dependent cell death (LCD) [16]. Several lysosomal ion channels have been characterized, including two-pore channels (TPCs), transmembrane protein 175 (TMEM175), transient receptor potential mucolipins (TRPMLs), and H^+^/Cl^–^ exchange transporter 7 (CLC-7) [17–19]. However, the therapeutic potential of modulating lysosomal ion channel activity in leukemia remains largely unexplored.

Mutations in CLN7, a lysosomal membrane protein, have been implicated in a variant form of late-infantile neuronal ceroid lipofuscinosis (NCL), a lysosomal storage disorder [20]. More recently, CLN7 has been identified as an organellar chloride channel involved in regulating lysosomal chloride conductance, luminal pH, and membrane potential, and in facilitating lysosomal Ca^2+^ release through TRPML1 [21]. Despite its established roles in lysosomal physiology, the contribution of CLN7 to leukemogenesis has not been defined. Given its broad regulatory influence on lysosomal dynamics, this study investigated whether CLN7 acts as a pathogenic mediator in myeloid leukemia and evaluated its potential as a novel therapeutic target. The findings offer mechanistic insight into CLN7-mediated lysosomal regulation and establish a theoretical basis for developing more effective treatment strategies targeting lysosome-dependent pathways in myeloid leukemia.

## Materials and methods

### Cell lines and culture

OCI-AML3, HL-60 and HEK293T cells were purchased from DSMZ (Braunschweig, Germany), Shanghai Cell Bank of the Chinese Academy of Sciences (Shanghai, China) and ATCC, respectively. THP1 and MV4-11 cells were obtained from Procell (Wuhan, China). K562 and NB4 cells were provided by Anhui Anke Biological Company (China). MV4-11 cells were cultured in Iscove’s Modified Dulbecco’s Medium (IMDM; Procell). HEK293T cells were cultured in Dulbecco’s Modified Eagle’s Medium (DMEM; Gibco). All remaining cells were cultured in RPMI-1640 (Gibco). All basal media were supplemented with 10% fetal bovine serum (FBS; Gibco) and 1% penicillin–streptomycin (Biosharp). All cultures were maintained at 37 °C in a humidified incubator containing 5% CO_2_.

### Human samples

The study was conducted in accordance with the Declaration of Helsinki and under protocols approved by the Ethics Committee of the University of Science and Technology of China (Anhui, China) (Ethics approval:2025-N(H)-325). Peripheral blood samples from four healthy donors and three patients with myeloid leukemia (two AML and one CML) were collected at the First Affiliated Hospital of the University of Science and Technology of China. Peripheral blood mononuclear cells (PBMCs) were isolated using Ficoll density gradient centrifugation with lymphocyte separation medium (Solarbio) according to the manufacturer’s protocols.

### Reagent

Dimethyl sulfoxide (DMSO) was purchased from Sigma-Aldrich (D2650). MG132 was purchased from Selleck (S2619). Bafilomycin A1 (HY-100558), CA-074Me (HY-100350) and MHY1485 (HY-B0795) were sourced from MedChemExpress. Cycloheximide (CHX, T1225) and polybrene (T13720) were purchased from TargetMol. Puromycin was obtained from Aladdin (p113126). Daunorubicin (CSN16731) and cytarabine (CSN19369) were purchased from CSNpharm. Hoechst 33342 was purchased from ImmunoChemistry (639). Lentifit transfection reagent was purchased from HanBio Technology (HB-LLF-1000). Matrigel was obtained from Corning (354234).

### shRNA knockdown

shRNA constructs targeting human *CLN7* (sh*CLN7*#1) were generated by annealing phosphorylated oligonucleotide pairs (forward: 5′-CCGGGCTGGGTTATTGCTTCATATACTCGAGTATATGAAGCAATAACCCAGCTTTTTTG-3′; reverse: 5′-AATTCAAAAAAGCTGGGTTATTGCTTCATATACTCGAGTATATGAAGCAATAACCCAGC-3′) and ligating into AgeI/EcoRI-digested pLKO.1 vectors. Target sequences are listed in Table 1.

**Table 1:** Target sequences for the shRNA.

Name	Target sequences (5’–3’)
sh*CLN7*#1	GCTGGGTTATTGCTTCATATA
sh*CLN7*#2	GCCAAATGGTAGCTTCACCTA
sh*ATG7*	GCTTTGGGATTTGACACATTT
sh*CTSB*	GCTGGTCAACTATGTCAACAA
sh*LAMP2A*	GCCATCAGAATTCCATTGAAT
sh*TRPML1*	CCTGATCACGTTTGACAACAA

Lentiviral particles were generated by co-transfecting HEK293T cells with pLKO.1-based shRNA transfer plasmids, psPAX2 packaging plasmids and pMD2.G envelope plasmids using Lentifit reagent. Supernatants were collected at 36 h and 60 h post-transfection, filtered through 0.45 μm surfactant-free cellulose acetate membranes, and concentrated by ultracentrifugation at 45,000 rpm for 90 min at 4 °C. Virus pellets were resuspended in Hank’s Balanced Salt Solution (HBSS), aliquoted, and stored at −80 °C until further use.

Target cells (K562, OCI-AML3 or NB4) were infected with lentiviral particles in antibiotic-free medium containing polybrene (8 μg/mL). Infected cells were selected with puromycin (1–3 μg/mL) for 48 h. Three days post-infection, cells were harvested for knockdown validation by Western blot or RT-qPCR.

### cDNA constructs and transfections

For lentiviral expression constructs, the plasmid of pSIN-EF1α-LAMP2A(human)-puro was purchased from MiaoLing Bio (Wuhan, China). Lentiviral particles were produced by cotransfection of the lentiviral expression constructs with the lentiviral packing (psPAX2) and envelope (pMD2.G) plasmids into HEK293T cells via lentifit transfection reagent. Viruses were collected and centrifuged, and subsequently were added to target cells in the presence of polybrene (8 μg/mL).

### Real-time quantitative polymerase chain reaction

Total RNA was extracted from K562, OCI-AML3 and NB4 cells using a MolPure® Cell RNA Kit (Yeasen). RNA concentrations were measured using a NanoDrop 2000 spectrophotometer (Thermo Fisher Scientific). First-strand cDNA was synthesized from 1 μg of RNA per sample using HiScript II Q Select RT SuperMix (Vazyme). RT-qPCR was performed on the LightCycler 96 platform (Roche) using SYBR Green Master Mix (Vazyme). Gene expression levels were normalized to GAPDH or ACTB mRNA. Primer sequences are listed in Table 2 (purchased from Generalbiol, Anhui, China).

**Table 2:** Sequences of primers for the RT-qPCR assay.

Name	Forward-primer (5’–3’)	Reverse-primer (5’–3’)
*GAPDH*	GGCCTCCAAGGAGTAAGACC	TGGTACATGACAAGGTGCGG
*CLN7*	CTGCGGAACGAAAGTGAACAG	AGAAAACCCTACACTGCTGAGA
*BCR-ABL*	TCCGCTGACCATCAATAAGGA	CACTCAGACCCTGAGGCTCAA
*TFEB*	CAAGGCCAATGACCTGGAC	AGCTCCCTGGACTTTTGCAG
*CTSB*	CTGGCAGGTTGAAGTAGGGG	CCGCTAATAACGGCAGTTGC
*CTSD*	GACATCCACTATGGCTCGGG	AGCACGTTGTTGACGGAGAT
*ATG7*	GAGCAGCCTTGTGAGAGACA	TTGAGAACGGATGCACTGGA
*LAMP2A*	TGACGACAACTTCCTTGTGC	AGCATGATGGTGCTTGAGAC
*LAMP2B*	TGCTGGTCTTTCAGGCTTGATT	TTGCATGTTGGAACTTGTACTTGC
*p62*	CCGTCTACAGGTGAACTCCAGTCC	AGCCAGCCGCCTTCATCAGAG
*TRPML1*	TTCGCCGTCGTCTCAAATACT	CTCTTCCCGGAATGTCACAGC

### Annexin V/propidium iodide (PI) apoptosis assay

Apoptotic cells were detected using annexin V/PI double staining. K562, OCI-AML3 or NB4 cells were centrifuged at 800 × *g* for 5 min, 4 °C and washed twice with ice-cold phosphate-buffered saline (PBS). Cells were resuspended in annexin-binding buffer and adjusted to a density of 3–5 × 10^5^ cells/100 μL. Annexin V (3 μL) and PI (1 μL) (Thermo, V13245) were added to 100 μL of cell suspension and incubated for 15 min at room temperature. Samples were immediately analyzed by flow cytometry (CytoFlex, Beckman) using the FITC and PI channels.

### CFSE cell proliferation assay

Cell proliferation was assessed by CellTrace CFSE Cell Proliferation Kit (Thermo Fisher Scientific, C34554) staining. Briefly, cells were centrifuged at 800 × *g* for 5 min, 4 °C and resuspended in PBS at a density of 5 × 10^6^ cells/mL, followed by incubation with 5 μM CFSE for 15 min at 37 °C. An equal volume of culture medium (five times the PBS volume) was added, and cells were incubated for an additional 5 min. Cells were then plated in a 12-well plate at 1 × 10^5^ cells/well. After 3 days, cells were harvested, washed twice with ice-cold PBS, and analyzed by flow cytometry using the FITC channel [22].

### Cell viability

Cells were seeded in 96-well plates at 20 000 cells/well and cultured for 24 h at 37 °C in a humidified incubator containing 5% CO_2_. Subsequently, 10 μL of CCK8 reagent (TargetMol, C0005) was added to each well, followed by a 2 h incubation at 37 °C. Absorbance was measured at 450 nm with a microplate reader (Elx800, BioTek) [23].

### Western blotting

Cellular proteins were extracted using NP-40 or SDS lysis buffer supplemented with PMSF (Servicebio), protease and phosphatase inhibitors (Thermo Fisher Scientific, USA, A32961). Lysates were centrifuged at 18,500 × *g* for 10 min at 4 °C, and supernatants were collected. Protein concentrations were determined using a BCA Protein Assay Kit (Beyotime, P0010). Equal amounts of protein were separated using 8–12% sodium dodecyl sulfate-polyacrylamide gel electrophoresis (SDS-PAGE) and transferred to polyvinylidene difluoride (PVDF) membranes (Millipore) using a vertical gel electrophoresis and transfer system (Cavoy Trans-Blot). Membranes were blocked for 2 h at room temperature with 5% nonfat dry milk in Tris-buffered saline with Tween 20 (TBST), then incubated overnight at 4 °C with primary antibodies. After washing with TBST, membranes were incubated with horseradish peroxidase (HRP)-labeled secondary antibodies for 2 h at room temperature. Primary antibodies included: CLN7 (HPA044802; Atlas Antibodies); β-Actin (T0022; Affinity); GAPDH (60004-1-Ig), PARP1 (13371-1-AP), mTOR (668888-1-Ig), phospho-mTOR (Ser2448; 67778-1-Ig), LC3 (14600-1-AP), p62 (66184-1-Ig), LAMP2 (66301-1-AP), HSC70(10654-1-AP), ATP6V1A (17115-1-AP) from Proteintech; Cleaved Caspase-3 (9664), c-ABL (2862), P70S6K (2708), phospho-P70S6K (9234), phospho-S6 (2211) and LAMTOR1 (8975) from Cell Signaling Technology; TRPML1 (ACC-081) from Alomone Labs; LAMP2A (ab125068) from Abcam.

### Flow cytometry analysis of lysosome changes

K562, OCI-AML3 and NB4 scramble and sh*CLN7* cells were collected and washed twice with 1 × PBS by centrifugation at 800 × *g* for 5 min at 4 °C. 5 × 10^5^ cells were resuspended in 1 mL of PBS and incubated with LysoTracker dye (100 nM; Invitrogen) at 37 °C for 30 min in the dark. Cells were subsequently washed with PBS and resuspended in 200 μL of PBS. Fluorescence was detected using a CytoFLEX cytometer (Beckman) in the FITC channel, and data were analyzed with FlowJo.

### Confocal imaging of lysosome morphology, pH and CTSB activity

K562, OCI-AML3 and NB4 scramble and sh*CLN7* cells were resuspended in 1 mL of medium at a density of 1 × 10^6^ cells/mL and incubated with LysoTracker Green (100 nM, Invitrogen) or Magic Red^TM^ Cathepsin B substrate (ImmunoChemistry, ICT-937) at 37 °C for 30 min in the dark. Cells were subsequently centrifuged at 150 × *g* for 4 min and resuspended in 1 mL of fresh medium in 35-mm confocal dishes. The dishes were centrifuged at 400 × *g* for 5 min, 4 °C to position cells at the bottom. For lysosomal pH analysis, 1 × 10^6^ K562 scramble and sh*CLN7* cells were cultured with pHrodo Red Dextran (20 μg/mL, Invitrogen) for 6 h at 37 °C in a humidified incubator containing 5% CO_2_, washed, and then resuspended in 1 mL of fresh medium in 35-mm confocal dishes and incubated at 37 °C for 2 h in the dark before imaging. Fluorescence imaging was performed using a Zeiss-LSM980 high-resolution confocal microscope. Excitation wavelengths were 488 nm for LysoTracker Green and 594 nm for both pHrodo Red Dextran and Magic Red™ Cathepsin B. Fluorescence intensity was analyzed using ImageJ and MATLAB.

### RNA sequencing (RNA-seq)

Scramble and sh*CLN7* K562 and OCI-AML3 cells were collected using TRIzol Reagent (Vazyme). RNA-seq was performed by LC Biotech Corporation (Hangzhou, China). Differentially expressed genes (DEGs) were identified by comparing the experimental and control groups, using a fold-change threshold of ± 1.2 and a q-value cutoff of < 0.05.

### Lysosome isolation

The lysosome isolation was conducted with Minute^TM^ Lysosome Isolation Kit (Invent, USA, LY-034) according to the manufacturer’s instructions.

### Co-immunoprecipitation (Co-IP)

Cells were harvested and lysed in IP lysis buffer (20 mM Tris-HCl, pH 8.0, 137 mM NaCl, 1% Nonidet P-40 and 2 mM EDTA) supplemented with protease inhibitor cocktail (Thermo Fisher Scientific, USA, A32953) and PMSF for 1 h at 4 °C. The supernatants were collected by centrifugation at 12,000 rpm for 20 min at 4 °C and pre-cleared with 50 μl Protein A/G Magnetic Beads (MCE, HY-K0202) at 4 °C for 1 h on a rotator. In all, 10% of the pre-cleared supernatants were saved as input, and the rest were subjected to incubation with the antibodies used for IP: c-ABL (CST, 2862), overnight at 4 °C. The next day, protein A/G Magnetic Beads were added to the reactions for 4 h at 4 °C. The protein-antibody-beads complex was then conducted to IP Wash Buffer (10 mM Tris pH7.4, 1 mM EDTA, 1 mM EGTA pH 8.0, 150 mM NaCl, 1% TritonX-100) containing PMSF and Proteinase Inhibitor Cocktail. Next, the resulting complex was dissolved in the NP-40 lysis buffer and subjected to SDS-PAGE for Western blotting.

### Leukemia xenograft model

All animal experiments were performed under protocols approved by the Laboratory Animal Platform of the National Science Center of Hefei (Ethics approval: IHM-AP-2024-049). Female NOD-SCID mice (5–6 weeks old) were obtained from GemPharmatech (Nanjing, China) and acclimated to the experimental housing facility for one week before tumor implantation. On day 0, hair was removed from the right flank, and mice were randomly assigned to two groups. Scramble or sh*CLN7* OCI-AML3 or NB4 cells (5 × 10^6^) were resuspended in 100 μL of PBS and mixed with 100 μL of Matrigel before subcutaneous injection into the right flank. Tumor volume was monitored using digital calipers and calculated with the formula: (length × width^2^)/2. Mice were euthanized when tumor volumes reached approximately 2000 mm^3^, after which tumors were excised and weighed.

### Quantification and statistical analysis

Data processing and statistical analyses were performed using ImageJ, FlowJo, MATLAB, Excel, and GraphPad Prism 10 (GraphPad Software, USA). All quantitative data were derived from at least three independent biological replicates and are reported as mean ± standard deviation (SD). Comparisons between two groups were analyzed using two-tailed unpaired Student’s *t* tests. Multiple group comparisons were evaluated using one-way analyses of variance (ANOVA) with Bonferroni correction. Data normality and homoscedasticity were assessed using the Shapiro-Wilk test and Bartlett’s test, respectively. Significance thresholds were defined as follows: *p < 0.05, ** *p* < 0.01, *** *p* < 0.001, **** *p* < 0.0001, ns: not significant.

## Results

### CLN7 is highly expressed in myeloid leukemia and modulates lysosomal function

**Fig. 1:**
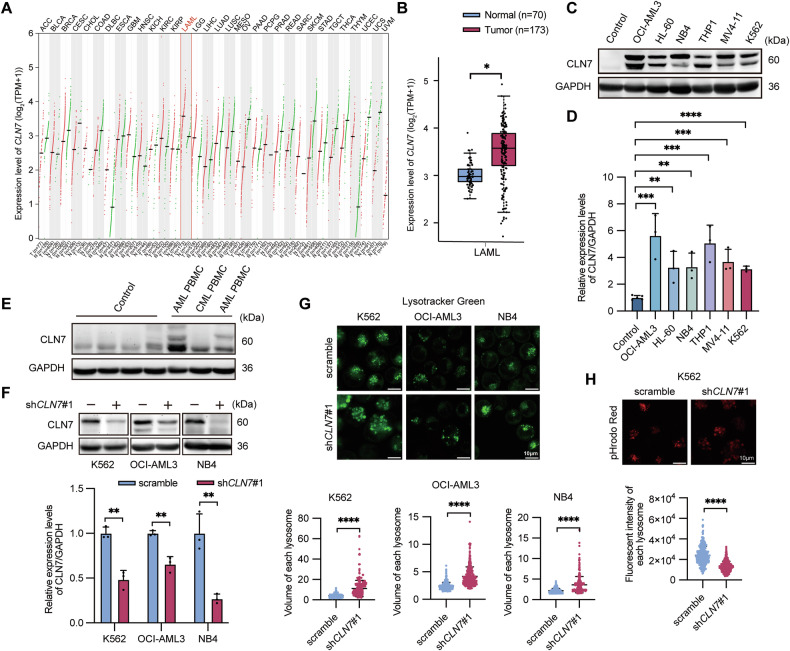
CLN7 is highly expressed in myeloid leukemia and modulates lysosomal function. **A** The RNA levels of *CLN7* analyzed in different cancer tissues and their corresponding normal tissues from TCGA and GTEx database. The statistical graphs are generated using the GEPIA2 website (http://gepia2.cancer-pku.cn) and analyzed using one-way ANOVA. **B** The RNA levels of *CLN7* in AML and their corresponding normal tissues. The box plots show the interquartile range (box limits), median (center line), min and max values with whiskers. **C** CLN7 protein expression levels in myeloid leukemia cell lines were detected by Western blot with healthy PBMCs as control. **D** Quantitative evaluation of protein levels from (**C**) (*n* = 3). **E** CLN7 protein expression levels in healthy PBMCs (Lanes 1–4, *n* = 4) and myeloid leukemia PBMCs (Lanes 5–7, *n* = 3) were detected by Western blot. Lanes 5–7 represent PBMCs from de novo AML, de novo CML, and secondary AML (transformed from MPN) patients, respectively. **F** Western blot analysis of CLN7 protein expression levels in scramble and sh*CLN7*#1 cells of K562, OCI-AML3 and NB4 cells (*n* = 3). **G** Confocal imaging analysis of lysosome changes (Lysotracker Green, 100 nM) of scramble and sh*CLN7*#1 cells in K562 (For scramble cells, *n* = 621 lysosomes. For sh*CLN7*#1 cells, *n* = 656 lysosomes), OCI-AML3 (For scramble cells, *n* = 373 lysosomes. For sh*CLN7*#1 cells, *n* = 323 lysosomes) and NB4 (For scramble cells, *n* = 339 lysosomes. For sh*CLN7*#1 cells, *n* = 370 lysosomes). Scale bars, 10 μm. **H** Confocal imaging analysis of lysosome pH (pHrodo Red Dextran, 20 μg/mL) in K562 scramble (n = 421 lysosomes) and sh*CLN7*#1 (*n* = 453 lysosomes) cells. Scale bars, 10 μm. Data are shown as mean ± SD. *p < 0.05, **p < 0.01, ***p < 0.001, **** *p* < 0.0001.

Analysis of the Cancer Genome Atlas (TCGA) dataset revealed significantly elevated CLN7 expression in AML (Fig. 1A, B). To validate this observation, CLN7 expression was examined in both leukemia cell lines and PBMCs from patients with myeloid leukemia. Compared to PBMCs from healthy donors, CLN7 expression was markedly increased in myeloid leukemia cell lines, including those derived from AML and chronic myeloid leukemia (CML), such as OCI-AML3, HL-60, NB4, THP1, MV4-11 and K562 (Fig. 1C, D). Consistently, PBMCs from myeloid leukemia patients also exhibited elevated CLN7 expression; however, considerable inter-patient variability was observed, likely reflecting individual heterogeneity (Fig. 1E). These results indicate that aberrant expression of the lysosomal ion channel CLN7 may contribute to the pathogenesis of myeloid leukemia.

To investigate the functional relevance of CLN7 in leukemic lysosomes, shRNA was used to silence *CLN7* in K562, OCI-AML3 and NB4 cells. Knockdown efficiency was validated by western blot and RT-qPCR (Figs. 1F and S1A–C). Alterations in lysosomes were observed upon CLN7 suppression (Fig. S1D, E). Further analysis using Lysotracker Green revealed significantly enlarged lysosomes in *CLN7* knockdown cells (Fig. 1G), a phenotype previously linked to impaired lysosomal stability and susceptibility to lysosome-dependent cell death [24, 25]. In addition, pHrodo Red staining demonstrated increased lysosomal pH following *CLN7* knockdown (Fig. 1H), indicating functional compromise. Collectively, these findings suggest that CLN7 regulates lysosomal structure and acidification in myeloid leukemia cells, and its suppression disrupts lysosomal homeostasis, potentially impairing cellular survival.

### CLN7 is essential for the proliferation and survival of leukemia cells

**Fig. 2:**
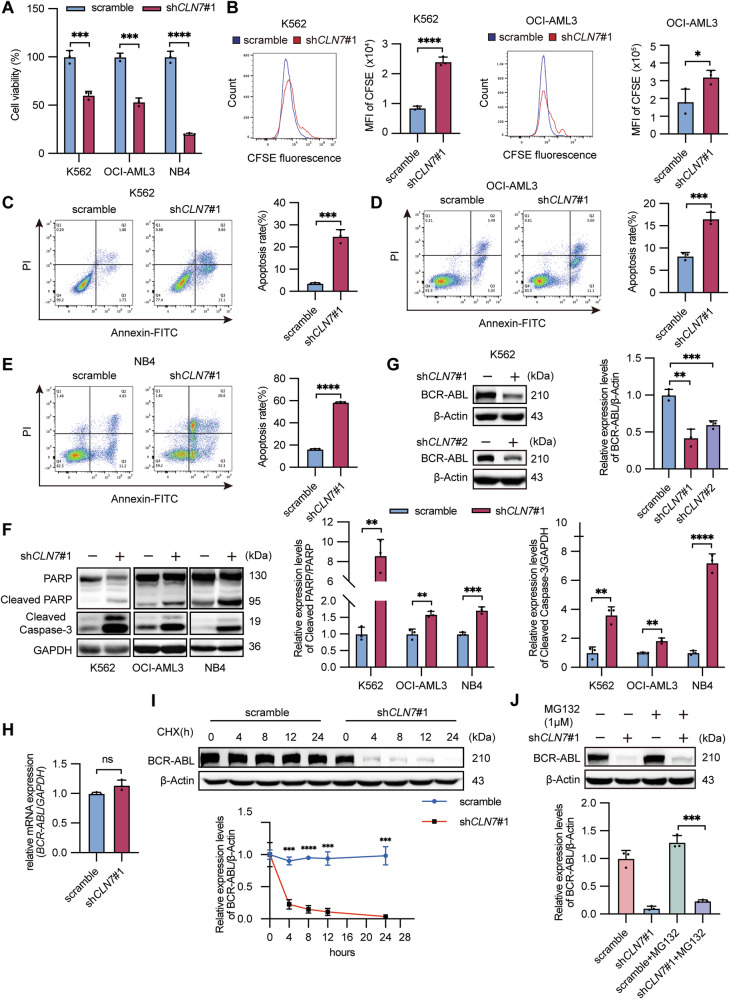
CLN7 is essential for the proliferation and survival of leukemia cells. K562, OCI-AML3 and NB4 cells were transfected with scramble shRNA (scramble) or shRNA against *CLN7* (sh*CLN7*#1 or sh*CLN7*#2). **A** Cell viability was measured using CCK8 in K562, OCI-AML3 and NB4 scramble and sh*CLN7*#1 cells (*n* = 3). **B** Flow cytometry analysis of CFSE staining of scramble and sh*CLN7*#1 cells in K562 and OCI-AML3 (*n* = 3). **C–E** Flow cytometry analysis of apoptosis of scramble and sh*CLN7*#1 cells in K562, OCI-AML3 and NB4 at third day after virus infection (*n* = 3). **F** Western blot analysis of apoptosis-related protein PARP, Cleaved Caspase-3 expression levels of K562, OCI-AML3 and NB4 scramble and sh*CLN7*#1 cells (*n* = 3). **G** Western blot analysis of BCR-ABL protein expression levels of scramble and sh*CLN7*#1 or sh*CLN7*#2 cells in K562 (*n* = 3). **H** RT-qPCR analysis of the *BCR-ABL* mRNA expression levels of K562 scramble and sh*CLN7*#1 cells (*n* = 3). **I** Western blot analysis of BCR-ABL protein expression levels of K562 scramble and sh*CLN7*#1 cells treated with different time of CHX (50 μg/mL) (*n* = 3). **J** Western blot analysis of BCR-ABL protein expression levels of K562 scramble and sh*CLN7*#1 cells treated with or without MG132 (1 μM) for 24 h (*n* = 3). Data are shown as mean ± SD. *p < 0.05, **p < 0.01, ***p < 0.001, **** *p* < 0.0001, ns, not significant. CHX: cycloheximide.

To investigate the functional importance of CLN7 in myeloid leukemia, a series of phenotypic assays was conducted. Results showed that partial depletion of CLN7 significantly reduced cell viability across multiple leukemia cell lines (Figs. 2A and S2A). Cell proliferation was also markedly suppressed upon CLN7 depletion, as assessed by CFSE-based analysis (Figs. 2B and S2B, C). To elucidate the mechanism underlying this proliferative defect, annexin V/PI double staining was performed. Notably, *CLN7* knockdown markedly increased annexin V positivity, indicating enhanced apoptotic cell death (Figs. 2C–E and S2D). Consistently, levels of apoptosis-related proteins—including PARP and Cleaved Caspase-3—were elevated following CLN7 depletion (Figs. 2F and S2E). Collectively, these findings demonstrate that CLN7 supports the viability and proliferative capacity of myeloid leukemia cells and that its loss compromises cellular expansion in vitro.

Notably, the pro-apoptotic effect of *CLN7* knockdown was more pronounced in K562 cells than in OCI-AML3 cells, particularly at later time points following CLN7 depletion (Fig. S2F). Further investigation revealed that suppression of CLN7 downregulated the expression levels of leukemia-associated oncoproteins, including BCR-ABL, in K562 cells (Fig. 2G).

Given that protein turnover is governed by transcription, translation, and degradation processes [26], the impact of *CLN7* silencing on *BCR-ABL* expression was examined. Initially, RT-qPCR revealed no significant change in *BCR-ABL* mRNA expression levels (Fig. 2H), indicating post-transcriptional regulation. Subsequently, cycloheximide (CHX) chase assays demonstrated accelerated degradation of BCR-ABL protein in CLN7-depleted cells (Fig. 2I). To establish whether BCR-ABL degradation induced by CLN7 suppression is proteasome- or lysosome-dependent, K562 cells were treated with the proteasome inhibitor MG132 or lysosomal inhibitor Baf A1. While BCR-ABL degradation persisted despite MG132 treatment (Fig. 2J), the downregulation of BCR-ABL protein levels triggered by *CLN7* knockdown was partially reversed upon Baf A1 treatment (Fig. S2G). These findings indicate that the lysosomal pathway, rather than the ubiquitin-proteasome system, mediates its turnover.

### Inhibition of CLN7 enhances lysosomal activity and induces BCR-ABL degradation

**Fig. 3:**
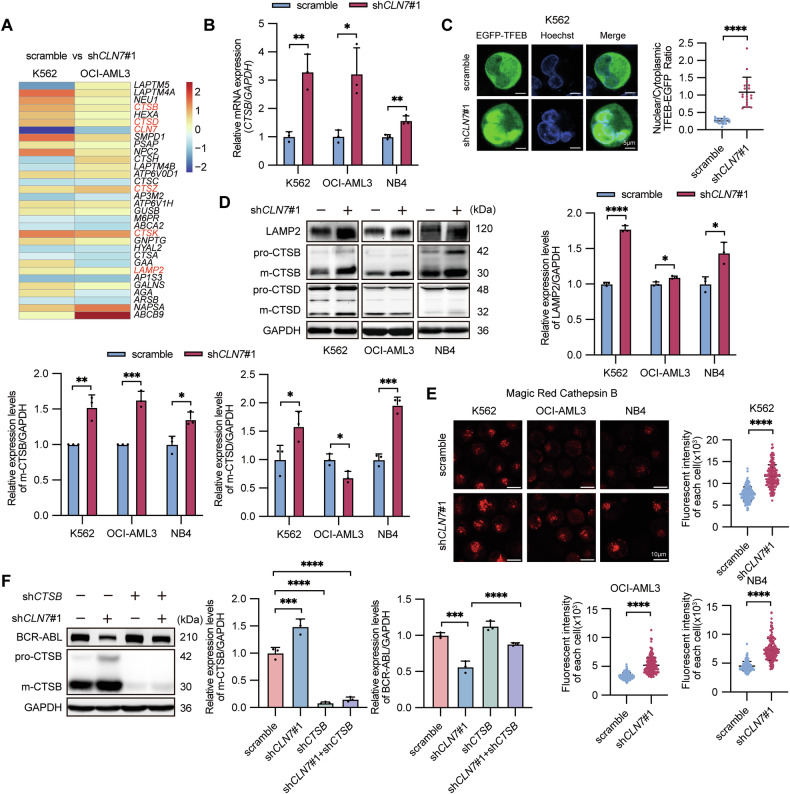
Inhibition of CLN7 enhances lysosomal activity and induces BCR-ABL degradation. **A** Heatmap showing the differential gene expression which are related to lysosome between sh*CLN7*#1 and scramble groups of K562 and OCI-AML3. **B** RT-qPCR analysis of the *CTSB* mRNA expression levels of scramble and sh*CLN7*#1 in K562, OCI-AML3 and NB4 cells (*n* = 3). **C** Confocal imaging analysis of EGFP-TFEB nuclear translocation in scramble (*n* = 25) and sh*CLN7*#1 (*n* = 18) cells of K562. Scale bars, 5 μm. **D** Western blot analysis of lysosome-related proteins LAMP2, CTSB CTSD expression levels in K562, OCI-AML3 and NB4 scramble and sh*CLN7*#1 cells (*n* = 3). **E** Confocal imaging analysis of lysosome CTSB activity of scramble and sh*CLN7*#1 cells in K562 (For scramble cells, *n* = 168 cells. For sh*CLN7*#1 cells, *n* = 145 cells), OCI-AML3 (For scramble cells, *n* = 188 cells. For sh*CLN7*#1 cells, *n* = 185 cells) and NB4 (For scramble cells, *n* = 161 cells. For sh*CLN7*#1 cells, *n* = 172 cells). Scale bars, 10 μm. **F** Western blot analysis of BCR-ABL and CTSB protein expression levels of scramble, *CLN7* knockdown, *CTSB* knockdown, *CLN7* and *CTSB* combined knockdown cells of K562 (*n* = 3). Data are shown as mean ± SD. *p < 0.05, **p < 0.01, ***p < 0.001, **** *p* < 0.0001.

To determine whether BCR-ABL is degraded through lysosomal pathways following CLN7 depletion, RNA-seq analysis was performed on scramble and *CLN7* knockdown K562 and OCI-AML3 cells. A total of 3455 overlapping DEGs were identified between both cell lines (Fig. S3A, B). Inhibition of CLN7 significantly altered the expression of lysosome-related genes, including those encoding lysosomal membrane proteins and proteolytic enzymes (Fig. 3A).

The mRNA expression levels of *CTSB* and *CTSD* were significantly upregulated upon CLN7 depletion, indicating upregulated lysosomal biogenesis (Figs. 3B and S3C). Although total expression of *TFEB*, a master regulator of lysosomal biogenesis [27], was not upregulated (Fig. S3D), increased nuclear translocation of TFEB was observed (Figs. 3C and S3E), consistent with transcriptional activation of lysosomal genes. Western blot confirmed increased protein levels of LAMP2, CTSB and CTSD in *CLN7* knockdown cells, with the exception of CTSD in OCI-AML3 cells, indicating an overall enhancement in lysosomal degradation capacity (Fig. 3D). Confocal imaging further verified elevated CTSB activity following *CLN7* knockdown (Fig. 3E).

Given the known role of CTSB in lysosome-mediated proteolysis, this protease was examined as a candidate mediator of BCR-ABL degradation [28]. Interestingly, the reduction of BCR-ABL protein levels induced by CLN7 deficiency was partially rescued by either simultaneous knockdown of *CTSB* (Fig. 3F) or pharmacological inhibition of CTSB using CA-074Me (Fig. S3F), further implicating CTSB as a key mediator in the degradation process. To confirm whether BCR-ABL is directly degraded within the lysosomal compartment, we performed organelle fractionation to isolate pure lysosomal fractions from K562 cells. These isolated lysosomes showed a marked decrease in BCR-ABL protein levels following *CLN7* knockdown. Remarkably, the depletion of CTSB effectively stabilized BCR-ABL within the lysosomes, restoring its levels to match the scramble (Fig. S3G). These results demonstrate that CLN7 inhibition enhances lysosomal biogenesis and protease activity—particularly CTSB—thereby promoting degradation of BCR‑ABL in myeloid leukemia cells.

### CLN7 suppression activates autophagy and accelerates chaperone-mediated autophagic degradation of BCR-ABL

**Fig. 4:**
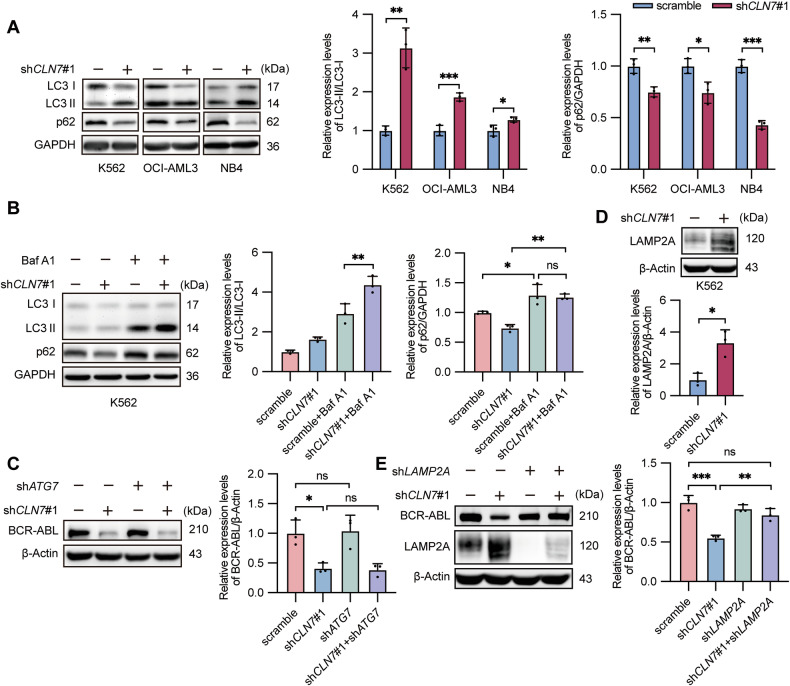
CLN7 suppression activates autophagy and accelerates chaperone-mediated autophagic degradation of BCR-ABL. **A** Western blot analysis of LC3 and p62 protein expression levels in K562, OCI-AML3 and NB4 scramble and sh*CLN7*#1 cells (*n* = 3). **B** Western blot analysis of LC3 (2 h) and p62 (12 h) protein expression levels in K562 scramble and sh*CLN7*#1 cells treated with Baf A1(100 nM) (*n* = 3). **C** Western blot analysis of BCR-ABL protein expression levels of scramble, *CLN7* knockdown, *ATG7* knockdown, *CLN7* and *ATG7* combined knockdown cells of K562 (*n* = 3). **D** Western blot analysis of LAMP2A protein expression levels of K562 scramble and sh*CLN7*#1 cells (*n* = 3). **E** Western blot analysis of BCR-ABL and LAMP2A protein expression levels of scramble, *CLN7* knockdown, *LAMP2A* knockdown, *CLN7* and *LAMP2A* combined knockdown cells of K562 (*n* = 3). Data are shown as mean ± SD. *p < 0.05, **p < 0.01, ***p < 0.001, ns, not significant.

BCR-ABL is sequestered into lysosomes via the autophagy pathway for subsequent degradation. Autophagic degradation occurs via distinct pathways, including macroautophagy, microautophagy, and chaperone-mediated autophagy (CMA) [29]. To evaluate the impact of CLN7 depletion on autophagic processes, macroautophagy-related markers were first assessed. Notably, *CLN7* knockdown resulted in accumulation of LC3 and a concomitant reduction in p62 protein levels (Fig. 4A), consistent with enhanced autophagosome maturation and lysosomal degradation. Transcriptional upregulation of *ATG7* and *p62* (Fig. S4A), together with increased conversion of LC3-I to LC3-II in the presence of Baf A1 (Fig. 4B), further confirmed the accumulation of autophagosomes is caused by increased synthesis. These data collectively indicate that CLN7-deficient cells exhibit elevated macroautophagy.

To determine whether this process contributes to BCR‑ABL turnover, *ATG7* was silenced to block autophagosome formation. However, inhibition of macroautophagy failed to restore BCR‑ABL protein expression (Fig. 4C), suggesting that BCR‑ABL is not primarily degraded through this pathway in the context of CLN7 suppression.

CMA is a highly selective lysosomal pathway that recognizes specific proteins containing a pentapeptide KFERQ-like motif, targeting them for HSC70-mediated delivery to LAMP2A at the lysosomal membrane [30]. To determine whether BCR-ABL degradation upon CLN7 suppression occurs via the CMA pathway, expression of LAMP2A was examined and found to be significantly elevated following *CLN7* knockdown (Figs. 4D and S4B). Using the KFERQ finder V0.8, we identified five potential CMA-targeting motifs within the BCR-ABL sequence (four in the BCR domain and one in ABL1). Given the large size of the fusion protein, we performed molecular docking between the BCR domain (1–927aa) and HSC70 using the HADDOCK 2.4 server. The top-ranked docking model revealed a stable interaction interface, specifically involving the RRLEQ motif (residues 43–47), which fits precisely into the substrate-binding pocket of HSC70 (Fig. S4C). To corroborate the modeling data, we performed Co-IP assays in K562 cells. Consistent with our structural predictions, *CLN7* knockdown significantly increased the recruitment of HSC70 to BCR-ABL (Fig. S4D). Dual suppression of CLN7 and LAMP2A partially reversed BCR-ABL loss (Figs. 4E and S4E), implicating in the absence of CLN7, BCR-ABL is more efficiently captured toward the CMA machinery for lysosomal delivery.

Together, these findings provide compelling evidence that although CLN7 inhibition induces broad autophagic activation, BCR‑ABL degradation is specifically mediated via the chaperone-dependent lysosomal pathway rather than canonical macroautophagy.

### mTOR-mediated autophagy activation drives apoptosis following CLN7 suppression

**Fig. 5:**
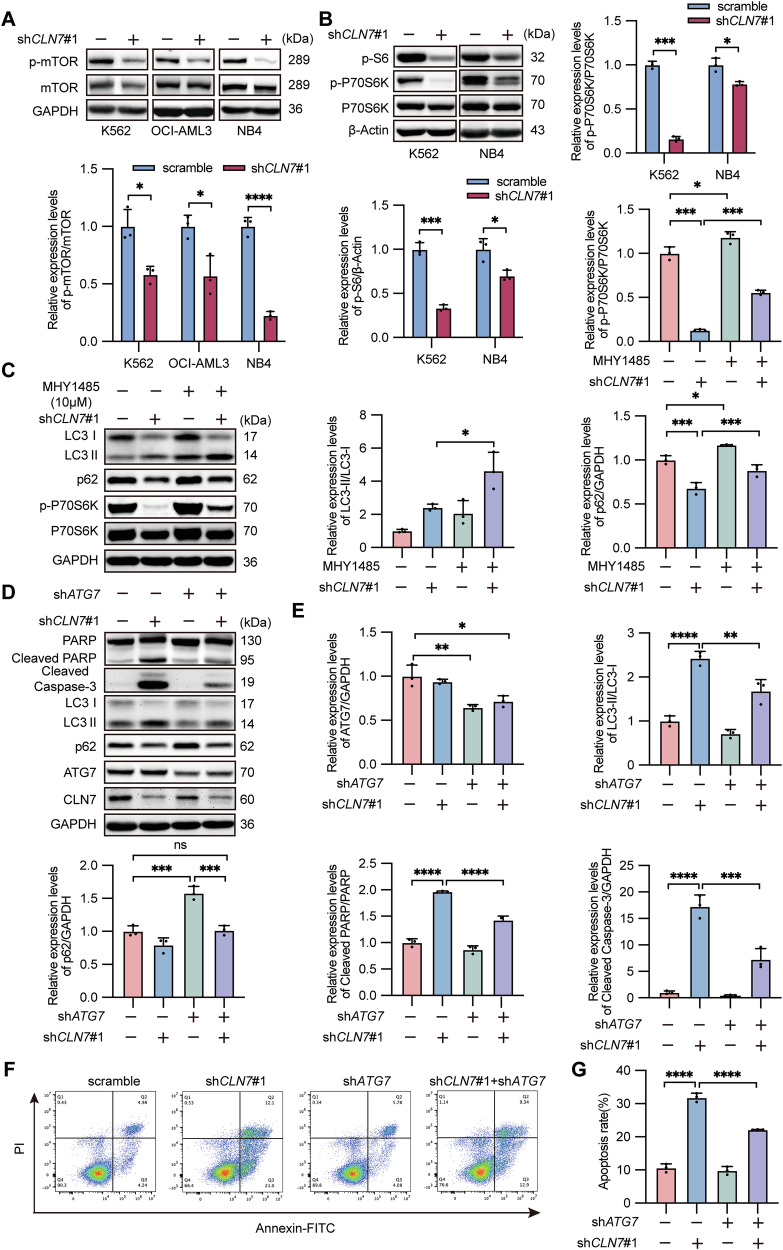
mTOR-mediated autophagy activation drives apoptosis following CLN7 suppression. **A** Western blot analysis of mTOR and p-mTOR protein expression levels in K562, OCI-AML3 and NB4 scramble and sh*CLN7*#1 cells (*n* = 3). **B** Western blot analysis of p-S6, P70S6K and p-P70S6K protein expression levels in K562 and NB4 scramble and sh*CLN7*#1 cells (*n* = 3). **C** Western blot analysis of LC3, p62, P70S6K and p-P70S6K protein expression levels of K562 scramble and sh*CLN7*#1 cells treated with or without MHY1485 (mTOR agonist,10 μM) for 24 h (*n* = 3). **D** Western blot analysis of LC3, p62, PARP, Cleaved Caspase-3, ATG7 and CLN7 protein expression levels of scramble, *CLN7* knockdown, *ATG7* knockdown, *CLN7* and *ATG7* combined knockdown cells of K562. **E** Statistics of relative protein expression levels in (**D**) (*n* = 3). **F** Flow cytometry analysis of apoptosis of scramble, *CLN7* knockdown, *ATG7* knockdown, *CLN7* and *ATG7* combined knockdown cells of K562. **G** Statistics of the apoptosis rates in (**F**) (*n* = 3). Data are shown as mean ± SD. *p < 0.05, **p < 0.01, ***p < 0.001, **** *p* < 0.0001, ns, not significant.

mTOR functions as a central signaling kinase that integrates growth and stress cues to coordinate macroautophagy and lysosomal biogenesis, largely through regulation of TFEB activity [31–33]. Accumulating evidence also links lysosomal ion channel activity to modulation of mTOR signaling [17, 34, 35]. Consistent with this regulatory framework, suppression of CLN7 resulted in sustained inhibition of mTORC1 signaling, as indicated by reduced phosphorylation of downstream targets (Figs. 5A, B and S5A). Western blot analysis of amino acid starvation/repletion assays and isolated lysosomes revealed that *CLN7* knockdown did not alter its responsiveness to nutrient signaling (Fig. S5B) or mTOR lysosomal localization (Fig. S5C). These results suggest that CLN7 deficiency inhibits mTORC1 through a non-canonical mechanism independent of the classical Rag-dependent recruitment and amino acid sensing. Pharmacological activation of mTOR using MHY1485 attenuated autophagic responses and restored LC3 and p62 protein levels (Fig. 5C), indicating that autophagy induction following CLN7 suppression is mediated via mTOR signaling.

To further delineate the functional relationship between autophagy and apoptosis [36], autophagosome formation was disrupted through *ATG7* knockdown. Inhibition of autophagic initiation restored LC3 and p62 protein levels and markedly reduced accumulation of apoptosis-associated markers, including PARP and cleaved caspase-3 (Fig. 5D, E). Consistently, annexin V staining revealed a significant reduction in apoptotic cell populations following combined suppression of CLN7 and ATG7 (Fig. 5F, G). Collectively, these results demonstrate that CLN7 suppression induces mTOR-dependent autophagy, which acts as a pro-death mechanism and contributes directly to apoptosis in myeloid leukemia cells.

### CLN7 suppression enhances the cytotoxic effects of chemotherapeutic agents in myeloid leukemia cells

**Fig. 6:**
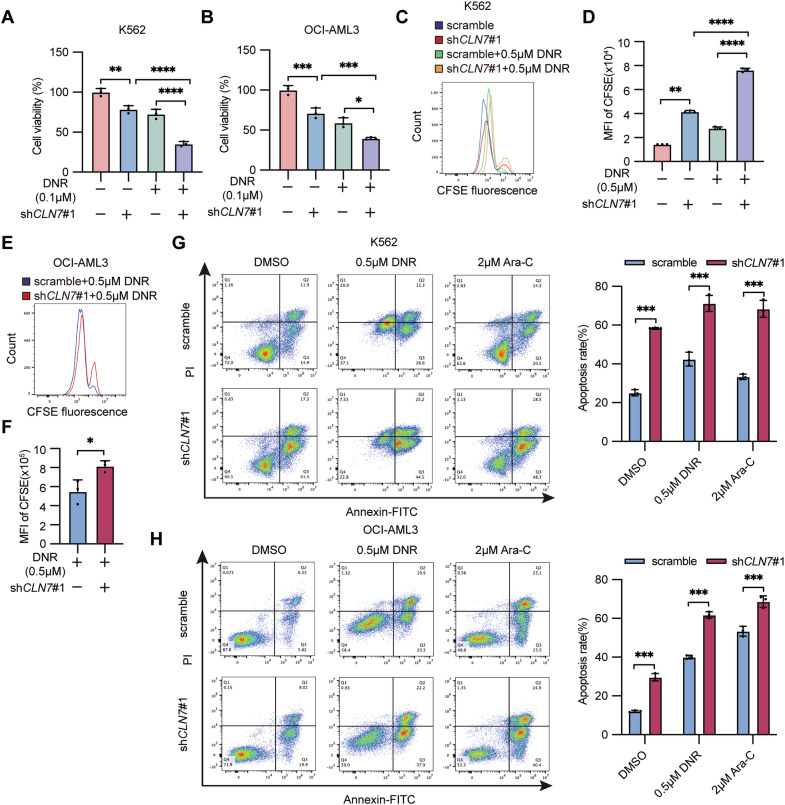
CLN7 suppression enhances the cytotoxic effects of chemotherapeutic agents in myeloid leukemia cells. **A, B** Cell viability was measured using CCK8 in K562 and OCI-AML3 scramble and sh*CLN7*#1 cells treated with or without DNR (0.1 μM) for 24 h (*n* = 3). **C** Flow cytometry analysis of CFSE staining in K562 scramble and sh*CLN7*#1 cells treated with or without DNR (0.5 μM) for 72 h. **D** Statistics of mean fluorescence intensity of CFSE in (**C**) (*n* = 3). **E** Flow cytometry analysis of CFSE staining in OCI-AML3 scramble and sh*CLN7*#1 cells treated with DNR (0.5 μM) for 72 h. **F** Statistics of mean fluorescence intensity of CFSE in (**E**) (*n* = 3). **G, H** Apoptosis detection of scramble and sh*CLN7*#1 cells of K562 and OCI-AML3 treated with or without DNR (0.5 μM) or Ara-C (2 μM) for 48 h (*n* = 3). Data are shown as mean ± SD. *p < 0.05, **p < 0.01, ***p < 0.001, **** *p* < 0.0001. DNR: Daunorubicin; Ara-C: cytarabine.

To evaluate whether reduced CLN7 expression alters sensitivity to chemotherapy, viability assays were performed in K562 and OCI-AML3 cells following treatment with daunorubicin (DNR). *CLN7* knockdown significantly potentiated the cytotoxic effects of DNR, resulting in further reductions in cell viability compared to control groups (Fig. 6A, B). CFSE-based proliferation assays confirmed that DNR treatment markedly suppressed proliferation in both cell lines, and this effect was amplified in CLN7-depleted cells (Fig. 6C–F). In addition, CLN7 suppression significantly increased apoptosis following exposure to either DNR or cytarabine (Ara-C), as evidenced by annexin V staining (Fig. 6G, H) and Western blot (Fig. S6). Collectively, these findings indicate that silencing *CLN7* sensitizes myeloid leukemia cells to chemotherapeutic agents and enhances the efficacy of standard frontline treatments.

### Inhibition of CLN7 suppresses tumor growth in vivo without disrupting hematopoietic homeostasis

**Fig. 7:**
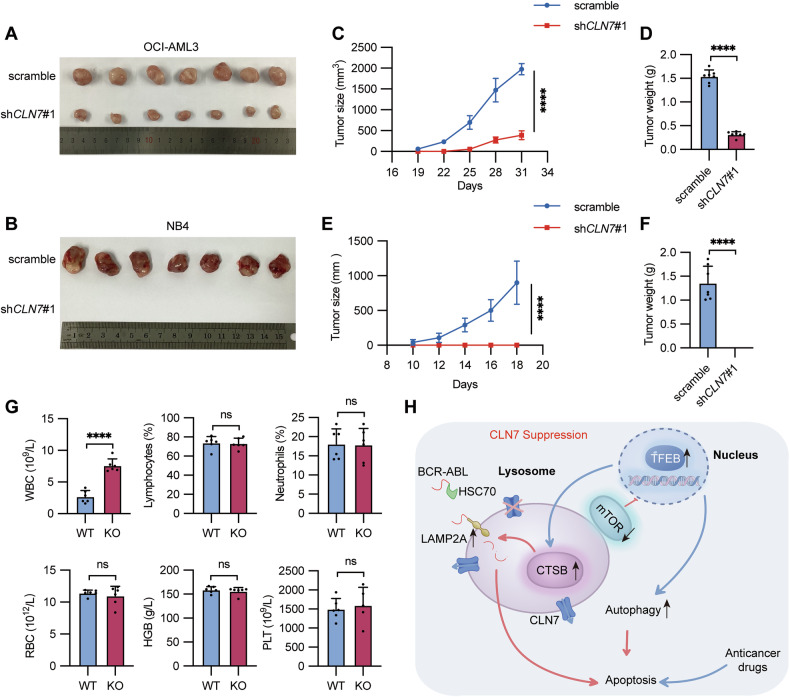
Inhibition of CLN7 suppresses tumor growth in vivo without disrupting hematopoietic homeostasis. **A, B** Macroscopic appearance of isolated tumors of scramble (*n* = 7) and *CLN7* knockdown (sh*CLN7*#1) (*n* = 7) in OCI-AML3 and NB4 cells after mice were euthanatized. **C, D** Tumor growth curves and tumor weight of two groups in (**A**). **E, F** Tumor growth curves and tumor weight of two groups of (**B**). **G** Concentration of WBC, RBC, HGB, PLT and percentage of Lymphocytes and Neutrophils in 8–10-month WT (*n* = 6) or CLN7-KO (*n* = 6) C57BL/6 N mice blood. **H** Working model: inhibition of CLN7 induces apoptosis by promoting TFEB nuclear translocation via mTOR downregulation, which enhances lysosomal activity and macroautophagy levels. Additionally, suppression of CLN7 accelerates CMA-mediated BCR-ABL degradation through upregulated cathepsin B. Combining CLN7 suppression with chemotherapy enhances therapeutic efficacy in myeloid leukemia cells. Data are shown as mean ± SD. **** *p* < 0.0001, ns, not significant.

To assess the in vivo relevance of CLN7 suppression, OCI-AML3 and NB4 cells transduced with either non-targeting (scramble) or *CLN7*-targeting shRNA were subcutaneously implanted into the axillary regions of NOD-SCID mice to establish AML xenografts (Fig. S7A, B). Upon sacrifice, tumors were excised and analyzed (Fig. 7A, B). Mice bearing CLN7-depleted cells exhibited markedly reduced tumor burden, as reflected by significant decreases in both tumor volume and weight in the OCI-AML3 (Fig. 7C, D) and NB4 models (Fig. 7E, F).

To evaluate the systemic safety of CLN7 inhibition, hematological profiles were examined in CLN7 knockout and wild-type mice. Routine blood tests revealed no major alterations across most hematological parameters. Although white blood cell counts were slightly elevated in CLN7-deficient mice, values remained within normal physiological limits (Fig. 7G). These findings demonstrate that targeting CLN7 effectively impairs leukemia progression in vivo without inducing hematopoietic toxicity, supporting its therapeutic potential in myeloid leukemia.

## Discussion

This study identified CLN7 as a critical regulator of lysosomal homeostasis and survival in myeloid leukemia. Suppression of CLN7 enhanced lysosomal biogenesis and macroautophagy through mTOR downregulation, leading to increased nuclear translocation of TFEB. This lysosomal reprogramming was accompanied by the induction of apoptosis and impaired proliferation, driven in part by cathepsin B-dependent degradation of the oncoprotein BCR‑ABL via chaperone-mediated autophagy in K562 cells.

Recent findings have highlighted lysosomal ion channels as promising therapeutic targets in hematologic malignancies. For instance, targeting TPC2 has been shown to sensitize acute lymphoblastic leukemia cells to chemotherapeutics by disrupting lysosomal function, while TRPML1 blockade sensitizes AML cells through autophagy suppression [24, 37, 38]. However, comparing the effects of *CLN7* knockdown with the inhibition of TRPML1 in K562 cells, our data shows that while targeting TRPML1 failed to induce significant apoptosis (Fig. S8A–C). Although most studies have focused on how regulating lysosomal ion channels can alleviate chemotherapy resistance, the present findings extend this concept by demonstrating that CLN7 suppression not only enhances the cytotoxicity of frontline chemotherapeutic agents but also directly promotes apoptotic elimination of leukemia cells through lysosome-mediated degradation of oncogenic drivers, such as BCR-ABL.

Notably, this study provides the first evidence that CLN7 suppression activates CMA through upregulation of LAMP2A, resulting in the degradation of BCR-ABL. While previous studies have established that immature BCR-ABL is directed to proteasomal degradation via Bag1, and mature BCR-ABL undergoes Cbl-mediated ubiquitination and lysosomal degradation [39, 40], the present findings uncover an alternative degradation route. Notably, forced overexpression of LAMP2A in K562 cells did not alter BCR-ABL levels (Fig. S8D), suggesting that CLN7 suppression may trigger conformational changes in BCR-ABL that expose a previously inaccessible KFERQ-like motif, promoting self-degradation. This hypothesis provides a mechanistic link between lysosomal function and BCR-ABL degradation, offering a new perspective on the regulation of this critical signaling molecule. Recent efforts to degrade BCR-ABL have focused on proteolysis-targeting chimera (PROTAC) technologies [41–43]. In contrast, the current findings revealed that endogenous lysosomal pathways can be leveraged to reduce BCR‑ABL abundance upon CLN7 suppression. Although BCR‑ABL degradation via CMA was confirmed, the molecular link between CLN7 suppression and this selective turnover remains undefined. Determining how lysosomal changes trigger substrate recognition and degradation may uncover new strategies for eliminating oncogenic proteins through endogenous autophagy pathways.

Emerging evidence also suggests that lysosomal ion channels exert regulatory control over mTOR signaling [34, 35]. Our study reveals that CLN7 deficiency inhibits mTORC1 through a non-canonical mechanism, as it affects neither mTORC1 lysosomal recruitment nor its nutrient-sensing capability. This suggests that CLN7, as a chloride channel, likely regulates mTORC1 by modulating lysosomal homeostasis, rather than through direct nutrient-dependent signaling. Suppression of CLN7 markedly reduced mTORC1 activity, a change that facilitated nuclear translocation of TFEB and promoted lysosomal biogenesis and macroautophagy [32, 44, 45]. CLN7 suppression also triggered caspase-dependent apoptosis, which was significantly attenuated by *ATG7* knockdown, implicating autophagy as a necessary effector of cell death under CLN7-deficient conditions [46]. The accumulation of autophagosomes likely enabled the formation of intracellular death-inducing signaling complexes that recruited and activated caspase-8, thereby initiating the caspase-8–caspase-3–PARP cascade [36, 47].

Lysosomes play a crucial role in chemotherapy resistance by modulating drug sequestration, metabolic signaling, and gene regulation. Their ability to accumulate weakly basic compounds depends critically on lysosomal integrity and acidity [24]. In the present study, CLN7 suppression enlarged lysosomes and elevated luminal pH, suggesting a compromised sequestration capacity. This lysosomal dysfunction may underlie the enhanced therapeutic efficacy of chemotherapeutic agents when combined with CLN7 inhibition in myeloid leukemia cells.

The observed elevation of CTSB activity in leukemia cells contrasts with the impaired lysosomal degradation typically reported in CLN7-deficient neurons. This suggests a cell-type-specific compensatory mechanism; while neurons are highly susceptible to CLN7 loss, certain malignant or highly metabolic cells (like myeloid leukemia cells and HEK293T) may upregulate alternative lysosomal proteases to maintain proteostasis, a plasticity not observed in HeLa cells or primary neurons (Fig. S8E, F).

While these results support CLN7 as a promising target for the treatment of myeloid leukemia, several limitations remain. First, although the efficacy observed in subcutaneous xenografts, these models lack the physiological bone marrow microenvironment. Subsequent research employing orthotopic engraftment will be essential to validate the role of CLN7 in systemic leukemia and overall survival. Regarding safety, favorable hematopoietic safety was observed; nonetheless, chronic systemic inhibition poses a risk of on-target central nervous system toxicity, as CLN7 loss causes neuronal ceroid lipofuscinosis. Establishing a safe therapeutic window for leukemia will require utilizing transient inhibition regimens or developing brain-sparing agents. Finally, despite the genetic suppression of CLN7 yielding antitumor effects, the absence of structural information precludes the development of selective inhibitors for pharmacologic validation. Therefore, future efforts will focus on identifying CLN7-directed compounds to assess their translational potential in preclinical models of myeloid leukemia.

Summed up, this study demonstrated that CLN7 suppression triggered apoptosis by enhancing lysosomal biogenesis and macroautophagy through TFEB nuclear translocation driven by mTOR inhibition. In K562 cells, CLN7 depletion further promoted chaperone-mediated autophagic degradation of BCR-ABL, facilitated through cathepsin B upregulation. Collectively, these findings identify CLN7 as a viable therapeutic target in myeloid leukemia.

## Supplementary information

SI-CLN7 suppression induces apoptosis via mTOR-regulated and chaperone-mediated autophagy in myeloid leukemia cells

Original Western Blot

## Data Availability

All data generated or analyzed during this study are included in this published article, its supplementary information files and publicly available repositories. The RNA-seq data from K562 and OCI-AML3 cells are accessible at Gene Expression Omnibus (accession number: GSE325745).

## References

[1] Sung H, Ferlay J, Siegel RL, Laversanne M, Soerjomataram I, Jemal A, et al. Global Cancer Statistics 2020: GLOBOCAN Estimates of Incidence and Mortality Worldwide for 36 Cancers in 185 Countries. CA Cancer J Clin. 2021;71:209–49. 33538338 10.3322/caac.21660

[2] Siegel RL, Giaquinto AN, Jemal A. Cancer statistics, 2024. CA Cancer J Clin. 2024;74:12–49. 38230766 10.3322/caac.21820

[3] Dohner H, Wei AH, Appelbaum FR, Craddock C, DiNardo CD, Dombret H, et al. Diagnosis and management of AML in adults: 2022 recommendations from an international expert panel on behalf of the ELN. Blood. 2022;140:1345–77. 35797463 10.1182/blood.2022016867

[4] Thol F, Dohner H, Ganser A. How I treat refractory and relapsed acute myeloid leukemia. Blood. 2024;143:11–20. 37944143 10.1182/blood.2023022481

[5] Ballabio A, Bonifacino JS. Lysosomes as dynamic regulators of cell and organismal homeostasis. Nat Rev Mol Cell Biol. 2020;21:101–18. 31768005 10.1038/s41580-019-0185-4

[6] Chauhan N, Patro BS. Emerging roles of lysosome homeostasis (repair, lysophagy and biogenesis) in cancer progression and therapy. Cancer Lett. 2024;584:216599. 38135207 10.1016/j.canlet.2023.216599

[7] Qi J, Xing Y, Liu Y, Wang MM, Wei X, Sui Z, et al. MCOLN1/TRPML1 finely controls oncogenic autophagy in cancer by mediating zinc influx. Autophagy. 2021;17:4401–22. 33890549 10.1080/15548627.2021.1917132 PMC8726724

[8] Settembre C, Fraldi A, Medina DL, Ballabio A. Signals from the lysosome: a control centre for cellular clearance and energy metabolism. Nat Rev Mol Cell Biol. 2013;14:283–96. 23609508 10.1038/nrm3565 PMC4387238

[9] Rafiq S, McKenna SL, Muller S, Tschan MP, Humbert M. Lysosomes in acute myeloid leukemia: potential therapeutic targets? Leukemia. 2021;35:2759–70. 34462526 10.1038/s41375-021-01388-x PMC8478647

[10] Zhang Z, Yue P, Lu T, Wang Y, Wei Y, Wei X. Role of lysosomes in physiological activities, diseases, and therapy. J Hematol Oncol. 2021;14:79. 33990205 10.1186/s13045-021-01087-1 PMC8120021

[11] Ma J, Ma R, Zeng X, Zhang L, Liu J, Zhang W, et al. Lysosome blockade induces divergent metabolic programs in macrophages and tumours for cancer immunotherapy. J Exp Clin Cancer Res. 2023;42:192. 37537587 10.1186/s13046-023-02768-0 PMC10401909

[12] Zhitomirsky B, Assaraf YG. Lysosomes as mediators of drug resistance in cancer. Drug Resist Updat. 2016;24:23–33. 26830313 10.1016/j.drup.2015.11.004

[13] Sukhai MA, Prabha S, Hurren R, Rutledge AC, Lee AY, Sriskanthadevan S, et al. Lysosomal disruption preferentially targets acute myeloid leukemia cells and progenitors. J Clin Investig. 2013;123:315–28. 23202731 10.1172/JCI64180 PMC3533286

[14] Zeng Y, Zhang X, Lin D, Feng X, Liu Y, Fang Z, et al. A lysosome-targeted dextran-doxorubicin nanodrug overcomes doxorubicin-induced chemoresistance of myeloid leukemia. J Hematol Oncol. 2021;14:189. 34749790 10.1186/s13045-021-01199-8 PMC8576957

[15] Stransky L, Cotter K, Forgac M. The function of V-ATPases in cancer. Physiol Rev. 2016;96:1071–91. 27335445 10.1152/physrev.00035.2015 PMC4982037

[16] Bonam SR, Wang F, Muller S. Lysosomes as a therapeutic target. Nat Rev Drug Discov. 2019;18:923–48. 31477883 10.1038/s41573-019-0036-1 PMC7097195

[17] Riederer E, Cang C, Ren D. Lysosomal ion channels: what are they good for and are they druggable targets? Annu Rev Pharm Toxicol. 2023;63:19–41. 10.1146/annurev-pharmtox-051921-013755 36151054

[18] Rosato AS, Tang R, Grimm C. Two-pore and TRPML cation channels: Regulators of phagocytosis, autophagy and lysosomal exocytosis. Pharmacol Ther. 2021;220:107713. 33141027 10.1016/j.pharmthera.2020.107713

[19] Xiong J, Zhu MX. Regulation of lysosomal ion homeostasis by channels and transporters. Sci China Life Sci. 2016;59:777–91. 27430889 10.1007/s11427-016-5090-x PMC5147046

[20] Siintola E, Topcu M, Aula N, Lohi H, Minassian BA, Paterson AD, et al. The novel neuronal ceroid lipofuscinosis gene MFSD8 encodes a putative lysosomal transporter. Am J Hum Genet. 2007;81:136–46. 17564970 10.1086/518902 PMC1950917

[21] Wang Y, Zeng W, Lin B, Yao Y, Li C, Hu W, et al. CLN7 is an organellar chloride channel regulating lysosomal function. Sci Adv. 2021;7:eabj9608. 34910516 10.1126/sciadv.abj9608 PMC8673761

[22] Fan C, Wu H, Du X, Li C, Zeng W, Qu L, et al. Inhibition of lysosomal TRPML1 channel eliminates breast cancer stem cells by triggering ferroptosis. Cell Death Discov. 2024;10:256. 38802335 10.1038/s41420-024-02026-y PMC11130215

[23] Miao Z, Li J, Wang Y, Shi M, Gu X, Zhang X, et al. Hsa_circ_0136666 stimulates gastric cancer progression and tumor immune escape by regulating the miR-375/PRKDC Axis and PD-L1 phosphorylation. Mol Cancer. 2023;22:205. 38093288 10.1186/s12943-023-01883-y PMC10718020

[24] Geisslinger F, Muller M, Chao YK, Grimm C, Vollmar AM, Bartel K. Targeting TPC2 sensitizes acute lymphoblastic leukemia cells to chemotherapeutics by impairing lysosomal function. Cell Death Dis. 2022;13:668. 35915060 10.1038/s41419-022-05105-z PMC9343397

[25] Aits S, Jaattela M. Lysosomal cell death at a glance. J Cell Sci. 2013;126:1905–12. 23720375 10.1242/jcs.091181

[26] Jovanovic M, Rooney MS, Mertins P, Przybylski D, Chevrier N, Satija R, et al. Immunogenetics. Dynamic profiling of the protein life cycle in response to pathogens. Science. 2015;347:1259038. 25745177 10.1126/science.1259038 PMC4506746

[27] Sardiello M, Palmieri M, di Ronza A, Medina DL, Valenza M, Gennarino VA, et al. A gene network regulating lysosomal biogenesis and function. Science. 2009;325:473–7. 19556463 10.1126/science.1174447

[28] Puissant A, Colosetti P, Robert G, Cassuto JP, Raynaud S, Auberger P. Cathepsin B release after imatinib-mediated lysosomal membrane permeabilization triggers BCR-ABL cleavage and elimination of chronic myelogenous leukemia cells. Leukemia. 2010;24:115–24. 19924144 10.1038/leu.2009.233

[29] Galluzzi L, Baehrecke EH, Ballabio A, Boya P, Bravo-San Pedro JM, Cecconi F, et al. Molecular definitions of autophagy and related processes. EMBO J. 2017;36:1811–36. 28596378 10.15252/embj.201796697 PMC5494474

[30] Bourdenx M, Martin-Segura A, Scrivo A, Rodriguez-Navarro JA, Kaushik S, Tasset I, et al. Chaperone-mediated autophagy prevents collapse of the neuronal metastable proteome. Cell. 2021;184:2696–714. e25. 33891876 10.1016/j.cell.2021.03.048 PMC8152331

[31] Saxton RA, Sabatini DM. mTOR signaling in growth, metabolism, and disease. Cell. 2017;169:361–71. 28388417 10.1016/j.cell.2017.03.035

[32] Nnah IC, Wang B, Saqcena C, Weber GF, Bonder EM, Bagley D, et al. TFEB-driven endocytosis coordinates MTORC1 signaling and autophagy. Autophagy. 2019;15:151–64. 30145926 10.1080/15548627.2018.1511504 PMC6287686

[33] Yang J, Yuan L, Li L, Liu F, Liu J, Chen Y, et al. Trehalose activates autophagy to alleviate cisplatin-induced chronic kidney injury by targeting the mTOR-dependent TFEB signaling pathway. Theranostics. 2025;15:2544–63. 39990216 10.7150/thno.102559 PMC11840734

[34] Sun X, Yang Y, Zhong XZ, Cao Q, Zhu XH, Zhu X, et al. A negative feedback regulation of MTORC1 activity by the lysosomal Ca^2+^ channel MCOLN1 (mucolipin 1) using a CALM (calmodulin)-dependent mechanism. Autophagy. 2018;14:38–52. 29460684 10.1080/15548627.2017.1389822 PMC5846559

[35] Li RJ, Xu J, Fu C, Zhang J, Zheng YG, Jia H, et al. Regulation of mTORC1 by lysosomal calcium and calmodulin. Elife. 2016;5:e19360. 10.7554/eLife.19360 PMC5106211 27787197

[36] Young MM, Takahashi Y, Khan O, Park S, Hori T, Yun J, et al. Autophagosomal membrane serves as platform for intracellular death-inducing signaling complex (iDISC)-mediated caspase-8 activation and apoptosis. J Biol Chem. 2012;287:12455–68. 22362782 10.1074/jbc.M111.309104 PMC3320995

[37] Dai M, Lin B, Li H, Wang Y, Wu M, Wei Y, et al. Lysosomal cation channel TRPML1 suppression sensitizes acute myeloid leukemia cells to chemotherapeutics by inhibiting autophagy. Mol Cell Biochem. 2025;480:1209–24. 38951379 10.1007/s11010-024-05054-5

[38] Müller M, Geisslinger F, Gerndt S, Schütz R, Chao Y-K, Bracher F, et al. Blocking lysosomal two-pore channel 2 function inhibits proliferation of multidrug resistant leukemia cells and sensitizes them to vincristine treatment. Blood. 2019;134:2081.

[39] Tsukahara F, Maru Y. Bag1 directly routes immature BCR-ABL for proteasomal degradation. Blood. 2010;116:3582–92. 20675402 10.1182/blood-2009-10-249623

[40] Melo JV, Hewett DR. Wrapping BCR-ABL: it’s in the bag. Blood. 2010;116:3382–3. 21051563 10.1182/blood-2010-08-300608

[41] Yang Y, Gao H, Sun X, Sun Y, Qiu Y, Weng Q, et al. Global PROTAC toolbox for degrading BCR-ABL overcomes drug-resistant mutants and adverse effects. J Med Chem. 2020;63:8567–83. 32657579 10.1021/acs.jmedchem.0c00967

[42] Lai AC, Toure M, Hellerschmied D, Salami J, Jaime-Figueroa S, Ko E, et al. Modular PROTAC design for the degradation of oncogenic BCR-ABL. Angew Chem Int Ed Engl. 2016;55:807–10. 26593377 10.1002/anie.201507634 PMC4733637

[43] Jiang L, Wang Y, Li Q, Tu Z, Zhu S, Tu S, et al. Design, synthesis, and biological evaluation of Bcr-Abl PROTACs to overcome T315I mutation. Acta Pharmacol Sin B. 2021;11:1315–28. 10.1016/j.apsb.2020.11.009 PMC8148061 34094836

[44] Wang W, Xia Z, Farre JC, Subramani S. TRIM37 deficiency induces autophagy through deregulating the MTORC1-TFEB axis. Autophagy. 2018;14:1574–85. 29940807 10.1080/15548627.2018.1463120 PMC6135569

[45] Cui Z, Napolitano G, de Araujo MEG, Esposito A, Monfregola J, Huber LA, et al. Structure of the lysosomal mTORC1-TFEB-Rag-Ragulator megacomplex. Nature. 2023;614:572–9. 36697823 10.1038/s41586-022-05652-7 PMC9931586

[46] Mi XJ, Choi HS, Perumalsamy H, Shanmugam R, Thangavelu L, Balusamy SR, et al. Biosynthesis and cytotoxic effect of silymarin-functionalized selenium nanoparticles induced autophagy-mediated cellular apoptosis via downregulation of PI3K/Akt/mTOR pathway in gastric cancer. Phytomedicine. 2022;99:154014. 35247670 10.1016/j.phymed.2022.154014

[47] Hu HF, Fu JY, Han L, Gao GB, Zhang WX, Yu SM, et al. The antipsychotic drug aripiprazole suppresses colorectal cancer by targeting LAMP2a to induce RNH1/miR-99a/mTOR-mediated autophagy and apoptosis. Adv Sci. 2024;11:e2409498. 10.1002/advs.202409498 PMC11672294 39513392

