# Supplementary material for: CLN7 suppression induces apoptosis via mTOR-regulated and chaperone-mediated autophagy in myeloid leukemia cells
Source: Cell Death Dis. 2026 May 30;17(1):672. doi: 10.1038/s41419-026-08936-2 (PMC13429735; doi:10.1038/s41419-026-08936-2)
Supplement: Supplementary file 1 — SI-CLN7 suppression induces apoptosis via mTOR-regulated and chaperone-mediated autophagy in myeloid leukemia cells [file 41419_2026_8936_MOESM1_ESM.docx]

**CLN7 suppression induces apoptosis via mTOR-regulated and chaperone-mediated autophagy in myeloid leukemia cells**


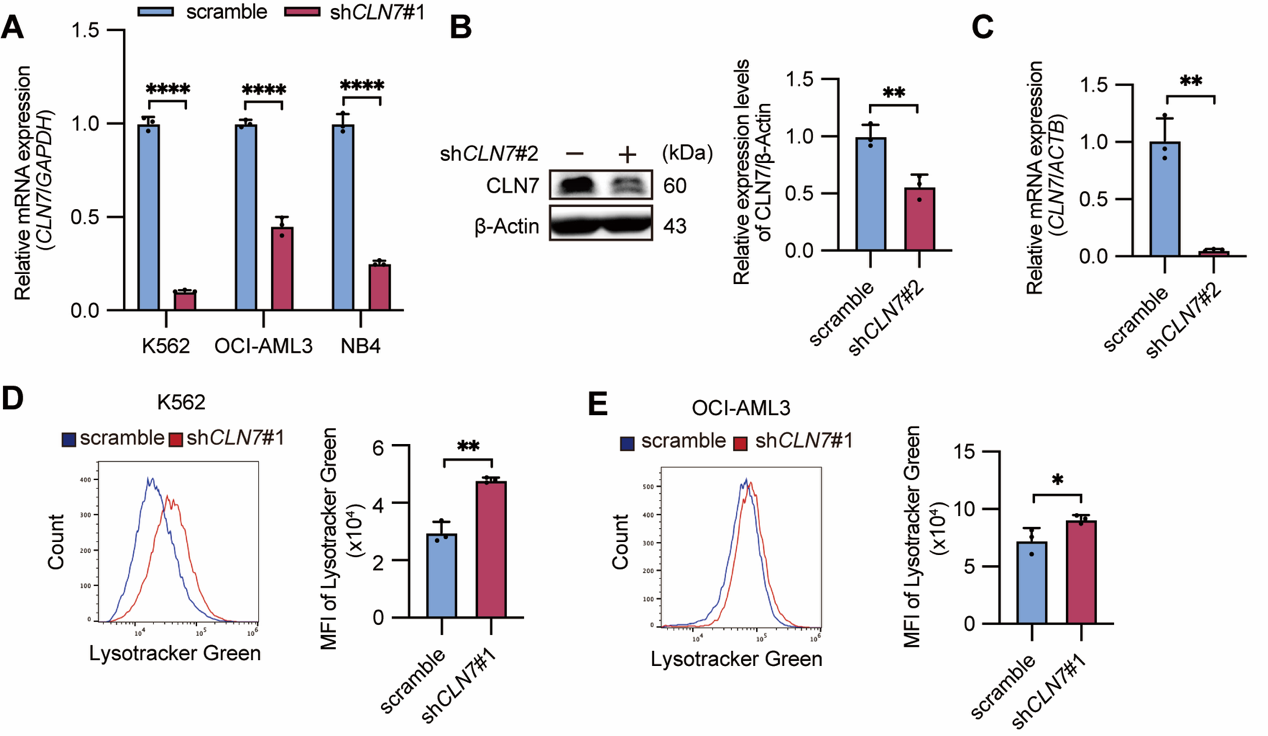


**Fig. S1 CLN7 is highly expressed in myeloid leukemia and modulates lysosomal function.** (A) RT-qPCR analysis of the mRNA expression of *CLN7* in scramble and sh*CLN7*#1 cells of K562, OCI-AML3 and NB4 cells (n=3). (B) Western blot analysis of CLN7 protein expression levels in scramble and sh*CLN7*#2 cells of K562 (n=3). (C) RT-qPCR analysis of the mRNA expression of *CLN7* in scramble and sh*CLN7*#2 cells of K562 (n=3). (D, E) Flow cytometry analysis of lysosome changes (Lysotracker Green, 100 nM) in K562 and OCI-AML3 scramble and sh*CLN7*#1 cells (n=3). Data are shown as mean ± SD. *p < 0.05, **p < 0.01, **** p < 0.0001.


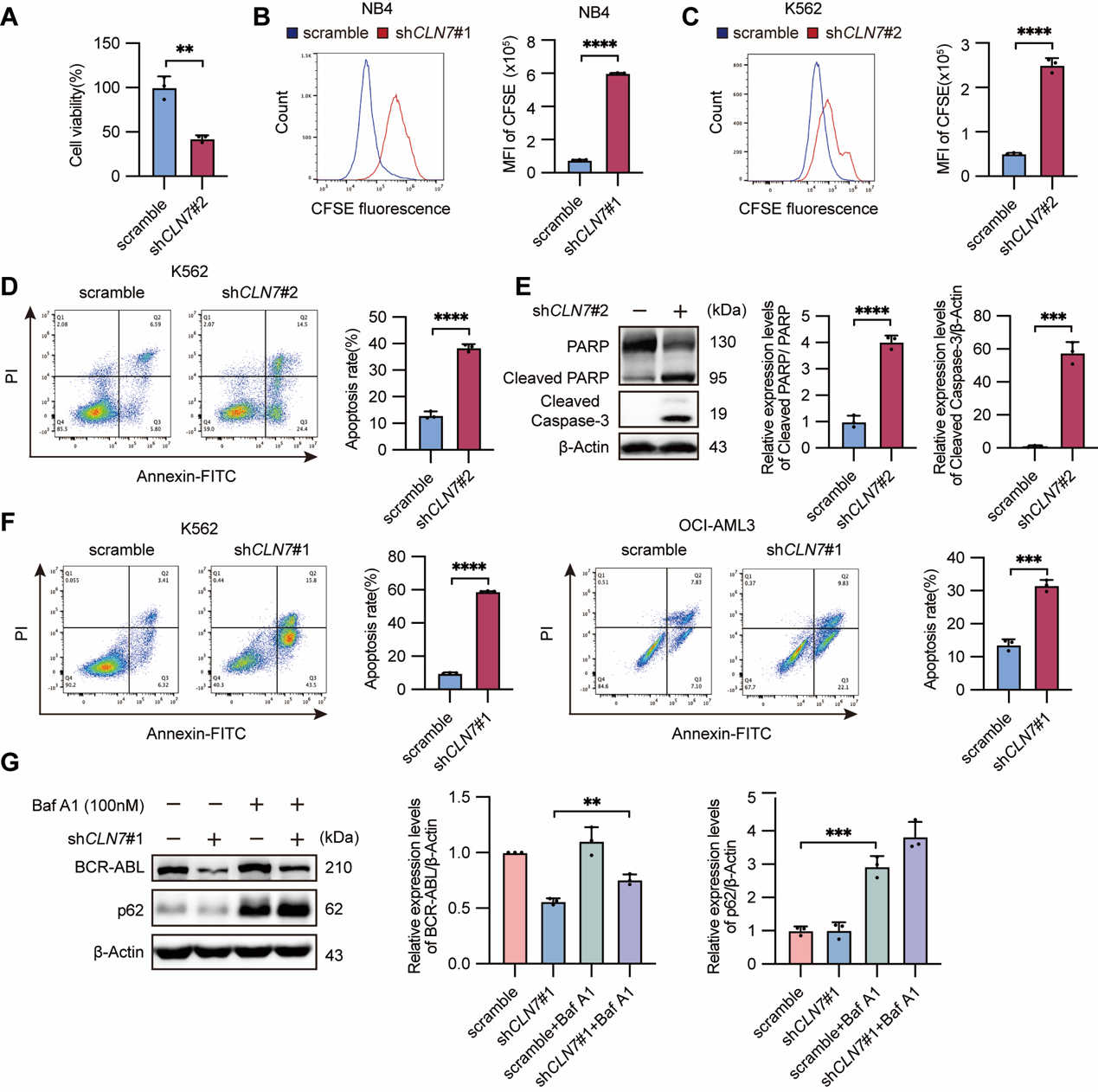


**Fig. S2** **CLN7 is essential for proliferation and survival of leukemia cells.** (A) Cell viability was measured using CCK8 in scramble and sh*CLN7*#2 of K562 cells (n=3). (B) Flow cytometry analysis of CFSE staining of scramble and sh*CLN7*#1 cells in NB4 (n=3). (C) Flow cytometry analysis of CFSE staining in scramble and sh*CLN7*#2 cells in K562 (n=3). (D) Flow cytometry analysis of apoptosis of scramble and sh*CLN7*#2 cells of K562 at third day after virus infection (n=3). (E) Western blot analysis of apoptosis-related protein PARP, Cleaved Caspase-3 expression levels of K562 scramble and sh*CLN7*#2 cells (n=3). (F) Flow cytometry analysis of apoptosis of scramble and sh*CLN7*#1 cells in K562 and OCI-AML3 at fifth day after virus infection (n=3). (G) Western blot analysis of BCR-ABL and p62 protein expression levels of K562 scramble and sh*CLN7*#1 cells treated with or without Baf A1 (100 nM) for 24 h (n=3). Data are shown as mean ± SD. **p < 0.01, ***p < 0.001, **** p < 0.0001.


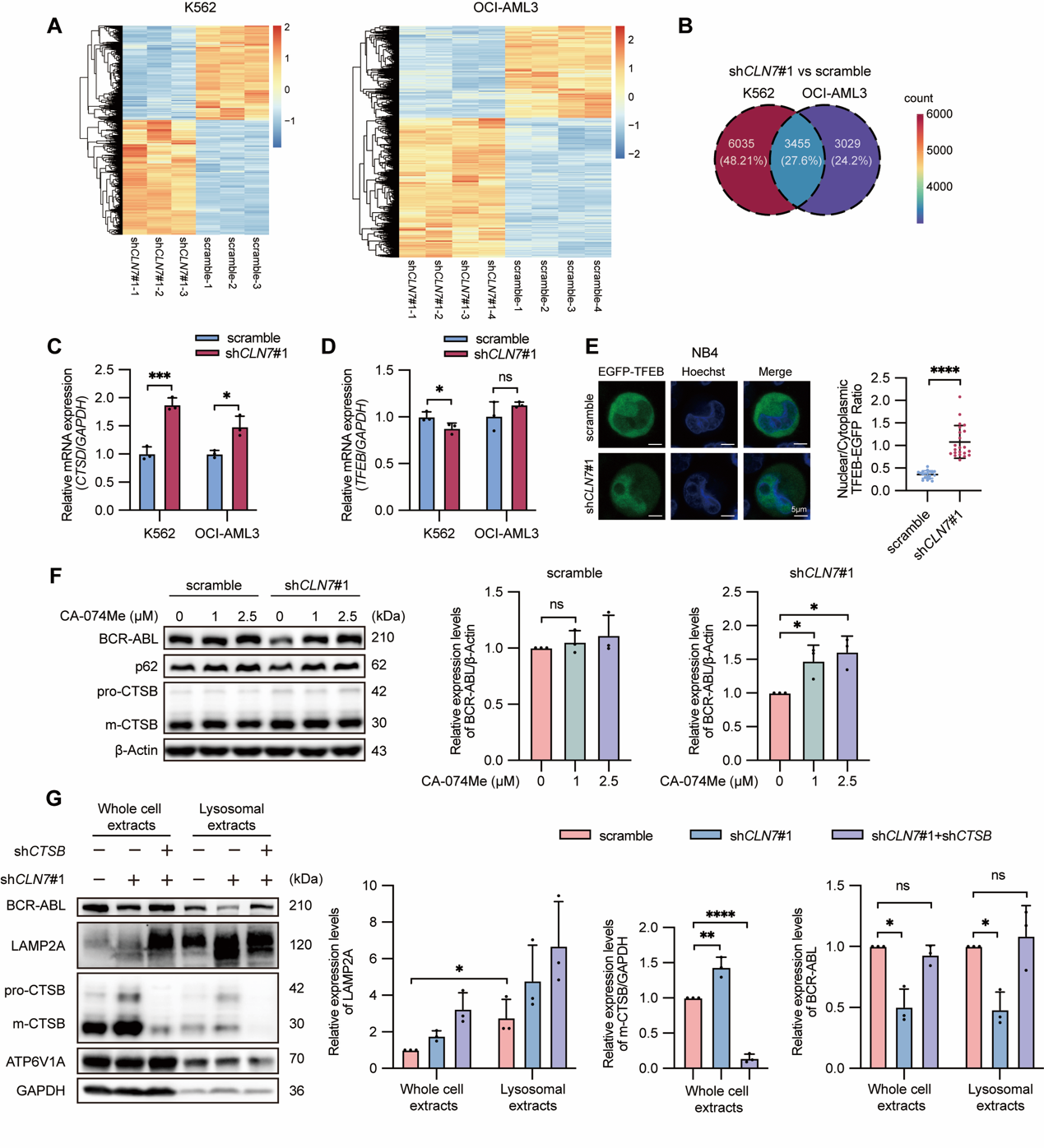


**Fig. S3** **Inhibition of CLN7 enhances lysosomal activity and induces BCR-ABL degradation.** (A) Heatmap showing the differential gene expression between sh*CLN7*#1 and scramble groups of K562 (n=3) and OCI-AML3 (n=4). (B) Venn diagram showing DEGs overlap between K562 and OCI-AML3. (C, D) RT-qPCR analysis of the *CTSD* and *TFEB* mRNA expression levels of scramble and sh*CLN7*#1 in K562 and OCI-AML3 cells (n=3). (E) Confocal imaging analysis of EGFP-TFEB nuclear translocation in scramble (n=19) and sh*CLN7*#1 (n=22) cells of NB4. Scale bars, 5 μm. (F) Western blot analysis of BCR-ABL, p62 and CTSB protein expression levels of K562 scramble and sh*CLN7*#1 cells treated with or without CTSB inhibitor (CA-074Me, 1 μM and 2.5 μM) for 24 h (n=3). (G) Western blot analysis of whole-cell extracts and lysosomal extracts prepared from K562 scramble, *CLN7* knockdown (sh*CLN7*#1) and *CLN7* and *CTSB* combined knockdown (sh*CLN7*#1+shCTSB) cells against BCR-ABL, LAMP2A, CTSB, ATP6V1A (lysosomal marker) and GAPDH (whole cell marker) (n=3). Data are shown as mean ± SD. *p < 0.05, **p < 0.01, ***p < 0.001, **** p < 0.0001, ns, not significant.


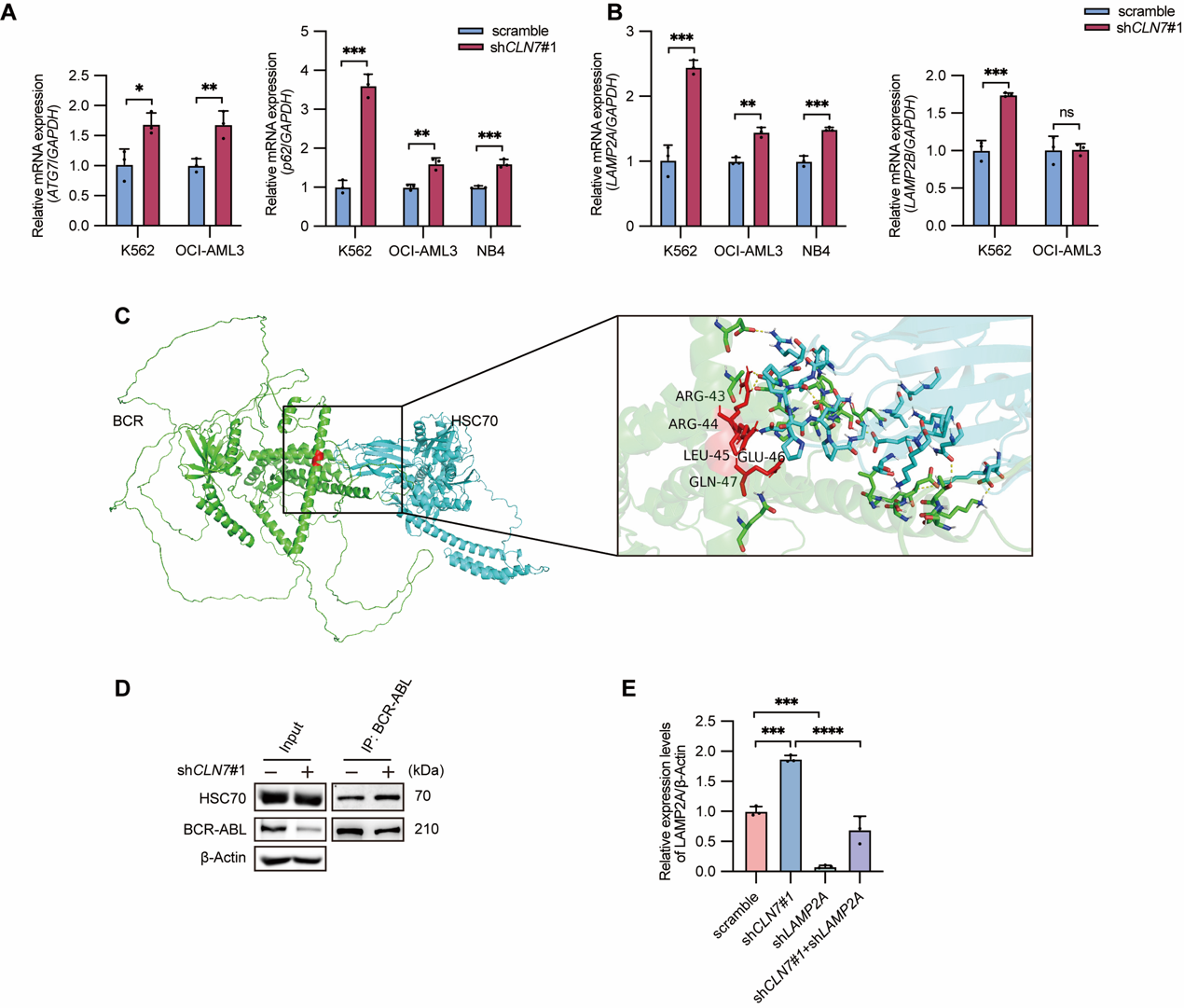


**Fig. S4 CLN7 suppression activates autophagy and accelerates chaperone-mediated autophagic degradation of BCR-ABL.** (A, B) RT-qPCR analysis of the *ATG7*, *p62*, *LAMP2A* and *LAMP2B* mRNA expression levels of scramble and sh*CLN7*#1 in K562 and OCI-AML3 cells. (C) Close-up of the BCR-HSC70 docking interface. The inset focuses on the binding of the RRLEQ motif (red sticks, residues 43–47) from the BCR domain (green) within the HSC70 pocket (cyan). Interacting residues are highlighted as sticks, with potential hydrogen bonds indicated by dashed lines. This interaction was further visualized in PyMOL. (D) Interaction of HSC70 and BCR-ABL detected by Co-IP and Western blot from K562 scramble and sh*CLN7*#1 cells. (E) Statistics of LAMP2A protein expression levels in (Fig. 4E). Data are shown as mean ± SD. *p < 0.05, **p < 0.01, ***p < 0.001, **** p < 0.0001, ns, not significant.


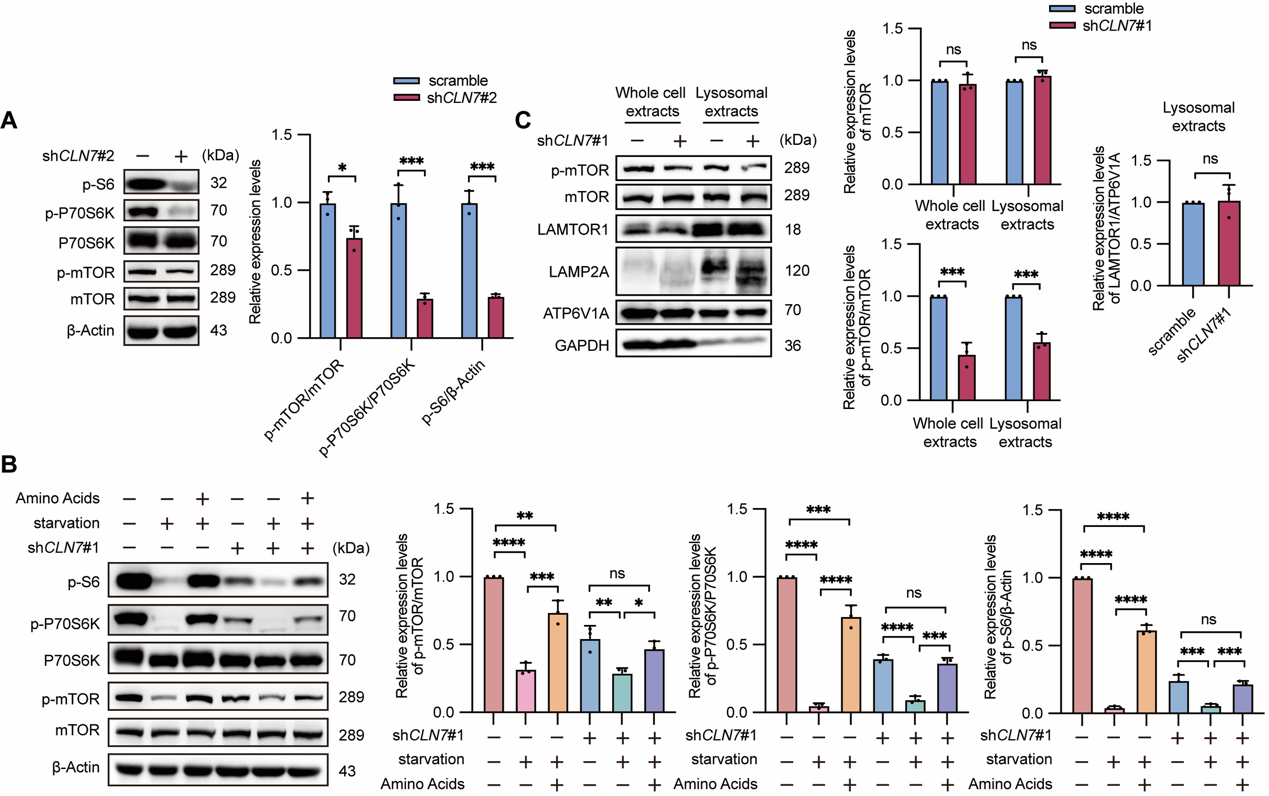


**Fig. S5 mTOR-mediated autophagy activation drives apoptosis following CLN7 suppression.** (A) Western blot analysis of p-S6, p-P70S6K and P70S6K, p-mTOR and mTOR protein expression levels in K562 scramble and sh*CLN7*#2 cells (n=3). (B) Western blot analysis of p-S6, p-P70S6K and P70S6K, p-mTOR and mTOR protein expression levels in K562 scramble and sh*CLN7*#1 cells. Cells were starved in AA-free medium (4 h) and then stimulated with AA-complete medium (30 min) for nutrient-recovery analysis (n=3). (C) Western blot analysis of whole-cell extracts and lysosomal extracts prepared from K562 scramble and sh*CLN7*#1 cells against p-mTOR, mTOR, LAMTOR1, LAMP2A, ATP6V1A (lysosomal marker) and GAPDH (whole cell marker) (n=3). Data are shown as mean ± SD. *p < 0.05, **p < 0.01, ***p < 0.001, **** p < 0.0001, ns, not significant.

**
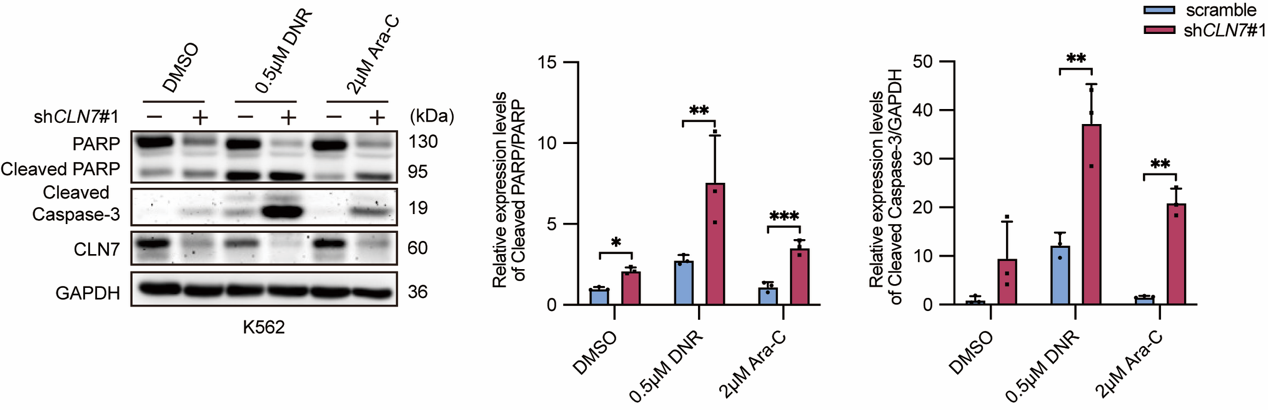
**

**Fig. S6 CLN7 suppression enhances the cytotoxic effects of chemotherapeutic agents in myeloid leukemia cells.** Western blot analysis of apoptosis-related protein PARP, Cleaved Caspase-3 expression levels in scramble and sh*CLN7*#1 cells of K562 treated with or without DNR (0.5 μM) or Ara-C (2 μM) for 48 h (n=3). Data are shown as mean ± SD. *p < 0.05, **p < 0.01, ***p < 0.001. DNR: Daunorubicin; Ara-C: cytarabine.


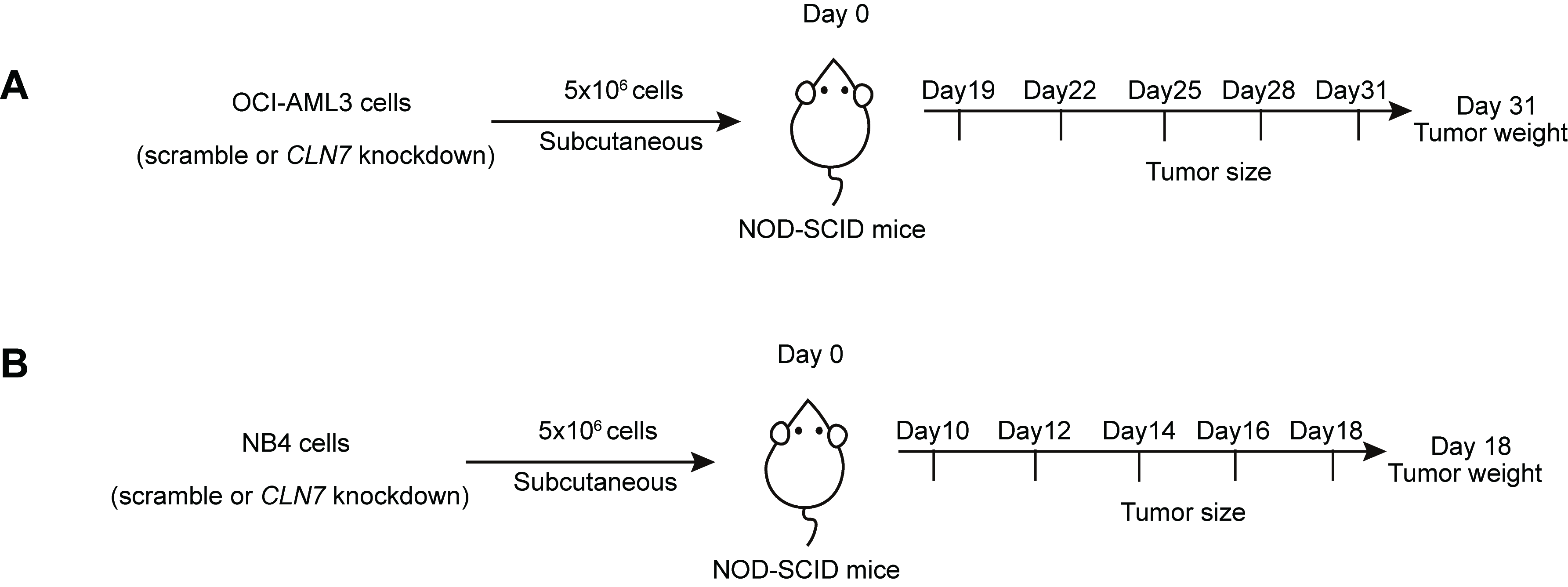


**Fig. S7 Inhibition of CLN7 suppresses tumor growth *in vivo.*** (A) 5 × 10^6^ OCI-AML3 scramble or sh*CLN7*#1 cells were subcutaneously injected into the right flanks of the NOD-SCID mice, followed by tumor size measurement. (B) 5 × 10^6^ NB4 scramble or sh*CLN7*#1 cells were subcutaneously injected into the right flanks of the NOD-SCID mice, followed by tumor size measurement.


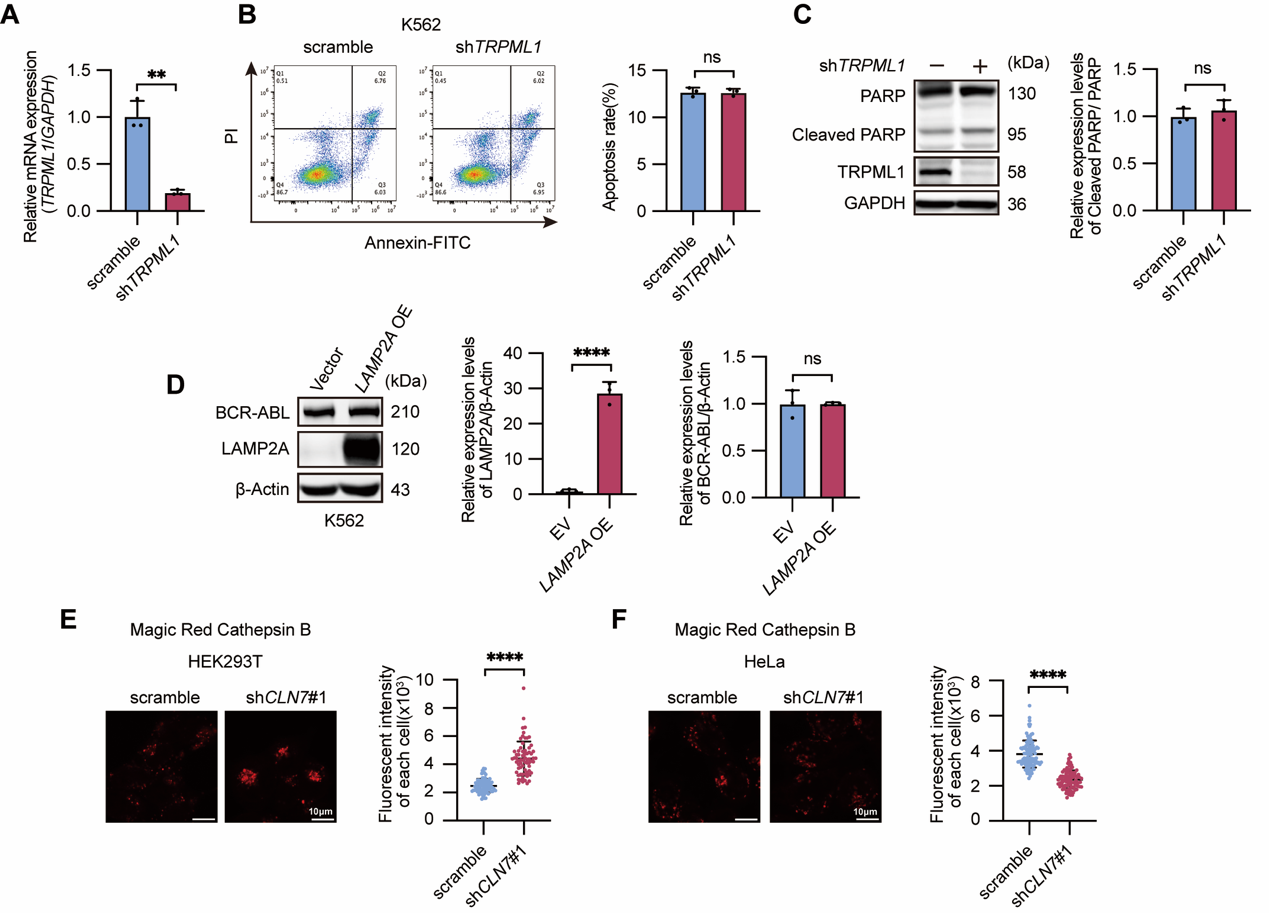


**Fig. S8** (A) RT-qPCR analysis of the mRNA expression of *TRPML1* in scramble and sh*TRPML1* cells of K562 (n=3). (B) Flow cytometry analysis of apoptosis of scramble and *TRPML1* knockdown cells of K562 (n=3). (C) Western blot analysis of apoptosis-related protein PARP (n=3), TRPML1 expression levels of K562 scramble and sh*TRPML1* cells. (D)Western blot analysis of LAMP2A and BCR-ABL protein expression levels in K562 transfected empty vector or LAMP2A (n=3). (E) Confocal imaging analysis of lysosome CTSB activity of scramble (n=77) and sh*CLN7*#1 (n=68) cells in HEK293T. Scale bars, 10 μm. (F) Confocal imaging analysis of lysosome CTSB activity of scramble (n=112) and sh*CLN7*#1 (n=91) cells in HeLa. Scale bars, 10 μm. Data are shown as mean ± SD. **p < 0.01, **** p < 0.0001, ns, not significant.
