# Supplementary material for: CLN7 suppression induces apoptosis via mTOR-regulated and chaperone-mediated autophagy in myeloid leukemia cells
Source: Cell Death Dis. 2026 May 30;17(1):672. doi: 10.1038/s41419-026-08936-2 (PMC13429735; doi:10.1038/s41419-026-08936-2)
Supplement: Supplementary file 2 — Original Western Blot [file 41419_2026_8936_MOESM2_ESM.pptx]

## Slide 1
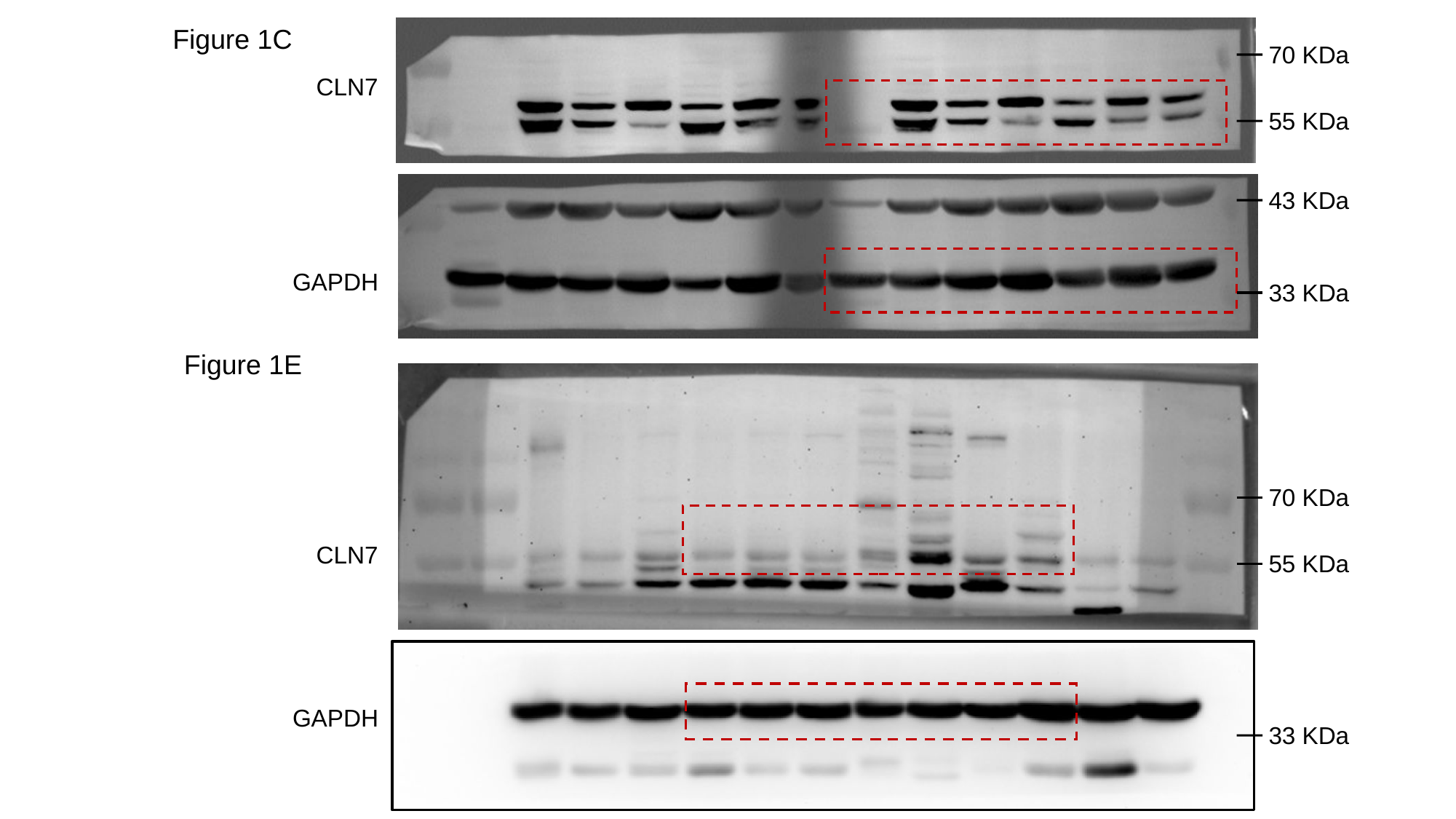

Figure 1C
70 KDa
CLN7
55 KDa
43 KDa
GAPDH
33 KDa
Figure 1E
70 KDa
CLN7
55 KDa
GAPDH
33 KDa

## Slide 2
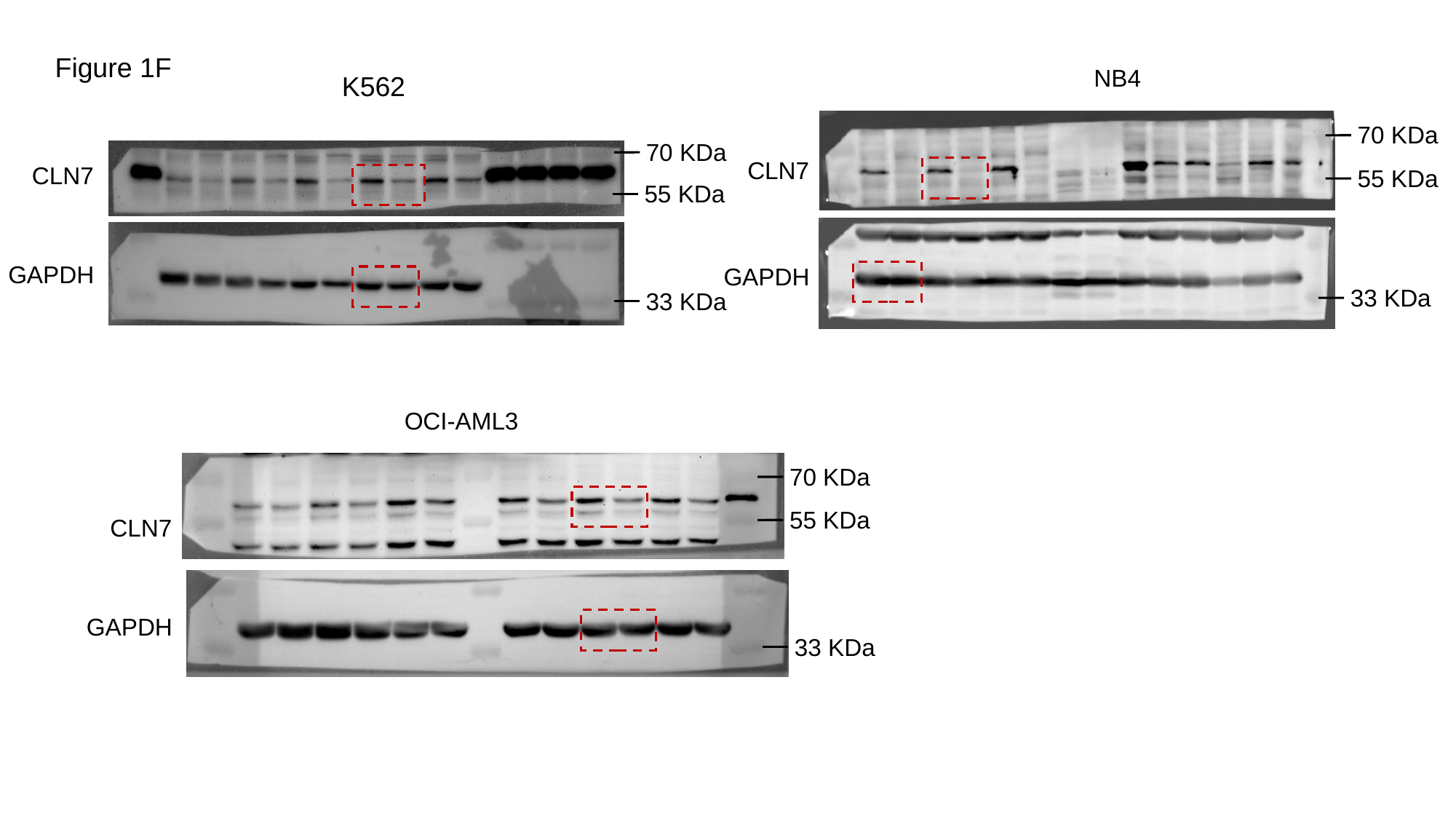

Figure 1F
NB4
K562
70 KDa
70 KDa
CLN7
CLN7
55 KDa
55 KDa
GAPDH
GAPDH
33 KDa
33 KDa
OCI-AML3
70 KDa
55 KDa
CLN7
GAPDH
33 KDa

## Slide 3
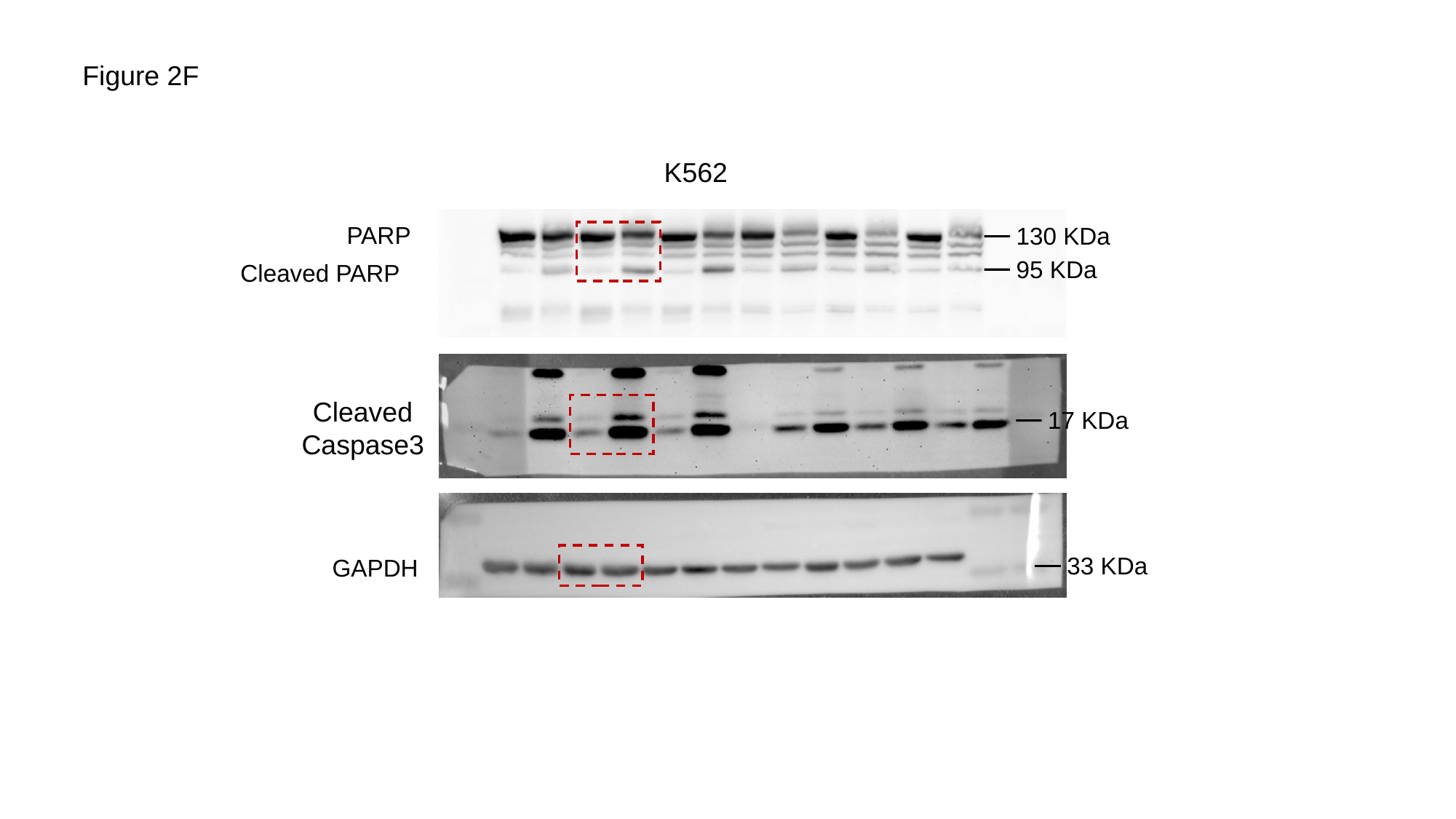

Figure 2F
K562
PARP
130 KDa
95 KDa
Cleaved PARP
Cleaved Caspase3
17 KDa
33 KDa
GAPDH

## Slide 4
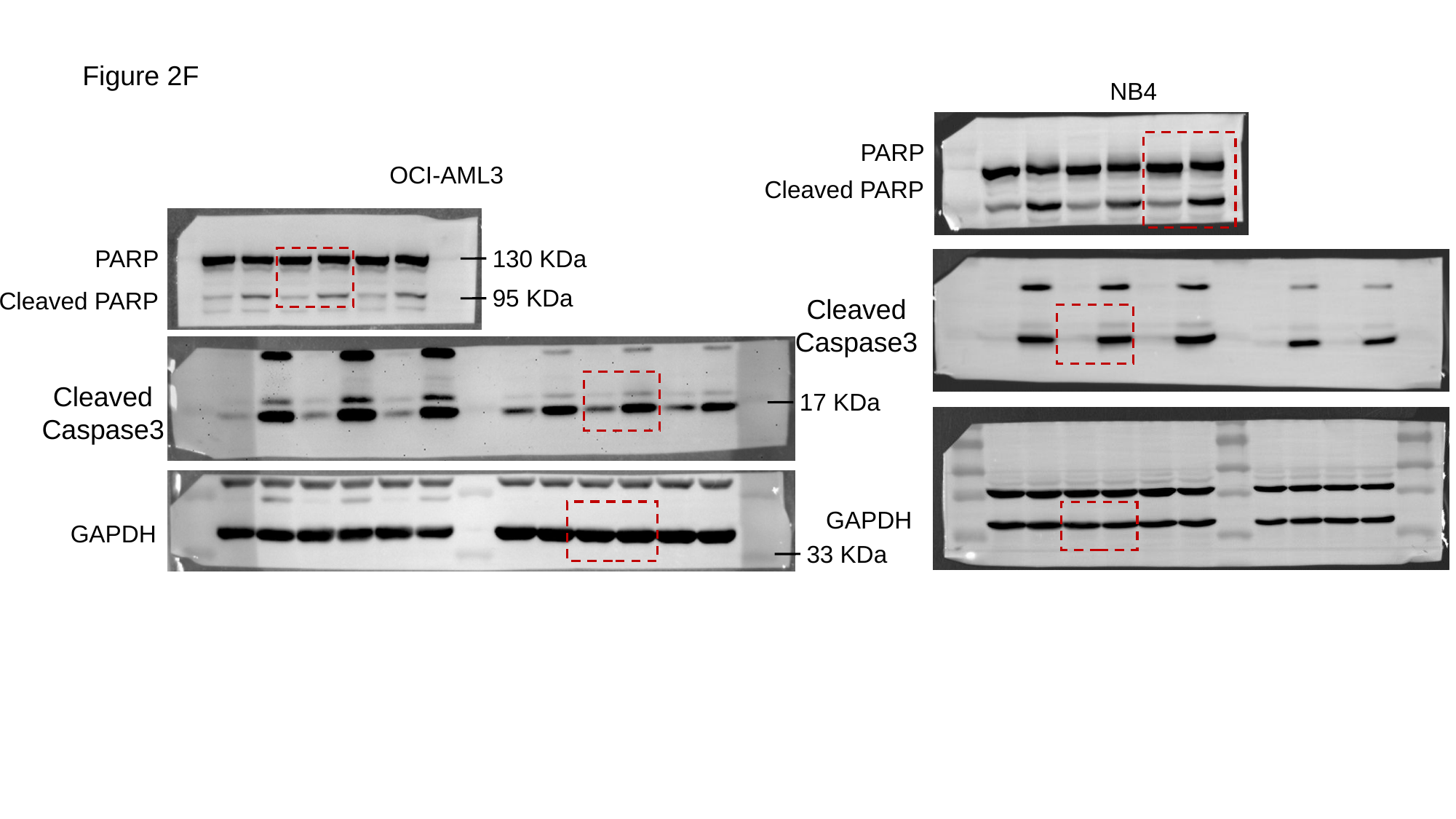

Figure 2F
NB4
PARP
OCI-AML3
Cleaved PARP
130 KDa
PARP
95 KDa
Cleaved PARP
Cleaved Caspase3
Cleaved Caspase3
17 KDa
GAPDH
GAPDH
33 KDa

## Slide 5
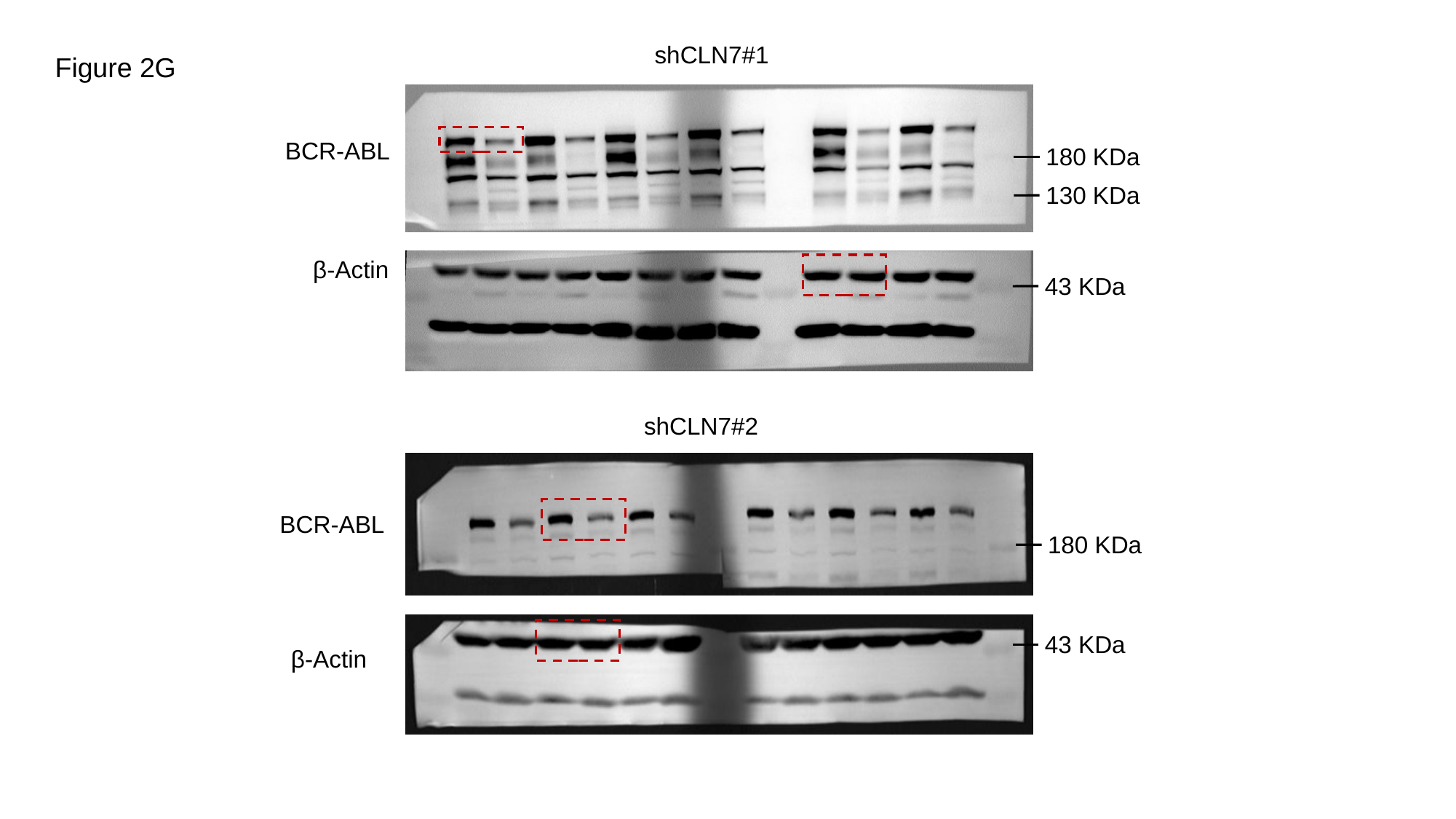

shCLN7#1
Figure 2G
BCR-ABL
180 KDa
130 KDa
β-Actin
43 KDa
shCLN7#2
BCR-ABL
180 KDa
43 KDa
β-Actin

## Slide 6
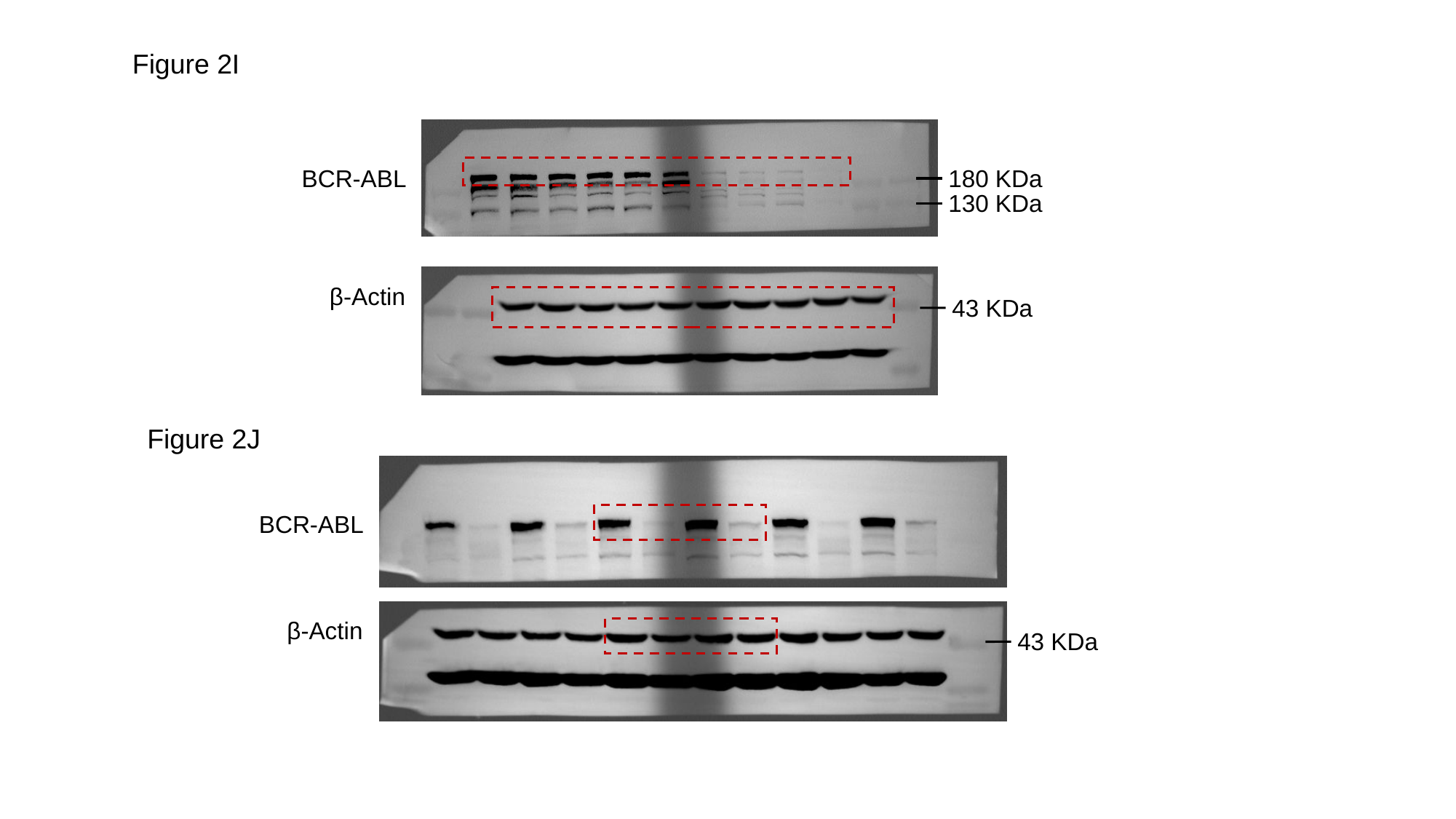

Figure 2I
BCR-ABL
180 KDa
130 KDa
β-Actin
43 KDa
Figure 2J
BCR-ABL
β-Actin
43 KDa

## Slide 7
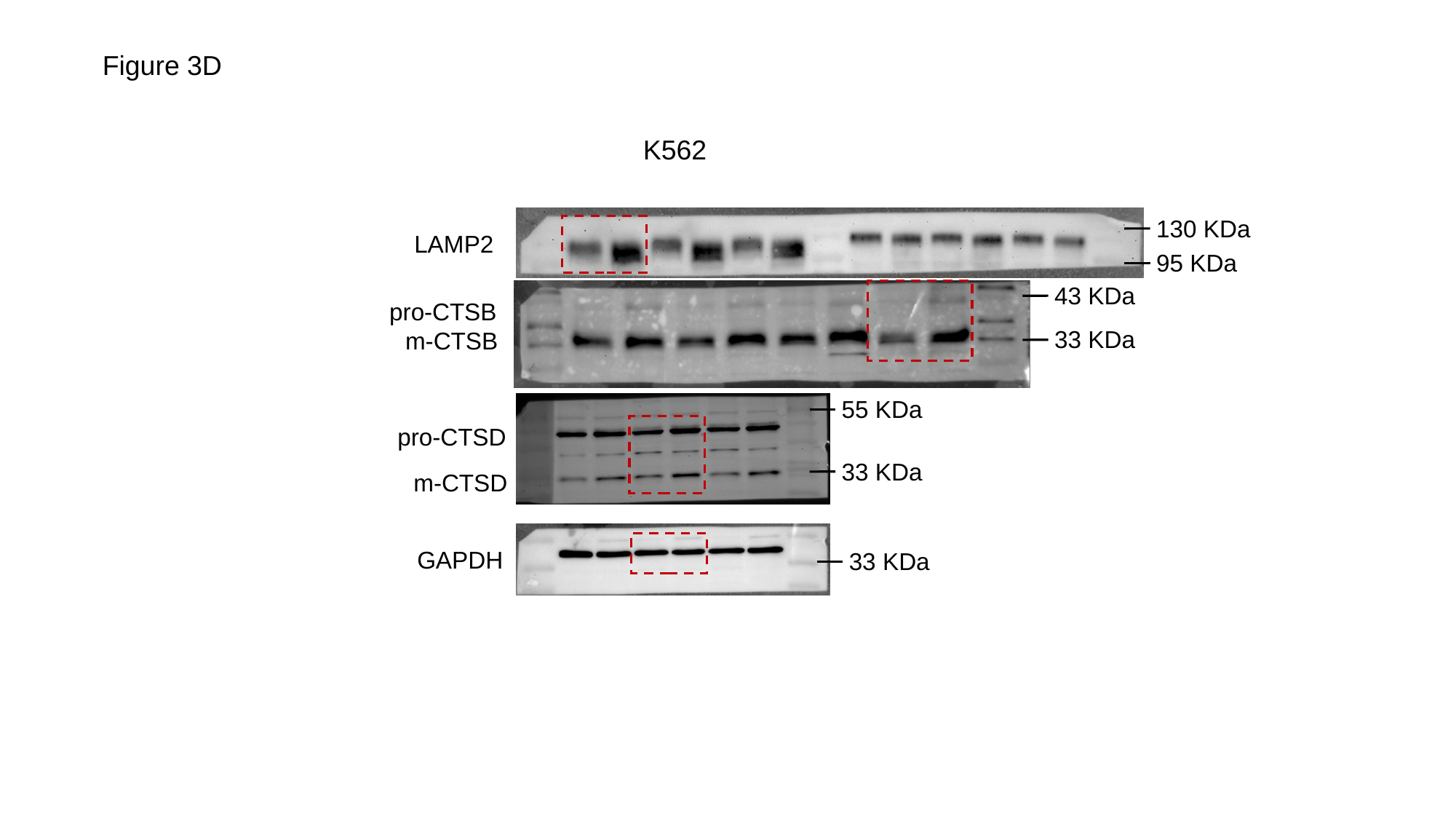

Figure 3D
K562
130 KDa
LAMP2
95 KDa
43 KDa
pro-CTSB
33 KDa
m-CTSB
55 KDa
pro-CTSD
33 KDa
m-CTSD
GAPDH
33 KDa

## Slide 8
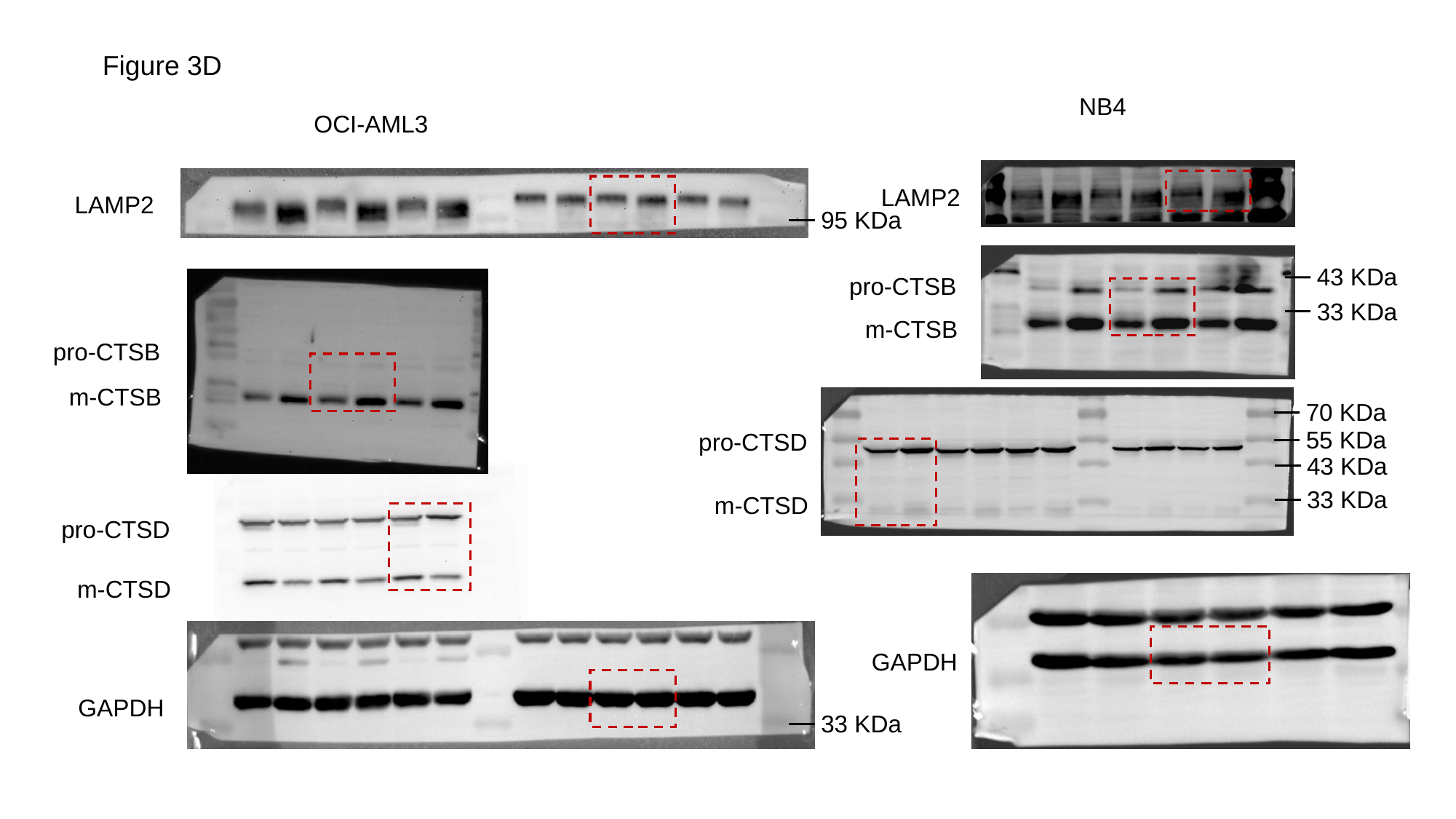

Figure 3D
NB4
OCI-AML3
LAMP2
LAMP2
95 KDa
43 KDa
pro-CTSB
33 KDa
m-CTSB
pro-CTSB
m-CTSB
70 KDa
55 KDa
pro-CTSD
43 KDa
33 KDa
m-CTSD
pro-CTSD
m-CTSD
GAPDH
GAPDH
33 KDa

## Slide 9
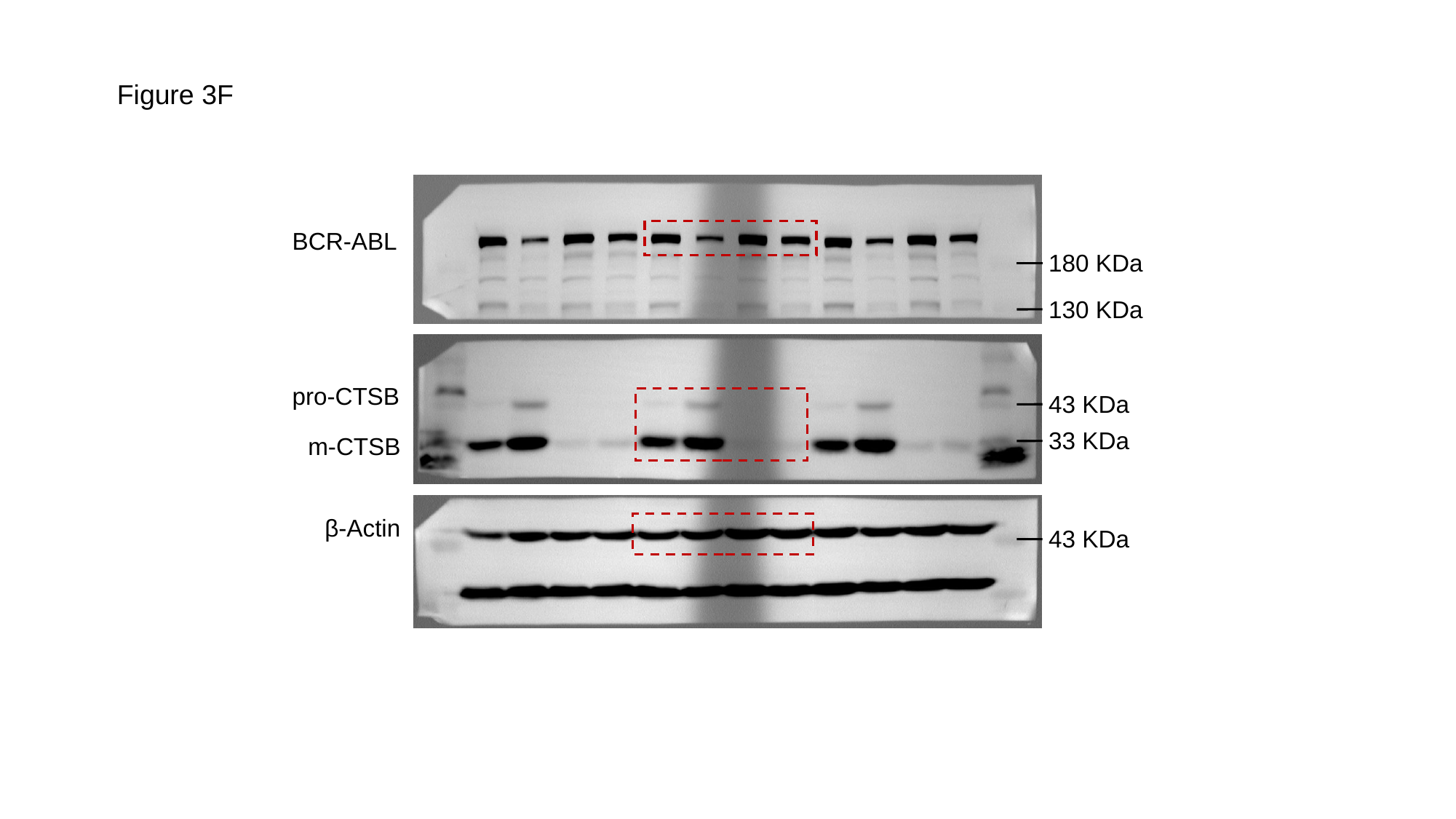

Figure 3F
BCR-ABL
180 KDa
130 KDa
pro-CTSB
43 KDa
33 KDa
m-CTSB
β-Actin
43 KDa

## Slide 10
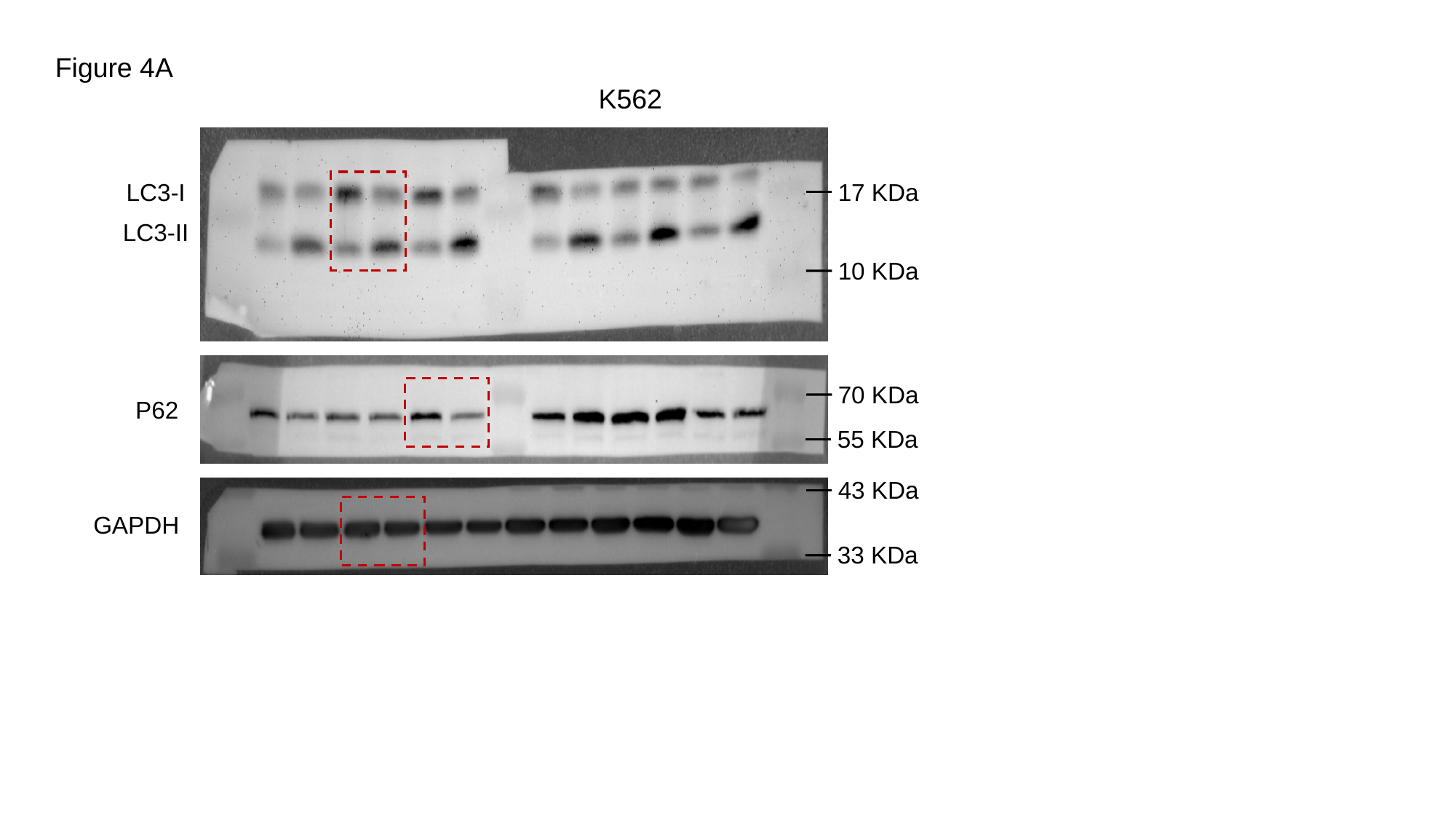

Figure 4A
K562
17 KDa
LC3-I
LC3-II
10 KDa
70 KDa
P62
55 KDa
43 KDa
GAPDH
33 KDa

## Slide 11
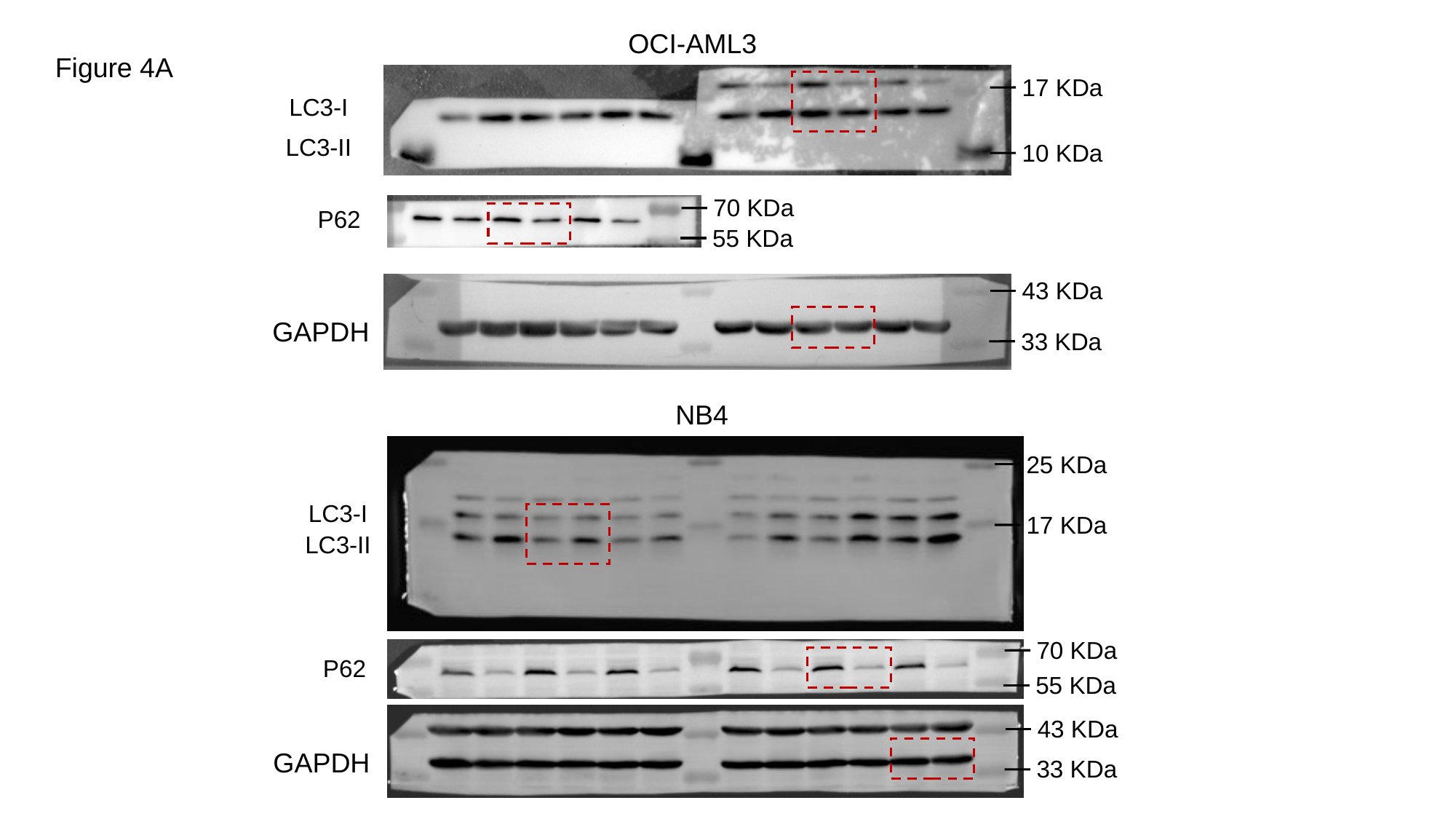

OCI-AML3
Figure 4A
17 KDa
LC3-I
LC3-II
10 KDa
70 KDa
P62
55 KDa
43 KDa
GAPDH
33 KDa
NB4
25 KDa
LC3-I
17 KDa
LC3-II
70 KDa
P62
55 KDa
43 KDa
GAPDH
33 KDa

## Slide 12
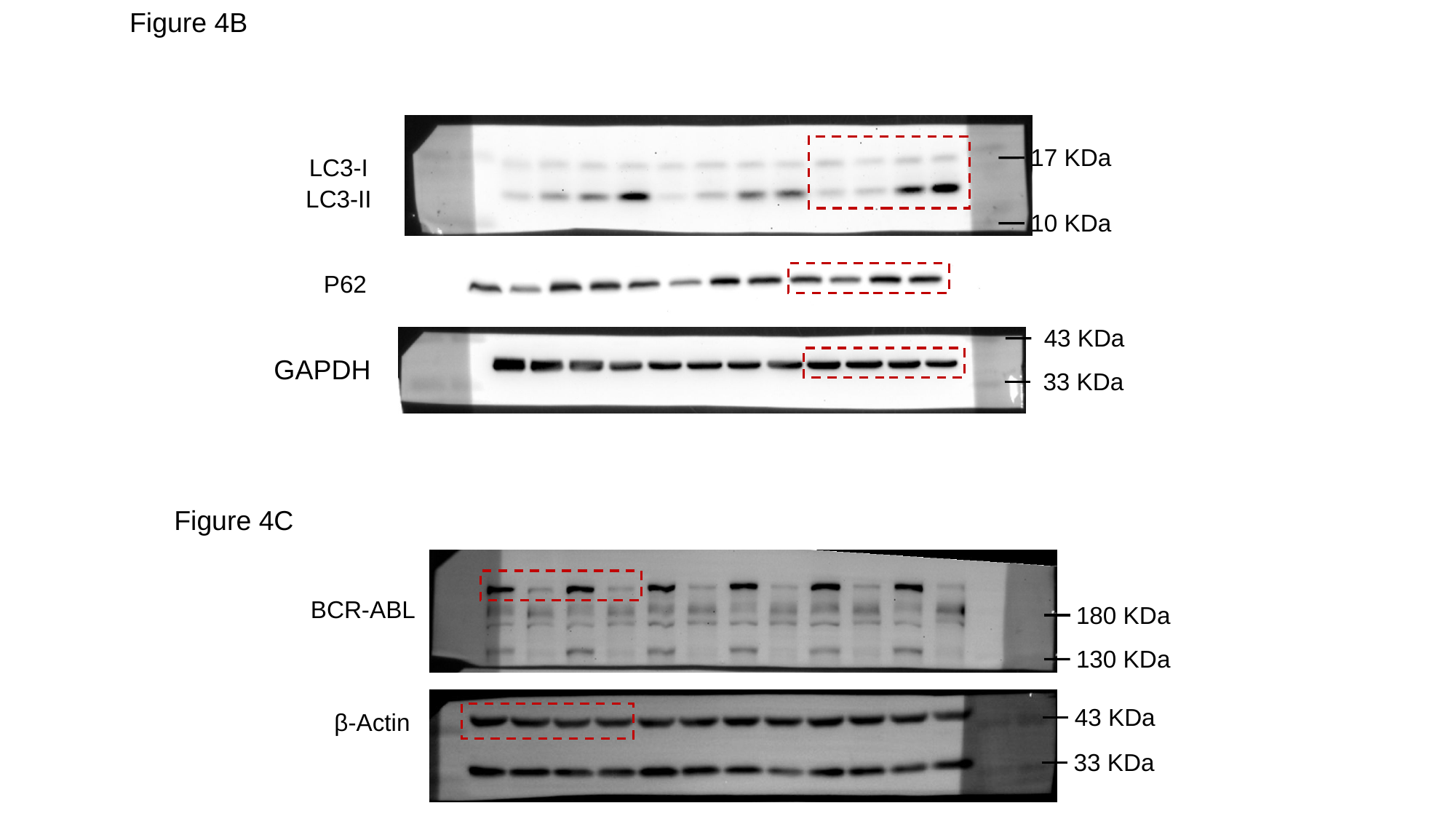

Figure 4B
17 KDa
LC3-I
LC3-II
10 KDa
P62
43 KDa
GAPDH
33 KDa
Figure 4C
BCR-ABL
180 KDa
130 KDa
43 KDa
β-Actin
33 KDa

## Slide 13
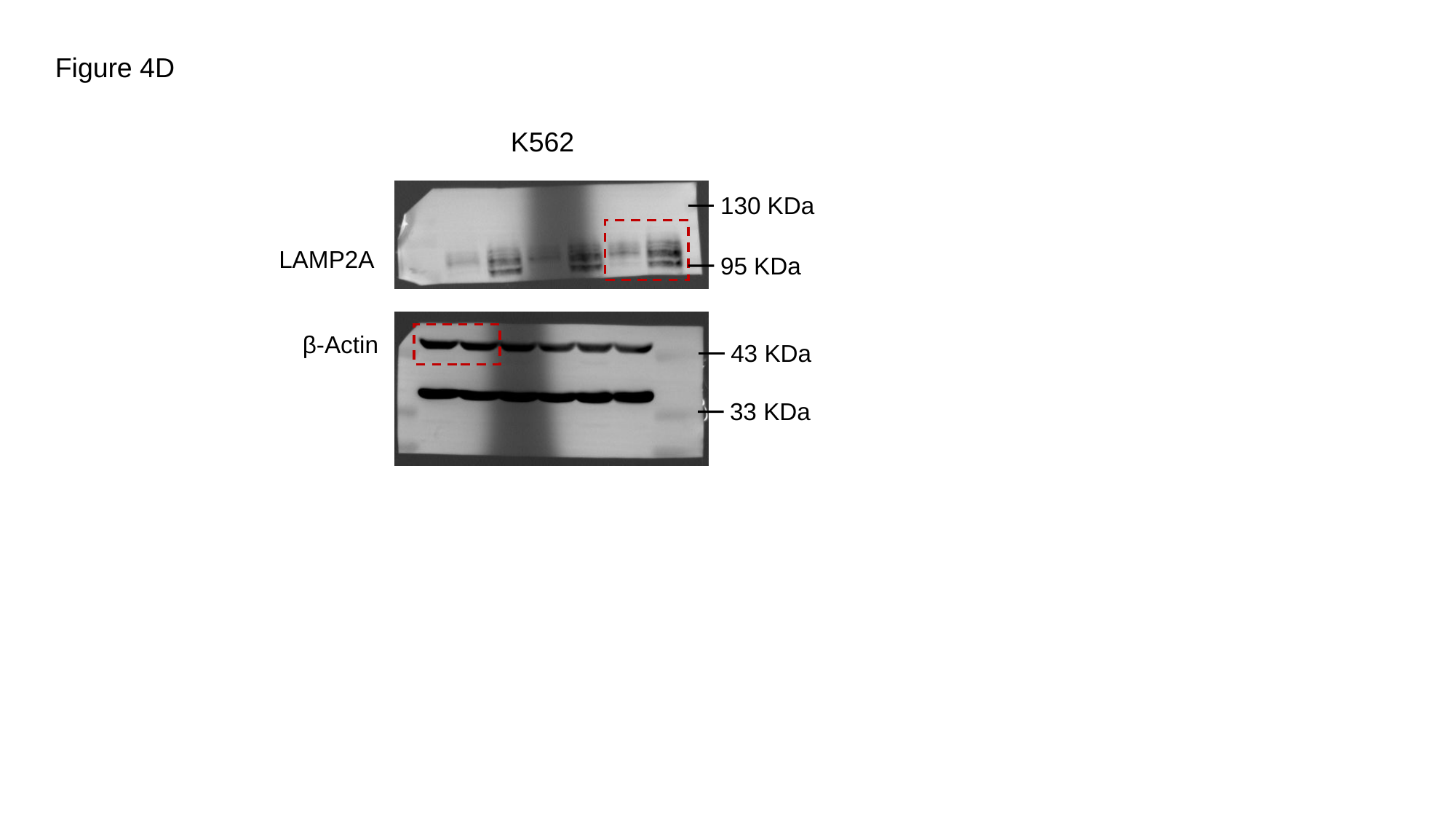

Figure 4D
K562
130 KDa
LAMP2A
95 KDa
β-Actin
43 KDa
33 KDa

## Slide 14
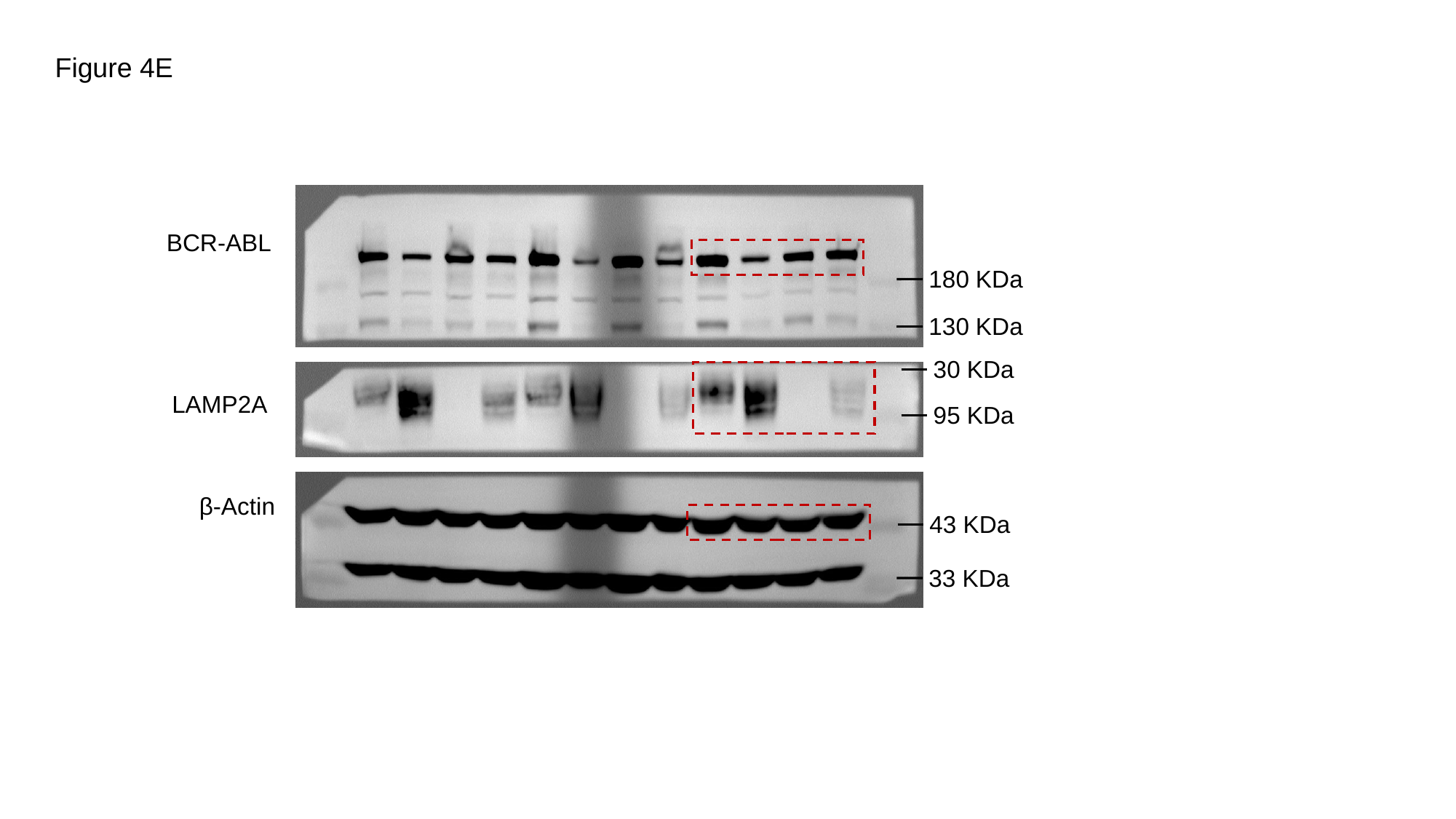

Figure 4E
BCR-ABL
180 KDa
130 KDa
30 KDa
LAMP2A
95 KDa
β-Actin
43 KDa
33 KDa

## Slide 15
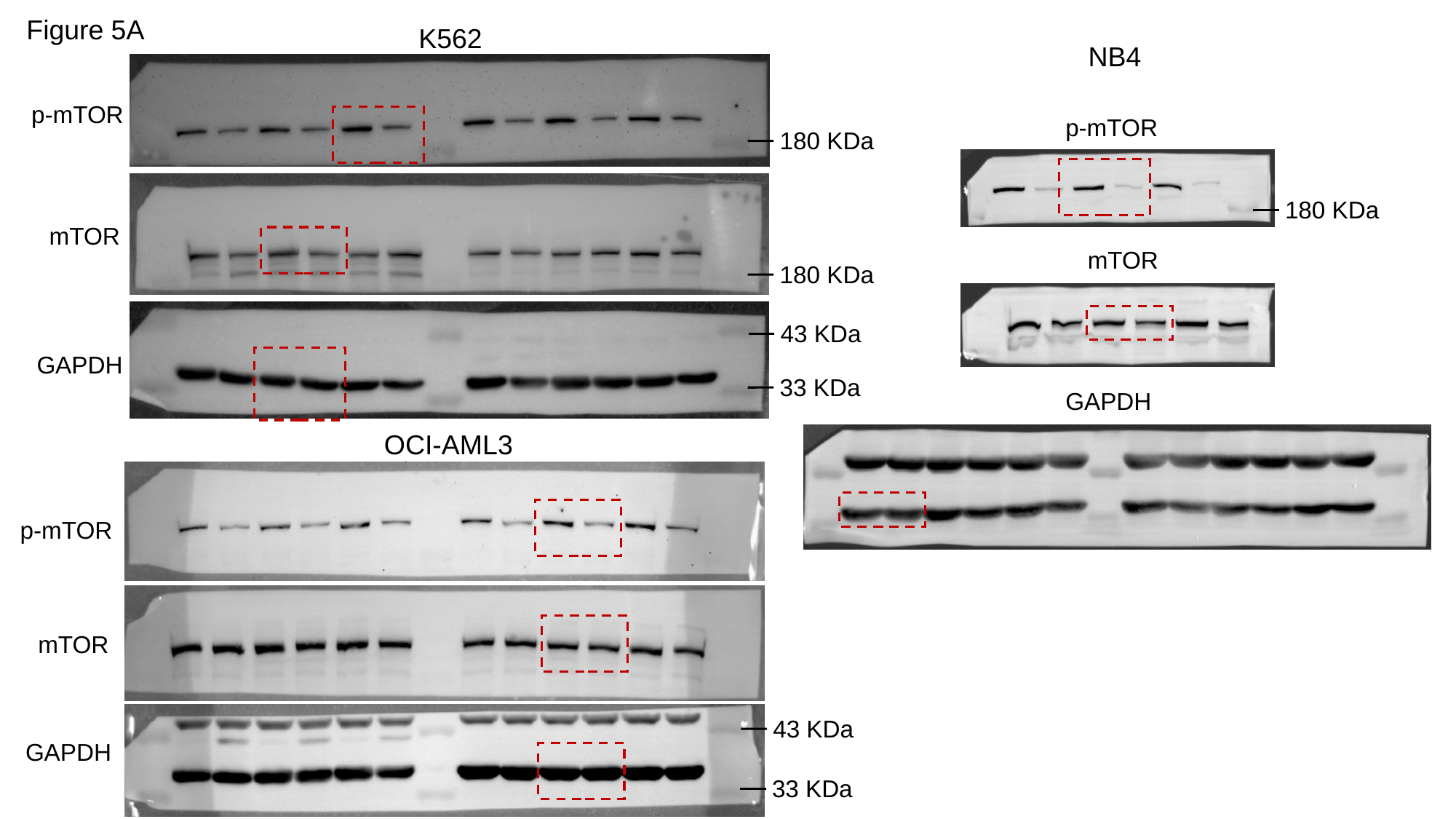

Figure 5A
K562
NB4
p-mTOR
p-mTOR
180 KDa
180 KDa
mTOR
mTOR
180 KDa
43 KDa
GAPDH
33 KDa
GAPDH
OCI-AML3
p-mTOR
mTOR
43 KDa
GAPDH
33 KDa

## Slide 16
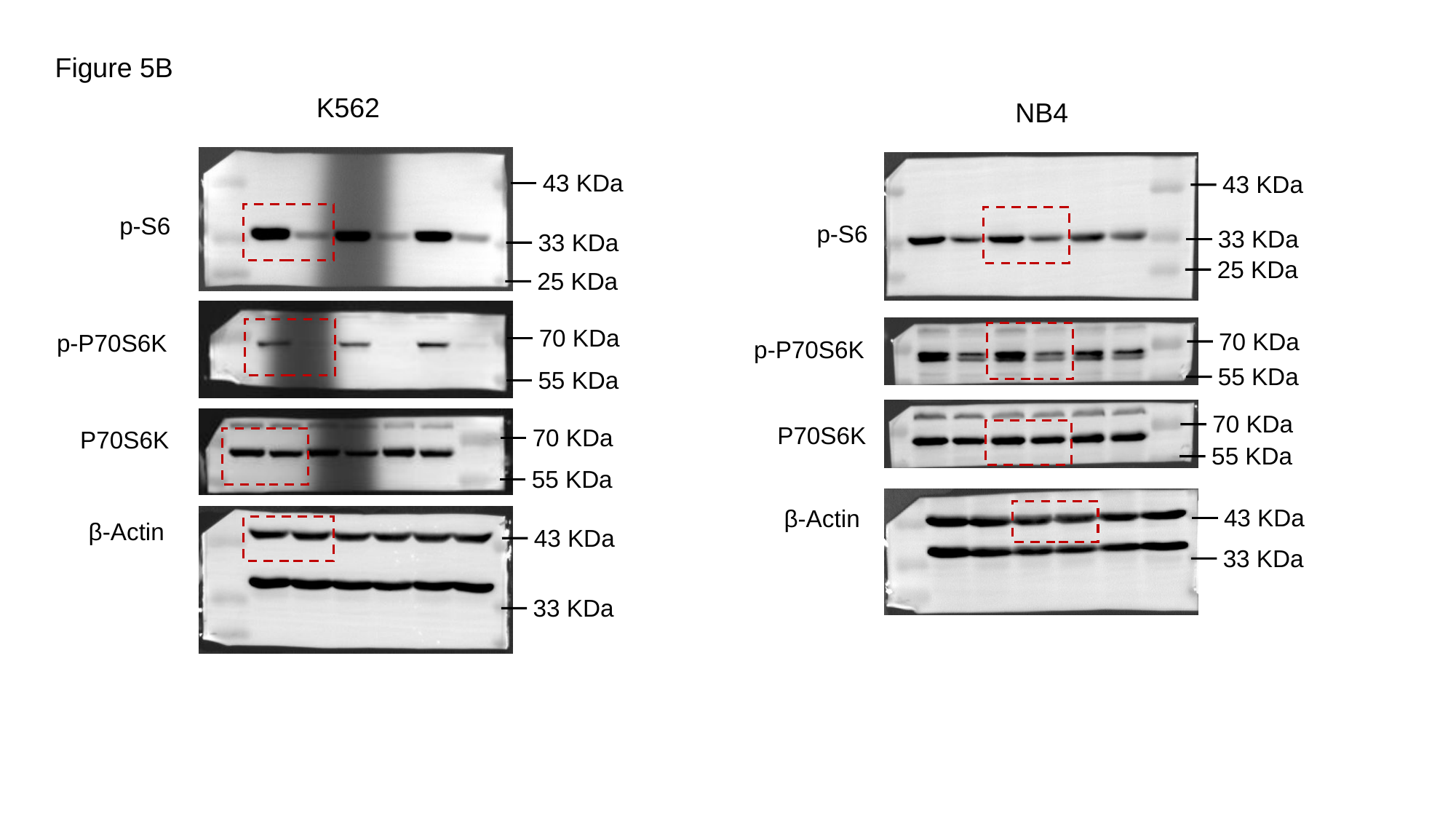

Figure 5B
K562
NB4
43 KDa
43 KDa
p-S6
p-S6
33 KDa
33 KDa
25 KDa
25 KDa
70 KDa
70 KDa
p-P70S6K
p-P70S6K
55 KDa
55 KDa
70 KDa
P70S6K
70 KDa
P70S6K
55 KDa
55 KDa
43 KDa
β-Actin
β-Actin
43 KDa
33 KDa
33 KDa

## Slide 17
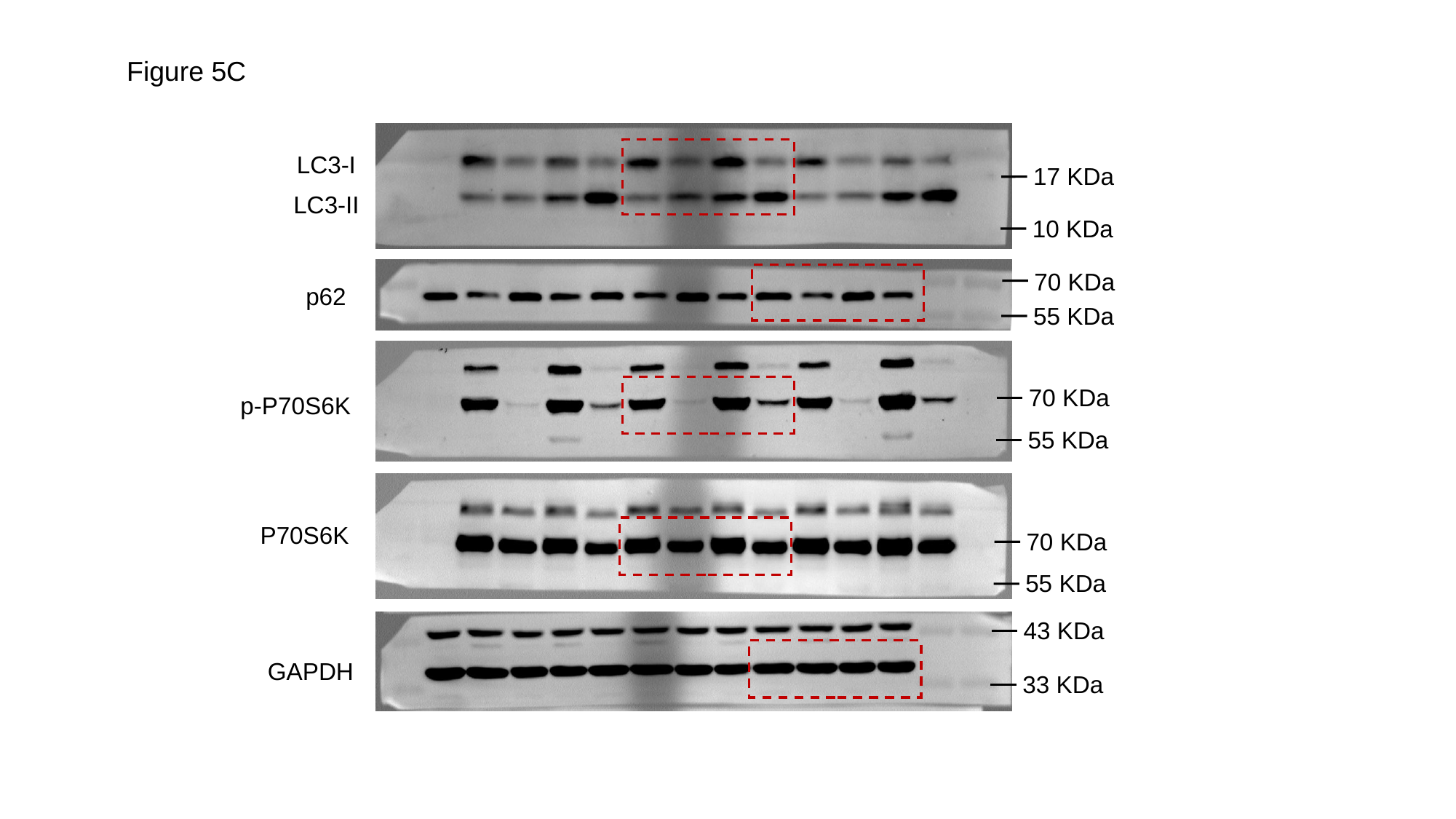

Figure 5C
LC3-I
17 KDa
LC3-II
10 KDa
70 KDa
p62
55 KDa
70 KDa
p-P70S6K
55 KDa
P70S6K
70 KDa
55 KDa
43 KDa
GAPDH
33 KDa

## Slide 18
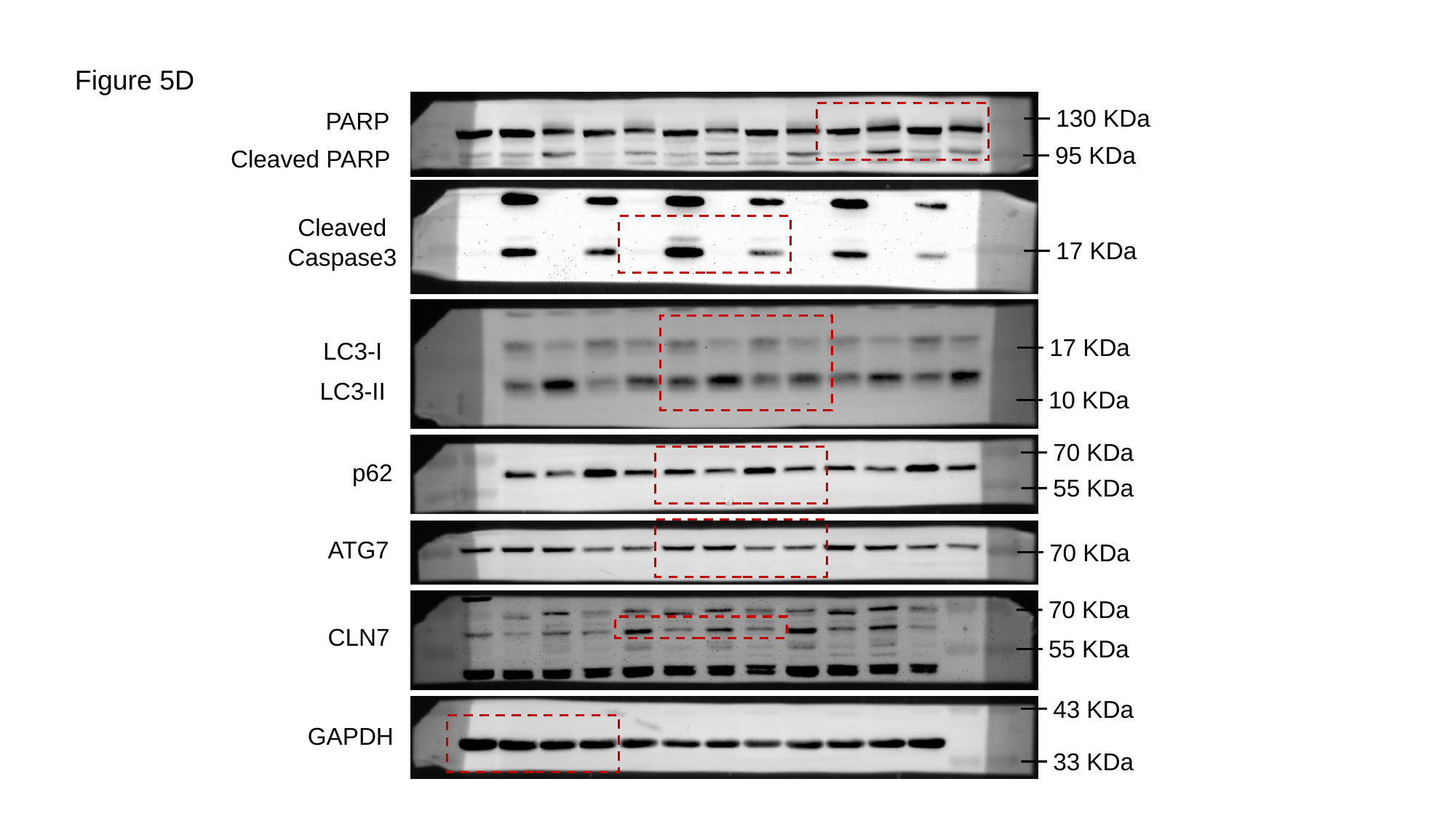

Figure 5D
130 KDa
PARP
95 KDa
Cleaved PARP
Cleaved Caspase3
17 KDa
17 KDa
LC3-I
LC3-II
10 KDa
70 KDa
p62
55 KDa
ATG7
70 KDa
70 KDa
CLN7
55 KDa
43 KDa
GAPDH
33 KDa

## Slide 19
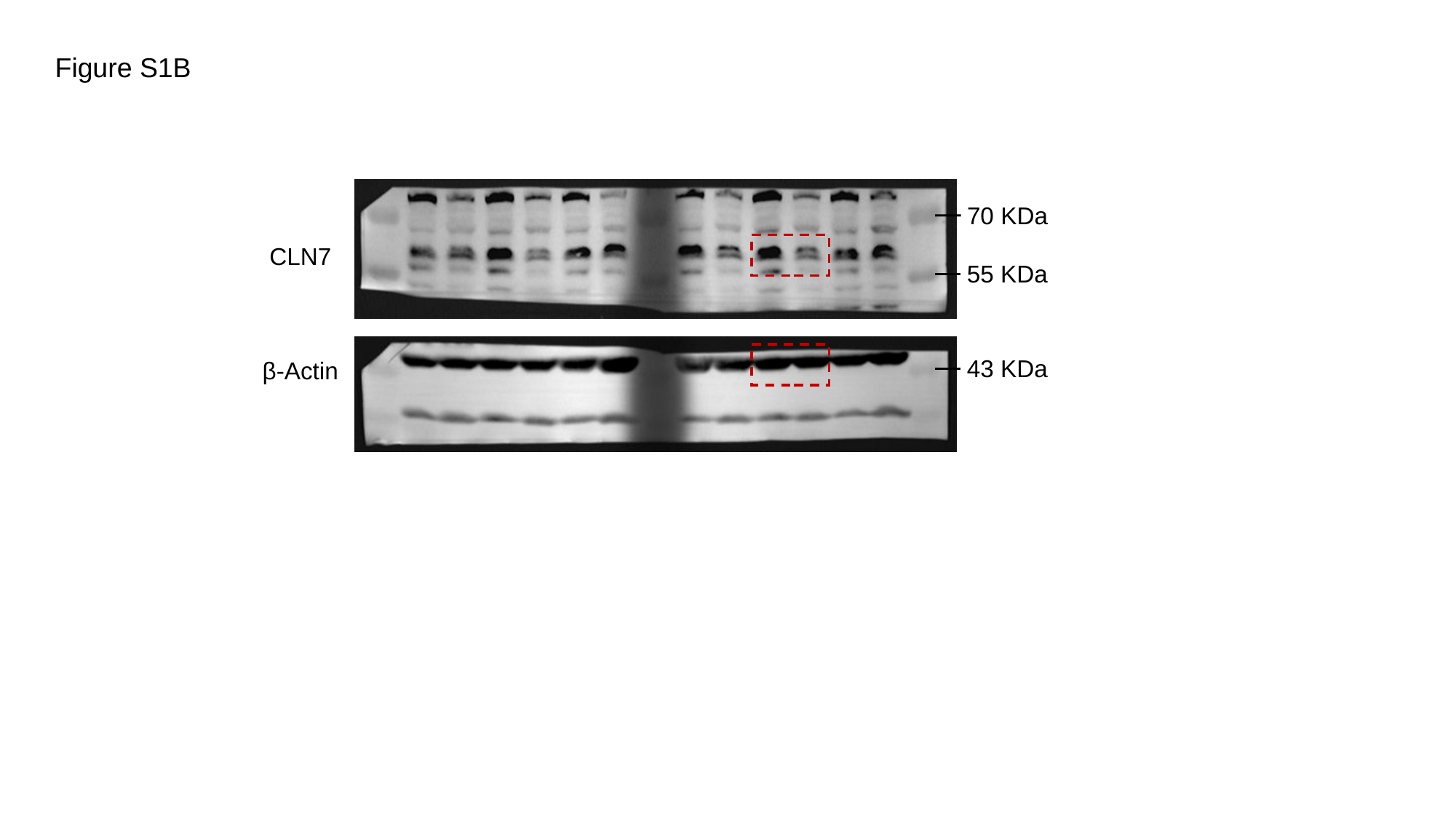

Figure S1B
70 KDa
CLN7
55 KDa
43 KDa
β-Actin

## Slide 20
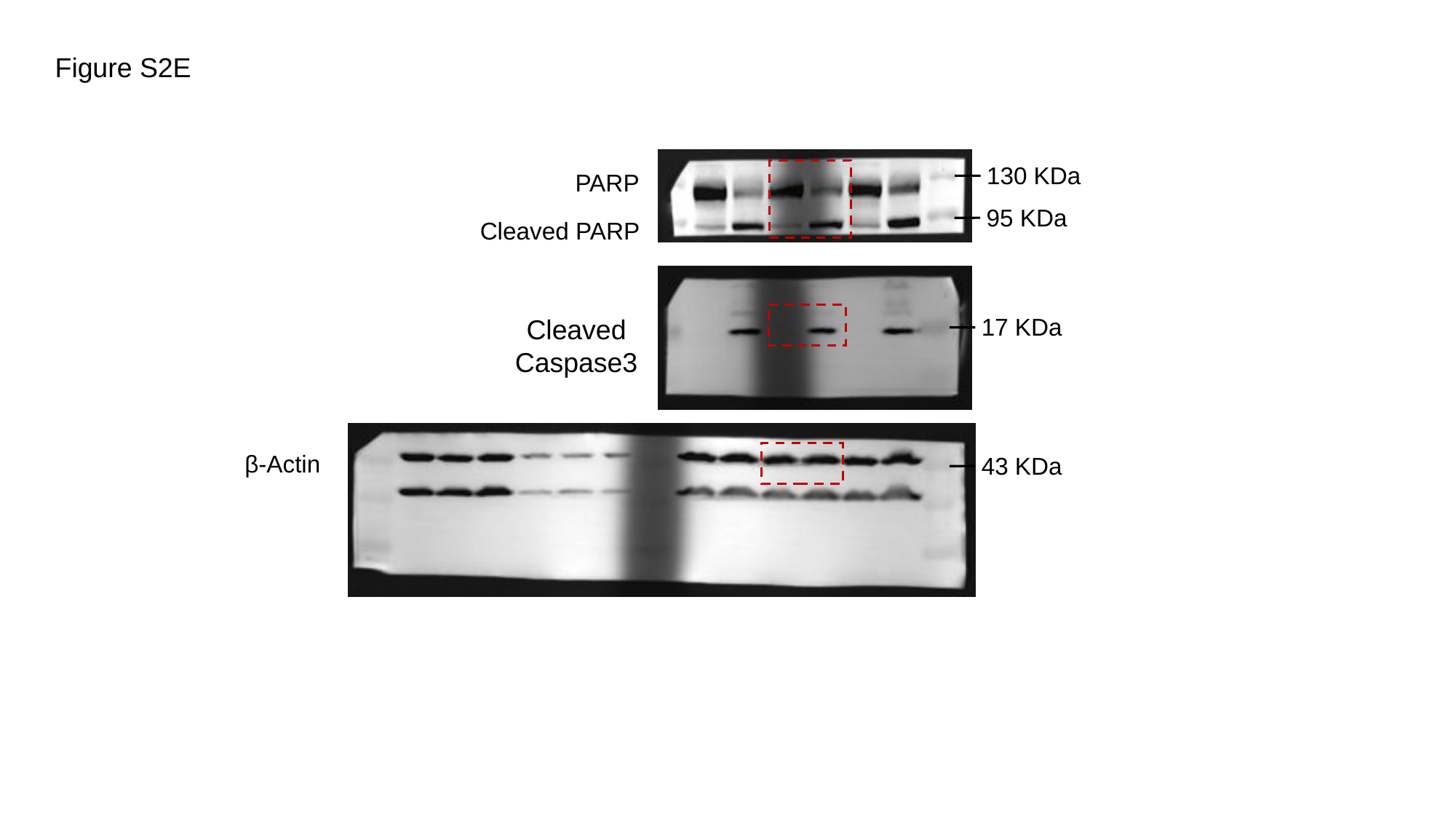

Figure S2E
130 KDa
PARP
95 KDa
Cleaved PARP
Cleaved Caspase3
17 KDa
β-Actin
43 KDa

## Slide 21
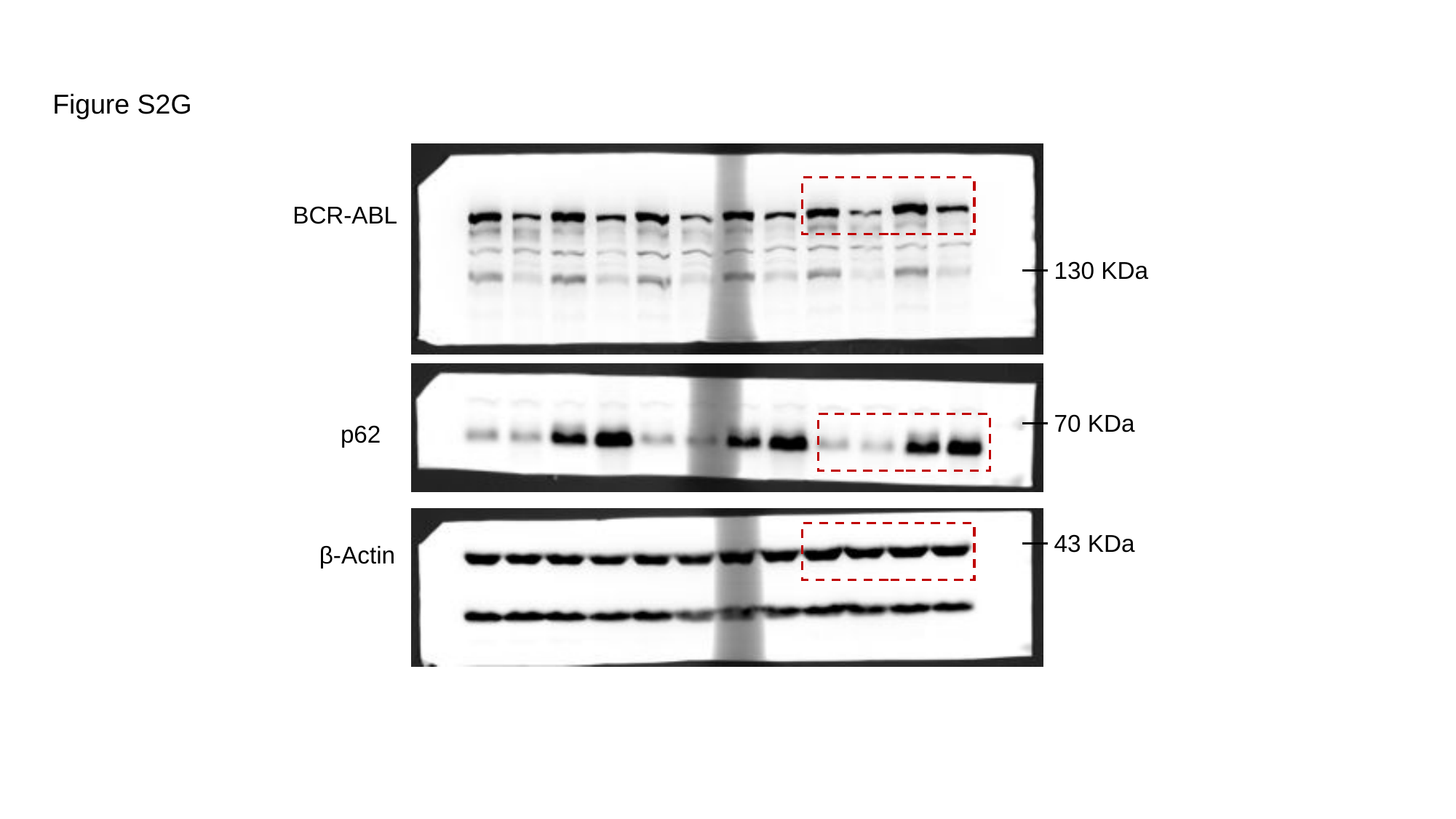

Figure S2G
BCR-ABL
130 KDa
70 KDa
p62
43 KDa
β-Actin

## Slide 22
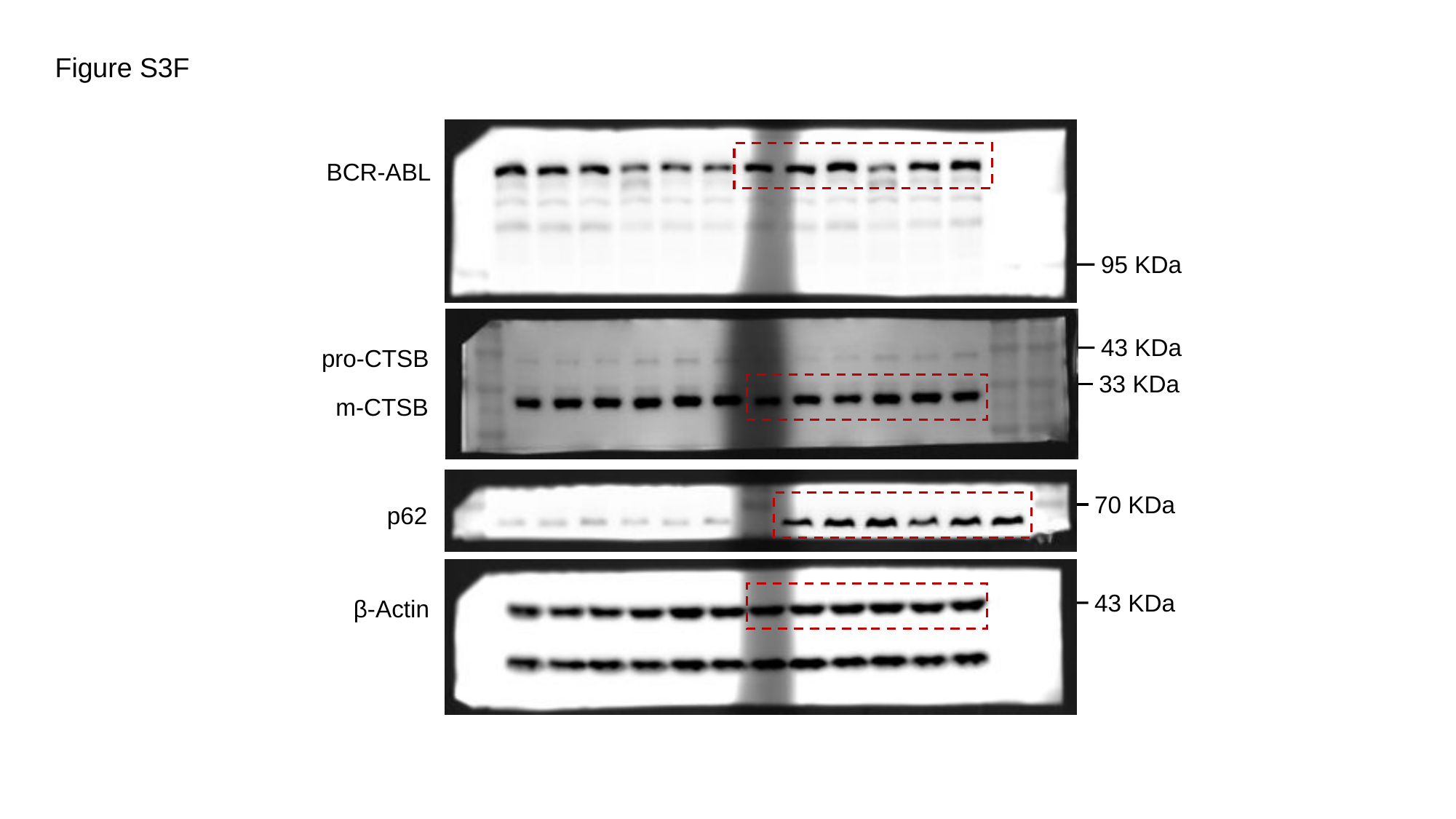

Figure S3F
BCR-ABL
95 KDa
43 KDa
pro-CTSB
33 KDa
m-CTSB
70 KDa
p62
43 KDa
β-Actin

## Slide 23
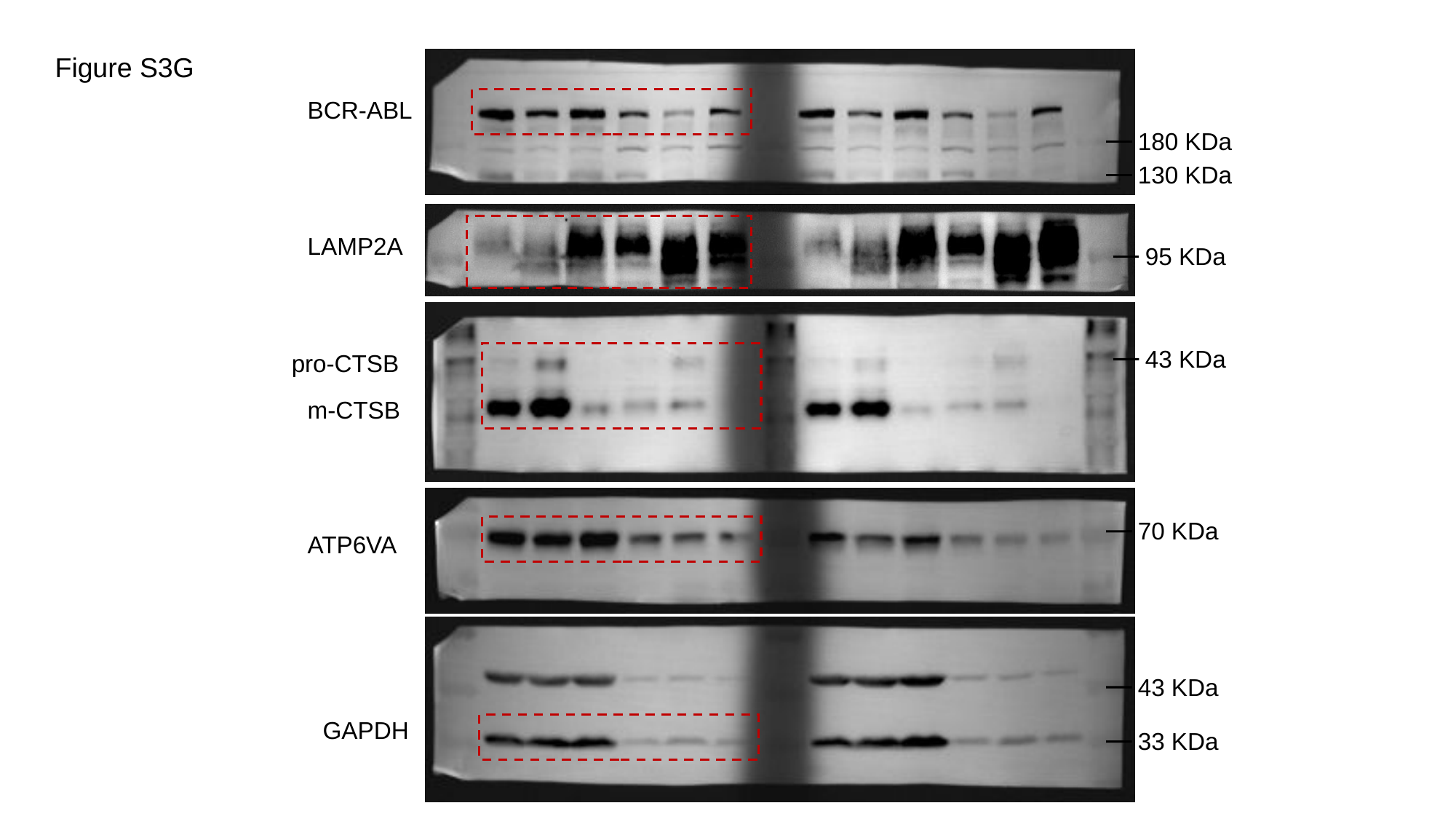

Figure S3G
BCR-ABL
180 KDa
130 KDa
LAMP2A
95 KDa
43 KDa
pro-CTSB
m-CTSB
70 KDa
ATP6VA
43 KDa
GAPDH
33 KDa

## Slide 24
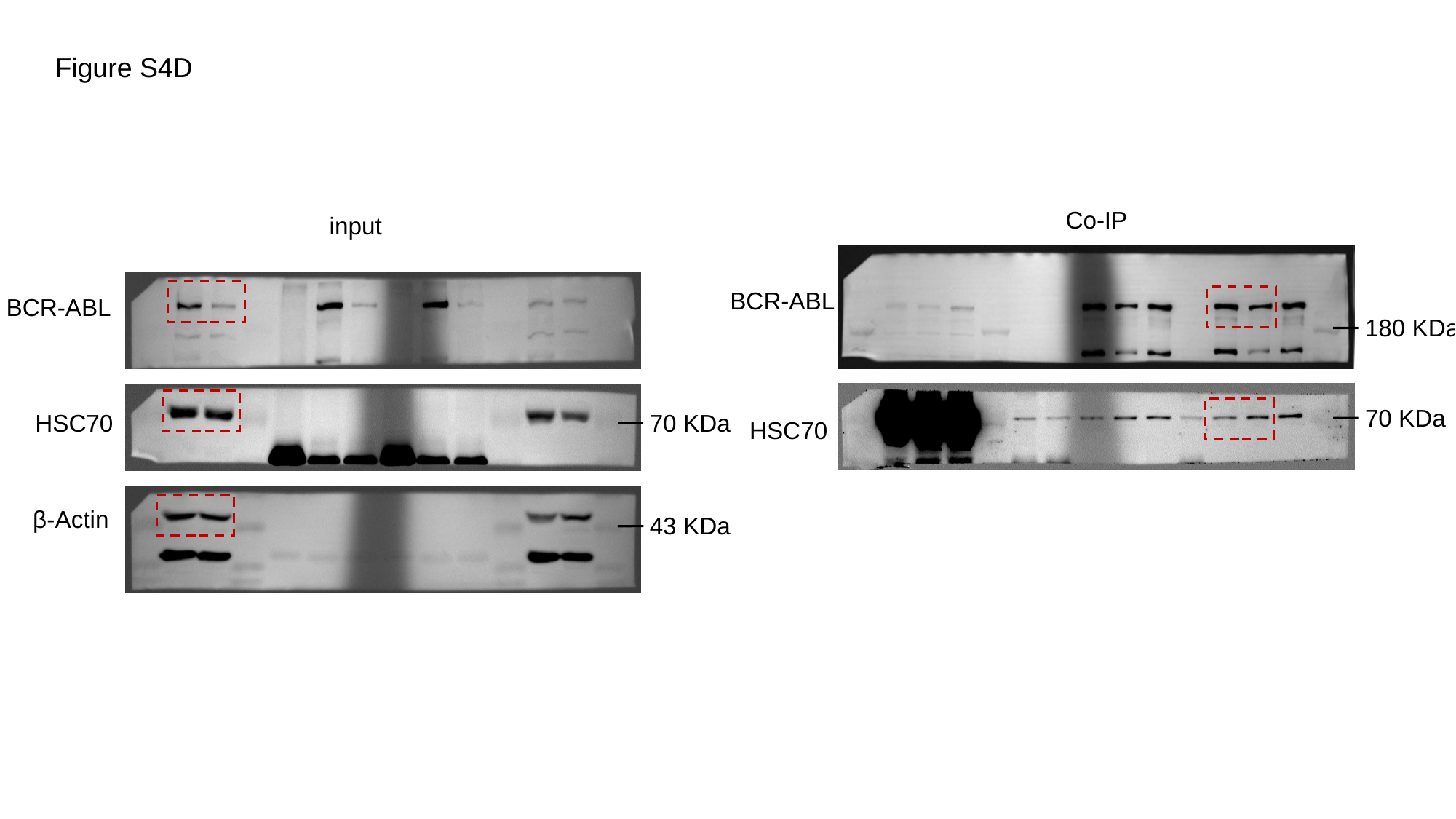

Figure S4D
Co-IP
input
BCR-ABL
BCR-ABL
180 KDa
70 KDa
HSC70
70 KDa
HSC70
β-Actin
43 KDa

## Slide 25
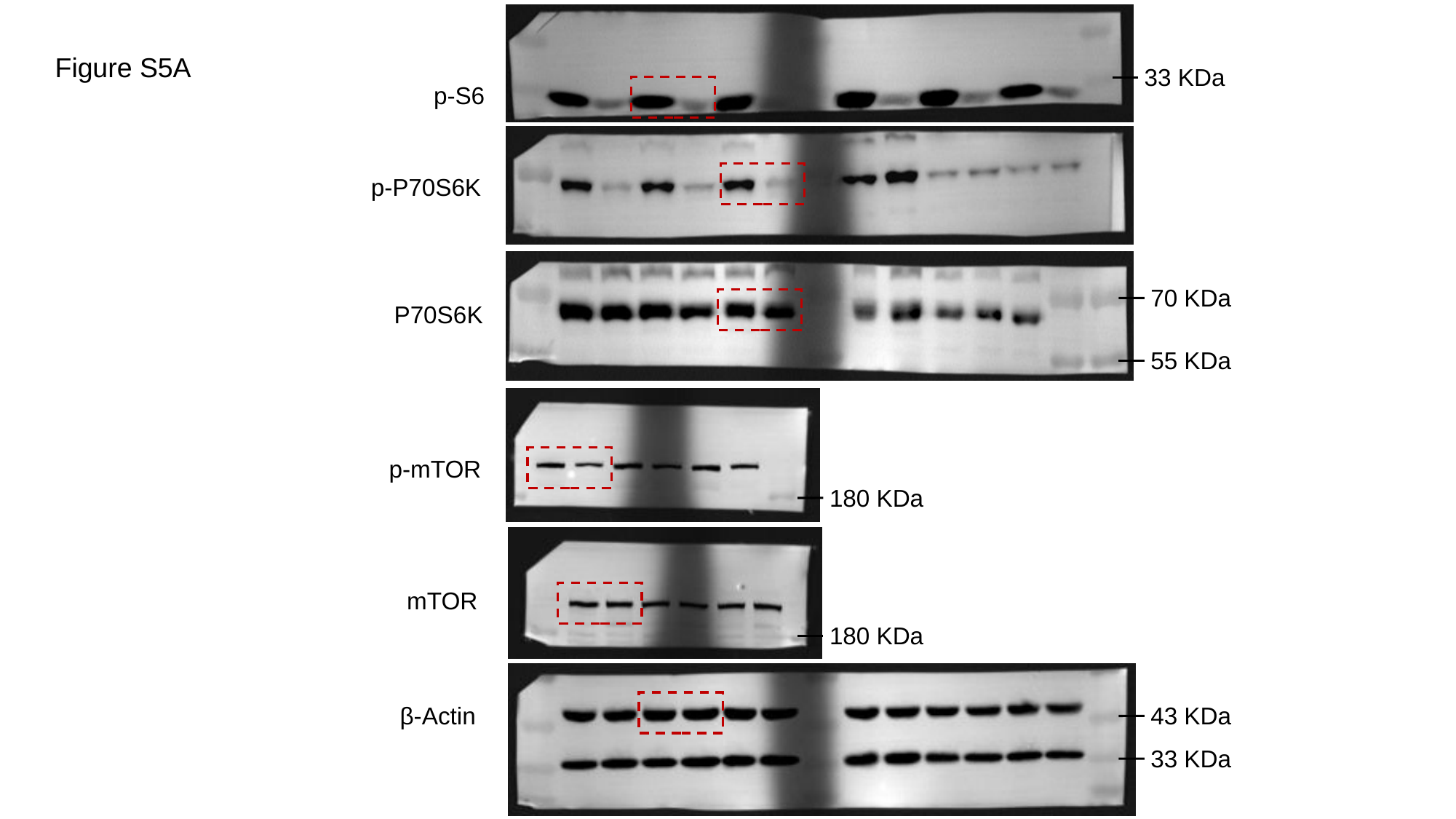

Figure S5A
33 KDa
p-S6
p-P70S6K
70 KDa
P70S6K
55 KDa
p-mTOR
180 KDa
mTOR
180 KDa
β-Actin
43 KDa
33 KDa

## Slide 26
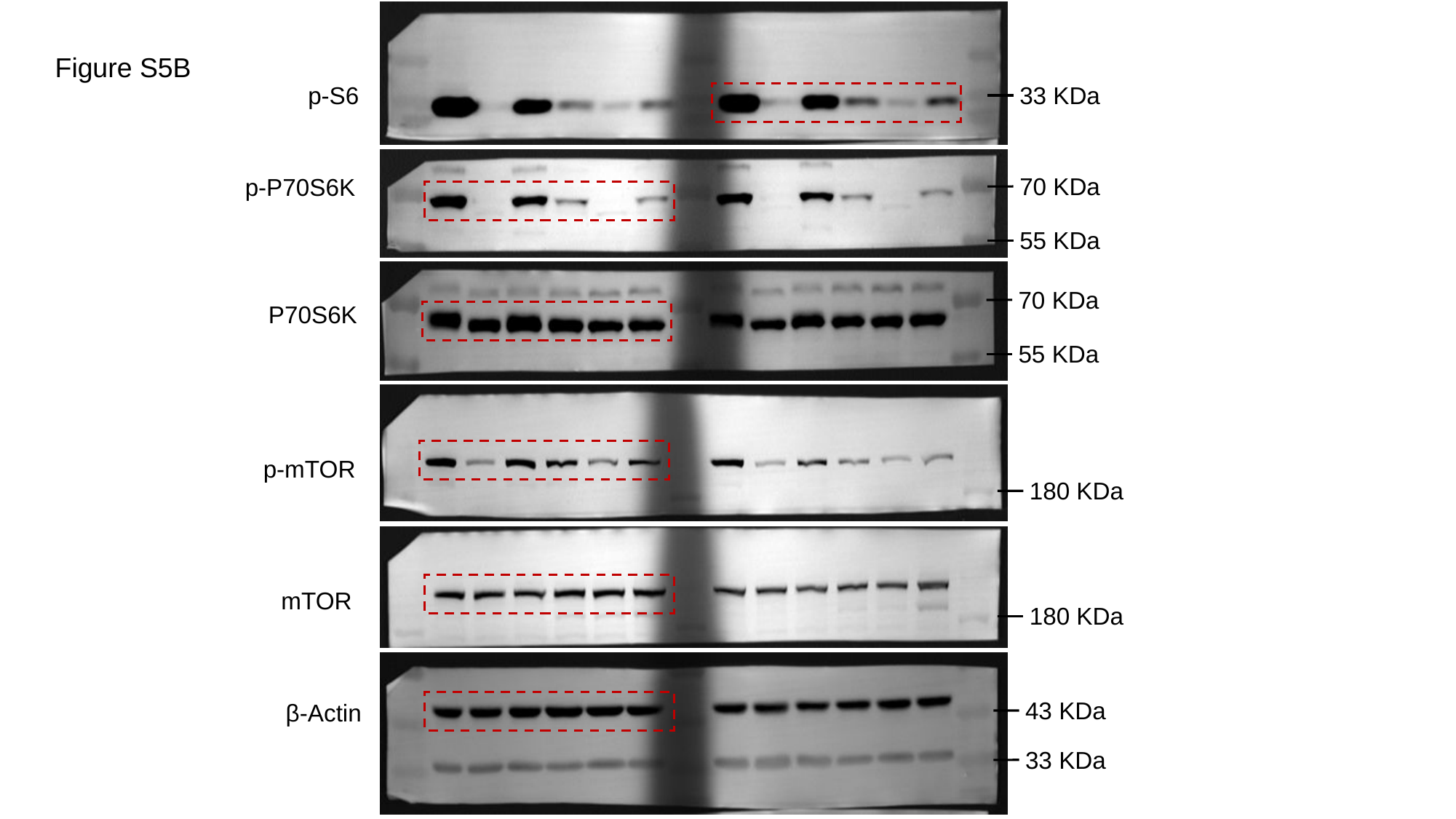

Figure S5B
p-S6
33 KDa
70 KDa
p-P70S6K
55 KDa
70 KDa
P70S6K
55 KDa
p-mTOR
180 KDa
mTOR
180 KDa
43 KDa
β-Actin
33 KDa

## Slide 27
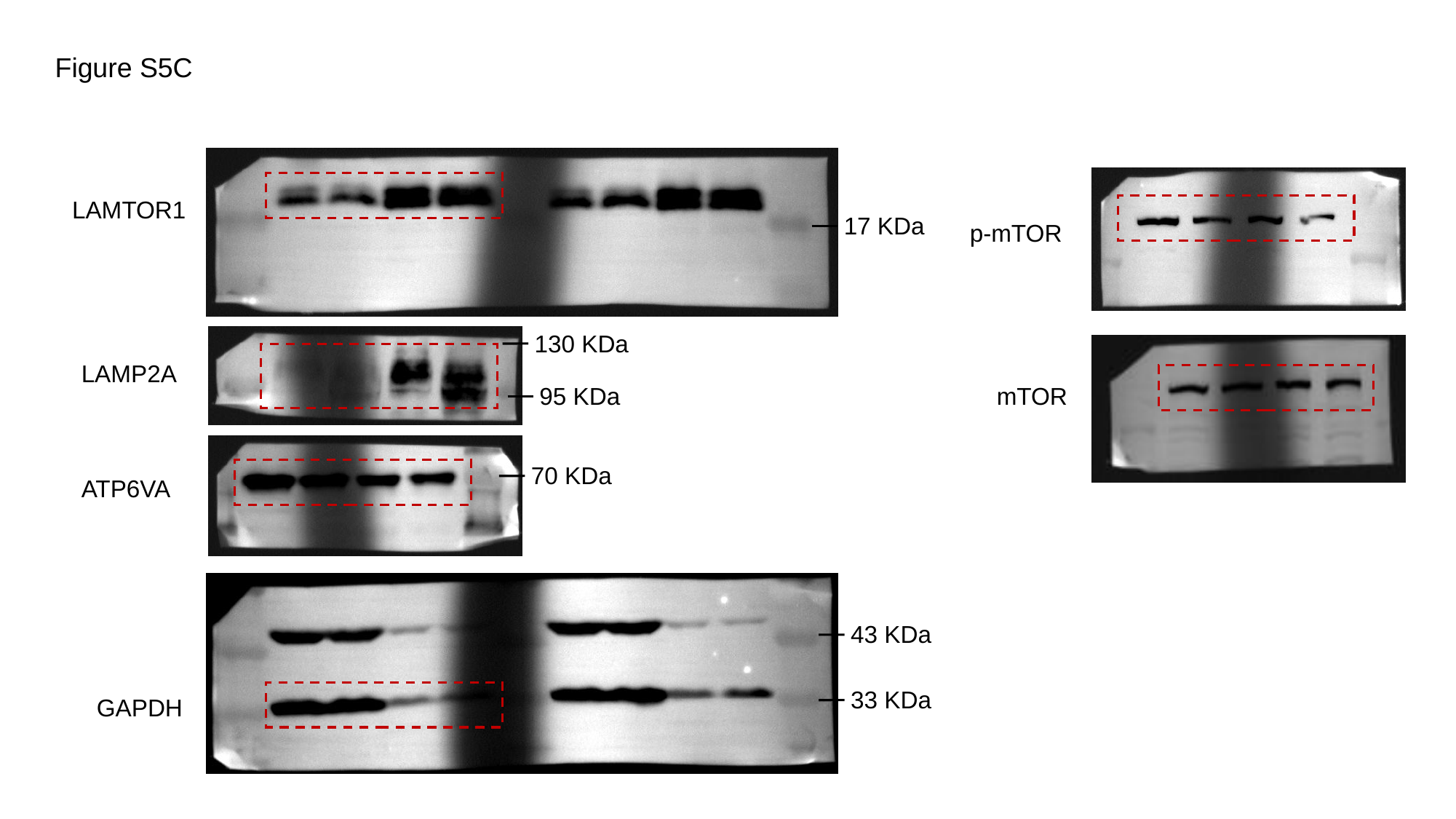

Figure S5C
LAMTOR1
17 KDa
p-mTOR
130 KDa
LAMP2A
95 KDa
mTOR
70 KDa
ATP6VA
43 KDa
33 KDa
GAPDH

## Slide 28
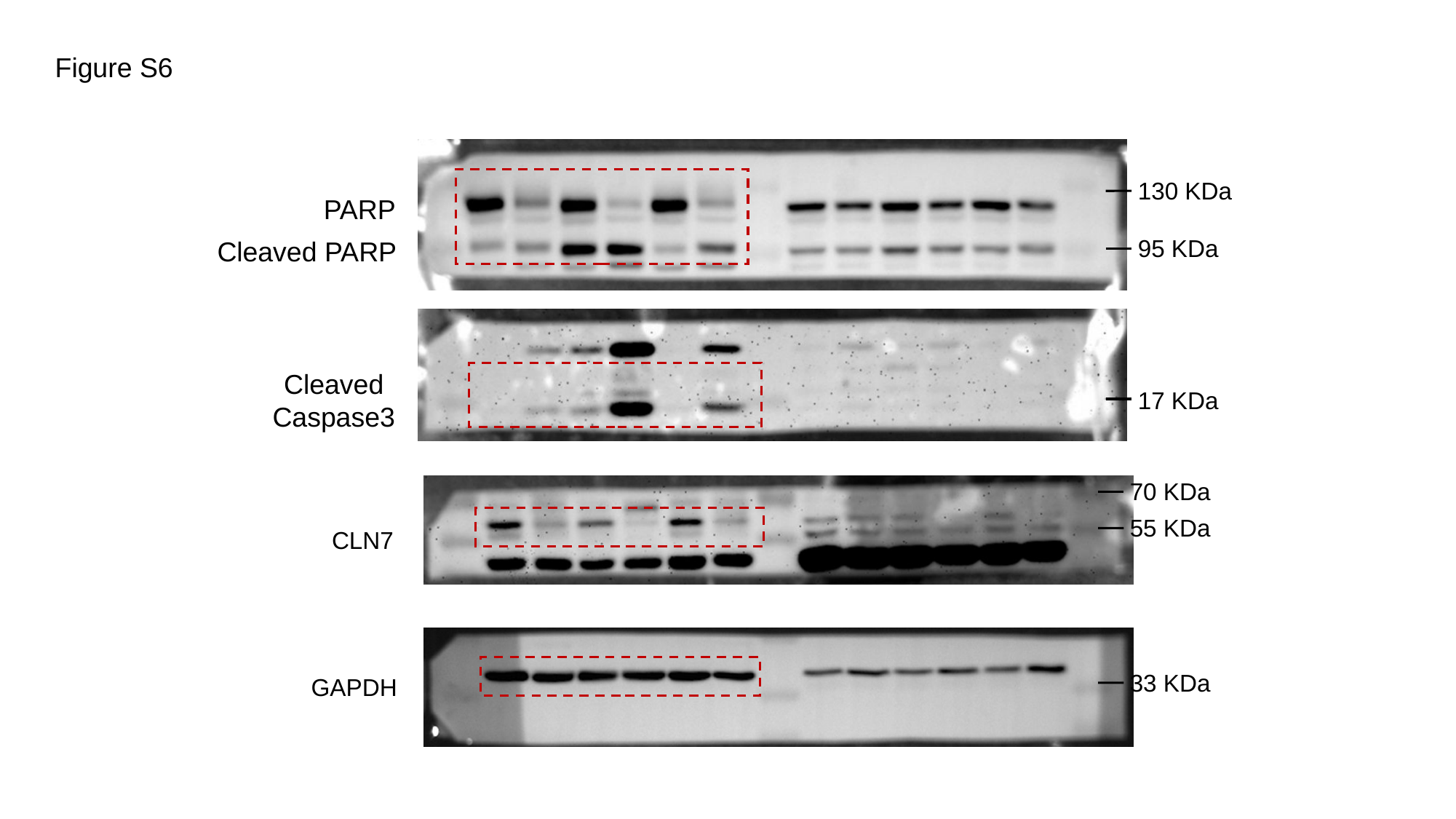

Figure S6
130 KDa
PARP
95 KDa
Cleaved PARP
Cleaved Caspase3
17 KDa
70 KDa
55 KDa
CLN7
33 KDa
GAPDH

## Slide 29
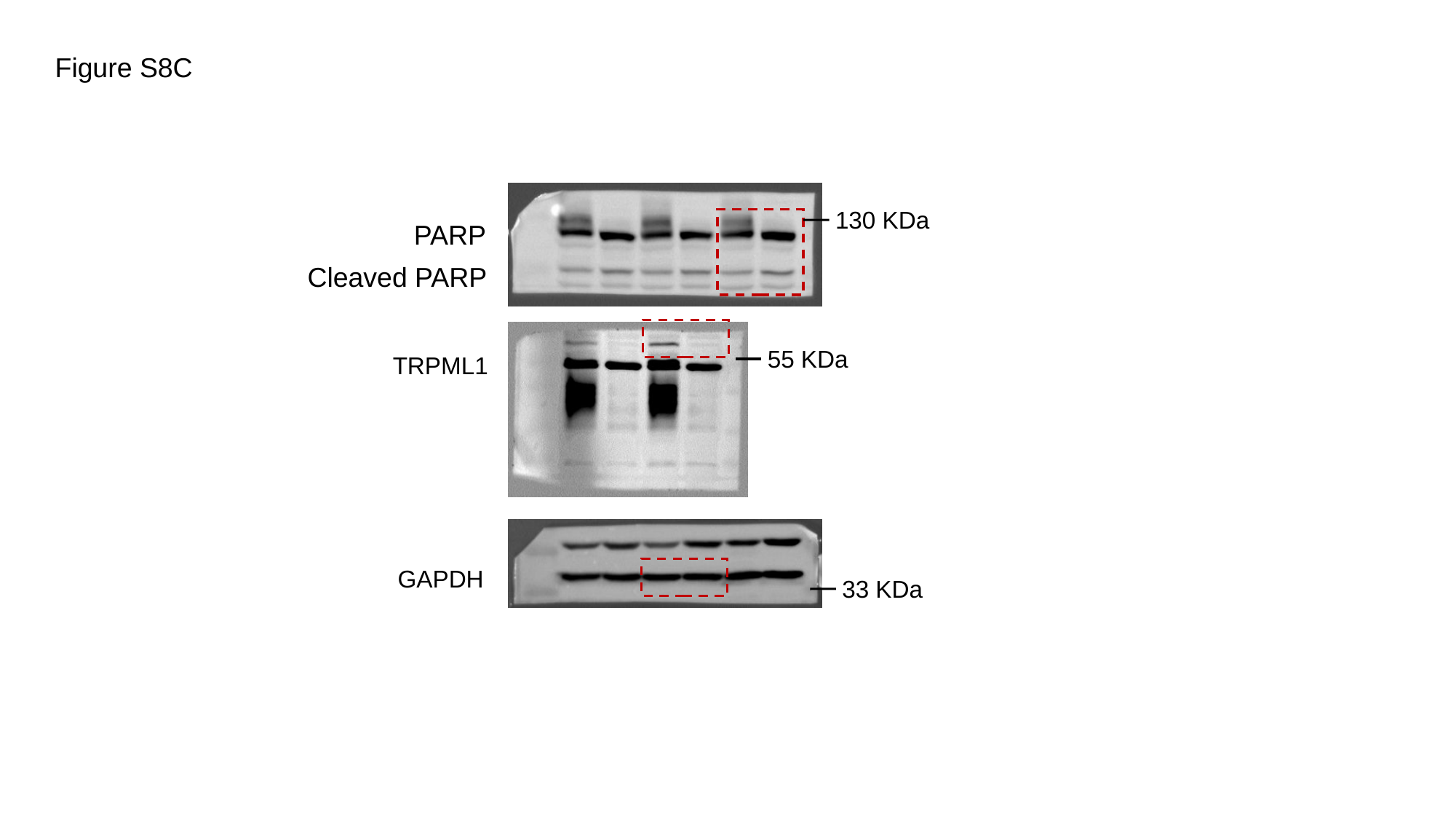

Figure S8C
130 KDa
PARP
Cleaved PARP
55 KDa
TRPML1
GAPDH
33 KDa

## Slide 30
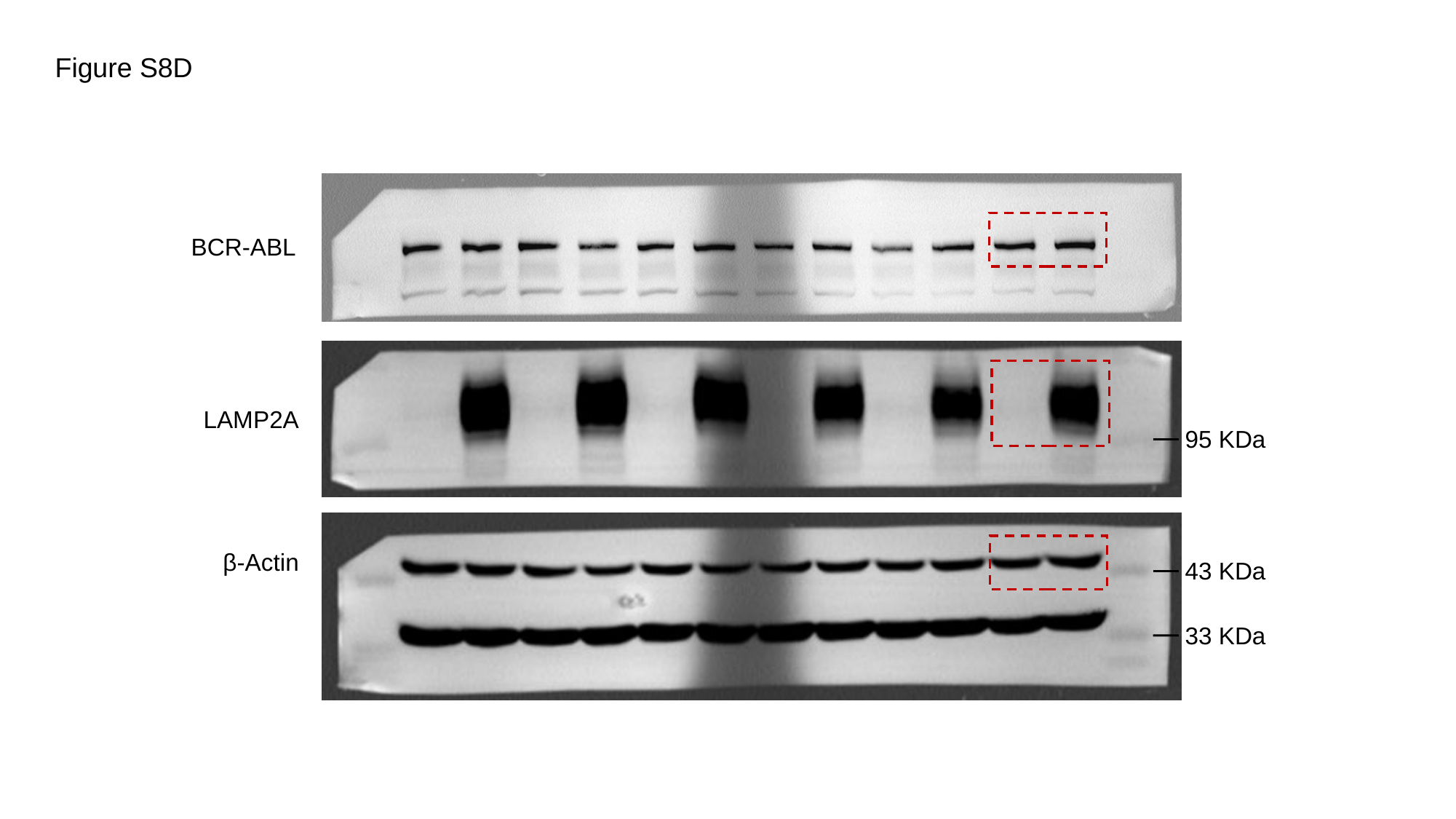

Figure S8D
BCR-ABL
LAMP2A
95 KDa
β-Actin
43 KDa
33 KDa
